# Loss of IGFBP-6 promotes monocyte-driven atherogenesis in periodontal disease

**DOI:** 10.1038/s41598-026-47023-6

**Published:** 2026-05-12

**Authors:** Dáire Shanahan, Georgios Kremastiotis, Kerry Wadey, Claudine Hodgson, Joon Seong, Nicola West, Angela H. Nobbs, Jason Johnson, Sarah George

**Affiliations:** 1 Laboratory of Cardiovascular Pathology, Translational Health Sciences, Bristol Medical School, University of Bristol, Level 7 Bristol Royal Infirmary, Bristol, UK; 2 Bristol Dental School, University of Bristol, 1 Trinity Quay, Avon Street, Bristol, BS2 0PT UK; 3 Bristol Dental School Research Laboratories, Dorothy Hodgkin Building, University of Bristol, Bristol, BS1 3NY UK

**Keywords:** IGFBP-6_1_, ABCG1_2_, Atherosclerosis_3_, Periodontitis_4_, Monocyte_5_, Macrophage_6_, Cell biology, Diseases, Immunology

## Abstract

Periodontitis has been associated with an increased risk of atherosclerosis, potentially through altered systemic monocyte and macrophage function. We aimed to identify a differentially expressed protein in naïve monocytes from individuals with periodontitis compared to controls and investigate its association with pro-atherosclerotic monocyte and macrophage functions. Peripheral blood monocytes were isolated from individuals with periodontitis and matched controls. Proteomic analysis and bioinformatics identified insulin-like growth factor-binding protein-6 (IGFBP-6) as a protein of interest. IGFBP-6 protein was quantified in monocytes, macrophages, foam cells, and plasma using Western blotting. To model periodontitis in vitro, monocytes and macrophages were treated with *Porphyromonas gingivalis* lipopolysaccharide. Extracellular vesicles were analysed for IGFBP-6 content and activity. Foam cell formation, cholesterol transporter expression, monocyte adhesion, macrophage transmigration, inflammatory cytokine production, and apoptosis were quantified. IGFBP-6 levels were reduced intracellularly but elevated in plasma in periodontitis, and similar patterns were observed in lipopolysaccharide-treated cells. IGFBP-6 in extracellular vesicles lacked biological activity. Reduced IGFBP-6 protein was associated with increased foam cell formation and downregulation of ATP-binding cassette subfamily-G1 (ABCG1), independent of IGF signalling. Restoration of IGFBP-6 was associated with reduced foam cell formation and inflammation, alongside inhibition of macrophage transmigration. These findings suggest that altered IGFBP-6 levels may contribute to the progression of atherosclerosis in periodontitis.

**Supplementary Information:**

The online version contains supplementary material available at 10.1038/s41598-026-47023-6.

## Introduction

Atherosclerosis is a chronic inflammatory disease characterised by the accumulation of lipids and immune cells within the arterial wall, leading to plaque formation that restricts blood flow and increases the risk of cardiovascular events^1,2^. Monocytes and their differentiated progeny, macrophages, are key drivers of atherogenesis^3^. Upon recruitment to activated endothelium, monocytes transmigrate into the subendothelial space, differentiate into macrophages, internalise modified low-density lipoproteins (LDLs), form foam cells, and sustain local inflammation through inflammatory mediator production, impaired efferocytosis and apoptosis^3–5^.

Periodontitis is a prevalent chronic inflammatory disease of the supporting structures of the teeth^6^. It arises from a dysregulated host-microbial interaction and is increasingly recognised as a contributor to systemic inflammation^7^. Epidemiological studies have linked periodontitis to increased risk of cardiovascular disease, and mechanistic evidence suggests that immune dysregulation caused by periodontitis may promote atherosclerosis^7–12^. However, the precise molecular pathways linking atherosclerosis and periodontitis remain incompletely defined, particularly with regard to monocyte and macrophage function. Peripheral blood monocytes are of particular relevance, as periodontal bacteria and their inflammatory components can enter the systemic circulation, where they interact with and activate circulating monocytes^7^, potentially shaping their subsequent contribution to vascular inflammation and atherogenesis. To address this, the initial aim of this study was to use proteomic analysis to identify dysregulated proteins in monocytes from individuals with periodontitis. This approach highlighted insulin-like growth factor-binding protein-6 (IGFBP-6) as a protein of interest, which subsequently became the primary focus of downstream functional investigations.

The insulin-like growth factor (IGF) system regulates growth, differentiation, and proliferation of many different types of cells, and comprises of IGF-1, IGF-2, their receptors, and six IGF binding proteins^13,14^. IGFBP-6 is unique among these for its strong binding preference for IGF-2, acting as a specific inhibitor of IGF-2 mediated actions^15^. In addition to modulating IGF availability, IGFBP-6 also exerts IGF-independent actions, including regulation of cell migration, apoptosis, and inflammation in various cell types^16–18^. Recent studies indicate that IGFBP-6 may play a protective role in vascular inflammation, with a reduced presence observed in unstable human plaques, and its deletion shown to worsen atherosclerosis and vascular inflammation in mouse models^19,20^.

Given that periodontitis is associated with systemic alterations in monocyte phenotype^21–23^, this study specifically investigated whether IGFBP-6 is associated with systemic monocyte/macrophage behaviour relevant to atherogenesis, including migration, foam cell formation and inflammatory mediator production. These effects were determined using proteomic profiling, endothelial cell adhesion and invasion assays, foam cell formation assessment, inflammatory mediator analysis, and apoptosis assays.

## Results

### Proteomic comparison of naïve monocytes from individuals with periodontitis and healthy controls

To determine whether periodontitis is associated with systemic alterations in circulating monocytes that may influence atherogenic behaviour, an unbiased proteomic comparison of naïve monocytes from individuals with periodontitis and matched healthy controls was performed. The aim of this analysis was to identify differentially expressed proteins and pathways that could reveal disease-related functional reprogramming and highlight candidate molecules for downstream mechanistic investigation.

**Fig. 1:**
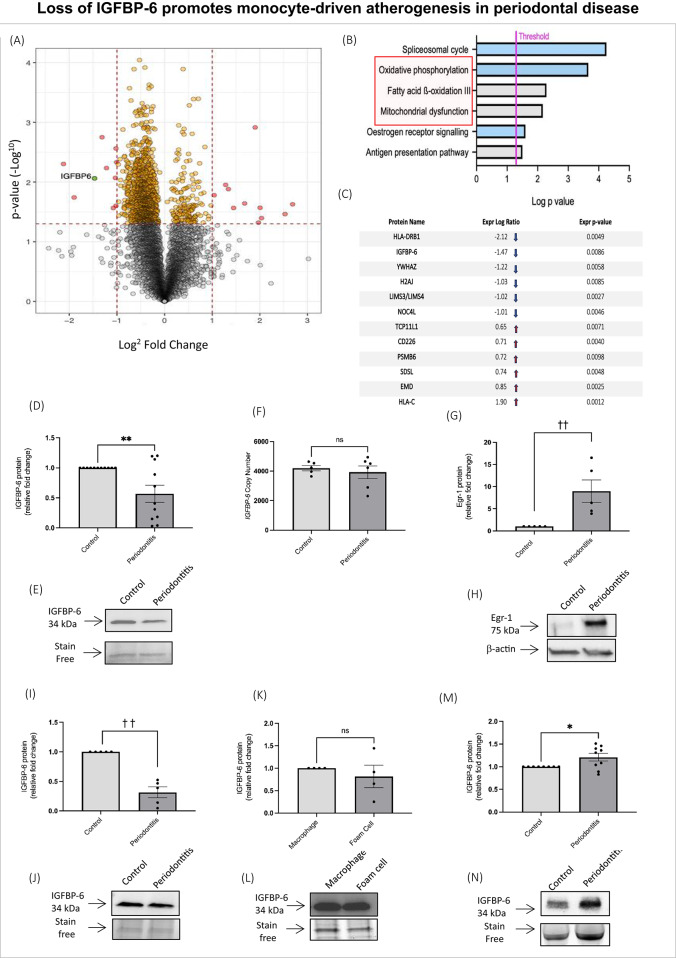
Proteomic and molecular comparison of IGFBP-6 in naïve monocytes and macrophages from individuals with periodontitis and controls.**(A)** Volcano plot of proteins identified in naïve monocytes from individuals with periodontitis compared to age- and sex-matched healthy controls (*n* = 15/group). Vertical red lines denote log2 fold change ± 1, and the horizontal red line indicates *p* = 0.05. Grey dots represent proteins that are not significantly different between groups; yellow dots indicate *p* < 0.05; red dots indicate *p* < 0.05 with log2 fold change > 1; green dot indicates IGFBP-6 protein. **(B)** Canonical pathway enrichment of differentially expressed proteins using IPA (top six pathways shown); bars represent -log(p-value) and predicted z-score. Blue bars indicate predicted inhibition; grey bars indicate no activity prediction. A purple line marks the significance threshold. Red box indicates cell metabolism-related pathways. **(C)** Top six up- and down-regulated “analysis ready molecules” identified by IPA Core Analysis (*p* < 0.01).**(D)** Quantification of IGFBP-6 protein in naïve monocytes from individuals with periodontitis and healthy controls (*p* = 0.0064; *n* = 11) with **(E)** representative Western blot. **(F)** Quantification of IGFBP-6 mRNA in naïve monocytes from individuals with periodontitis and matched healthy controls by qPCR (*p* = 0.4069; *n* = 6). **(G)** Quantification of Egr-1 protein in naïve monocytes from individuals with periodontitis and healthy controls (*p* = 0.0079; *n* = 5) with **(H)** representative Western blot. **(I)** Quantification of IGFBP-6 protein in macrophages from individuals with periodontitis and healthy controls (*p* = 0.0079; *n* = 5) with **(J)** representative Western blot. **(K)** Quantification of IGFBP-6 protein in macrophages and foam cells from individuals with periodontitis (*p* = 0.6250; *n* = 4) with **(L)** representative Western blot. **(M)** Quantification of IGFBP-6 protein in plasma from individuals with periodontitis and healthy controls (*p* = 0.0479, *n* = 8 periodontitis, *n* = 9 controls) with **(N)** representative Western blot. Protein expression was quantified by densitometry and normalised using stain-free gel technology or β-actin. Protein expression is displayed in fold change relative to control. *ns*, not significant, *p < 0.05, **p < 0.01 using two-tailed, unpaired t-test. †*p* < 0.05, ††*p* < 0.01 using Mann-Whitney t-test. Error bars indicate SEM. Arrowheads indicate the detected protein bands; molecular weights are noted in kDa. Refer to Figures S1-S5 for full length Western blots and gels.

Quantitative proteomic analysis revealed extensive differences in the abundance of proteins between naïve monocytes isolated from individuals with periodontitis and age- and sex-matched healthy controls. Peripheral blood was obtained from 15 participants per group (9 female, 6 male). The mean age ± standard deviation of the control group was 43.40 ± 10.23 years compared to 46.67 ± 8.42 years in the periodontitis group (*p* = 0.3778), and monocytes were isolated by adhesion. Donor demographic and clinical characteristics, including age, sex, ethnicity, smoking status, and periodontitis classification, are provided in Tables S1 and S2. The cells were lysed and analysed using Nano-LC Mass Spectrometry. This identified 7,334 proteins (excluding 53 common contaminants). Of the identified proteins, 1,124 proteins were significantly differentially expressed, with 969 downregulated and 155 upregulated in monocytes from individuals with periodontitis compared to the healthy controls (290 proteins at *p* < 0.01; 834 at *p* < 0.05). The distribution and magnitude of these alterations are illustrated in the volcano plot in Fig. 1A.

Ingenuity Pathway Analysis (IPA) was used to interpret the biological relevance of these proteomic changes. With a significance threshold of *p* < 0.01, log₂ fold-change > 1.3, and z-score > 2 or < 2, the top six enriched canonical pathways were identified. Notably, three of these were associated with cell metabolism (oxidative phosphorylation, fatty acid β-oxidation, and mitochondrial dysfunction), highlighting metabolic reprogramming as a key feature in monocytes from individuals with periodontitis (Fig. 1B). To identify candidate proteins for further validation, differentially expressed proteins were ranked by fold change and significance (Fig. 1C). Although several human leukocyte antigen (HLA) proteins showed marked differential expression, their established role in antigen presentation made them less relevant to the inflammatory and metabolic pathways of interest. Among the remaining proteins, IGFBP-6 emerged as a protein of interest, as IGFBP-6 has been found to be downregulated in atherosclerotic plaques^20,24^, upregulated in stable human coronary plaques^25^, and co-localised with macrophages between the fibrous cap and the necrotic core^20^, suggesting it may play a role in the pathogenesis of atherosclerosis.

## IGFBP-6 protein abundance was altered in monocytes, macrophages, and plasma from individuals with periodontitis

To validate the proteomic findings, IGFBP-6 protein abundance was assessed in naïve monocytes by Western blotting. A significant 43% reduction in IGFBP-6 protein was observed in monocytes from people with periodontitis compared to healthy controls (*p* = 0.0064), confirming the proteomic data (Fig. 1D and E). Interestingly, *IGFBP-6* mRNA levels were not significantly different between the two groups (*p* > 0.9999), indicating a possible post-transcriptional mechanism of regulation (Fig. 1F). To evaluate downstream effects of differential IGFBP-6 protein levels, Egr-1 protein was also quantified. Egr-1, a transcription factor inhibited by IGFBP-6 and regulated by insulin-like growth factor 1 receptor (IGF-1R)^26^, was significantly increased in monocytes from individuals with periodontitis (*p* = 0.0079; Fig. 1G and H), suggesting functional consequences of reduced IGFBP-6 protein.

To investigate the abundance of IGFBP-6 protein during monocyte-to-macrophage differentiation, protein levels were assessed in macrophages derived from peripheral blood. IGFBP-6 protein levels remained significantly reduced in macrophages from individuals with periodontitis compared to controls (*p* < 0.0001; Fig. 1I and J). However, no significant differences in IGFBP-6 levels were observed between macrophages and foam cells (*p* > 0.6250; Fig. 1K and L), indicating that lipid loading does not further influence IGFBP-6 protein expression at this differentiation stage.

In contrast to monocytes and macrophages, plasma IGFBP-6 protein was significantly elevated in individuals with periodontitis compared to controls (Fig. 1M and N).

### Stimulation with ***P. gingivalis*** LPS modulated IGFBP-6 and Egr-1 protein levels in monocytes and macrophages

**Fig. 2:**
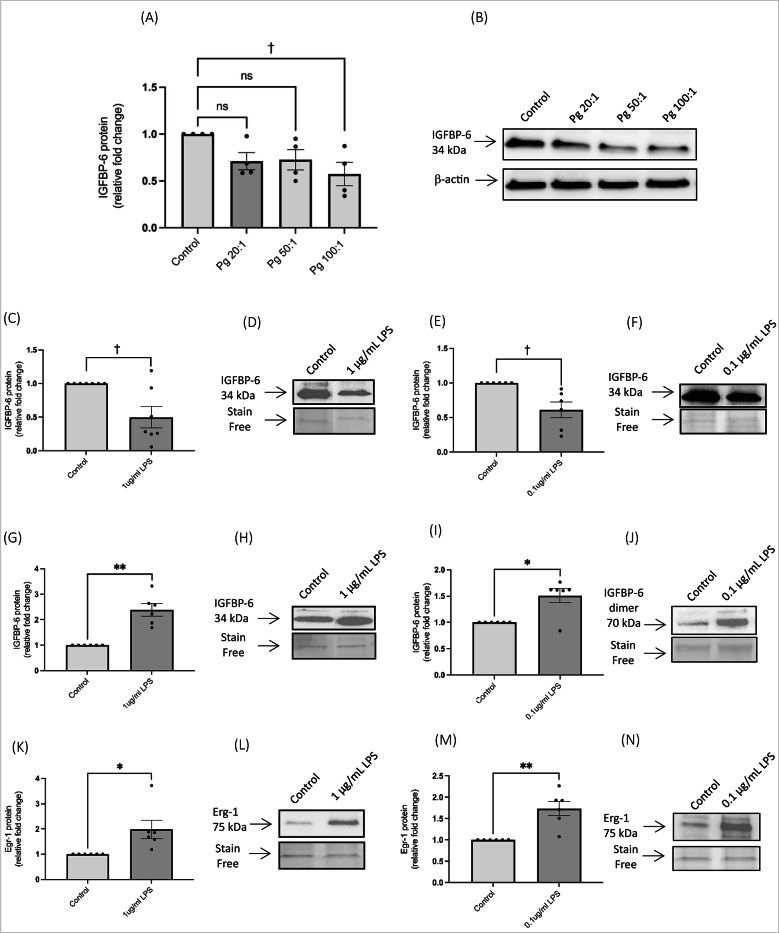
**Microbial stimulation modulates IGFBP-6 and Egr-1 protein expression in monocytes and macrophages from healthy controls.** Naïve monocytes isolated from healthy individuals were initially exposed to live *P. gingivalis* (W83) at bacteria: monocyte ratios of 20:1, 50:1 or 100:1 for 2 h. Cell lysates were collected and analysed by Western blotting. IGFBP-6 protein was quantified in cell lysates from **(A)** monocytes (*p* = 0.0190, *n* = 4) with **(B)** representative Western blots shown. Naïve monocytes and macrophages isolated from healthy individuals were treated with 0.1–1 µg/mL *P. gingivalis* LPS, respectively, for 2 h in serum-free RPMI (monocytes) or RPMI supplemented with 40 ng/mL M-CSF (macrophages). Cell lysates and conditioned media were collected and analysed by Western blotting. IGFBP-6 protein was quantified in cell lysates from **(C)** monocytes (*p* = 0.0469, *n* = 7) or **(E)** macrophages (*p* = 0.0312, *n* = 6), with **(D**,** F)** representative Western blots shown. IGFBP-6 protein was quantified in conditioned media from **(G)** monocytes (*p* = 0.0022, *n* = 6) or **(I)** macrophages (*p* = 0.0135, *n* = 6), with **(H**,** J)** representative Western blots shown. Egr-1 protein was quantified in cell lysates from **(K)** monocytes (*p* = 0.0421, *n* = 6) or **(M)** macrophages (*p* = 0.0070, *n* = 6), with **(L**,** N)** representative Western blots shown. IGFBP-6 was detected as a dimer in macrophage media. Densitometric scanning of protein bands was normalised by stain free gel technology or β-actin and protein expression is displayed in fold change relative to control. *p < 0.05, **p < 0.01, using two-tailed, paired t-test; †*p* < 0.05, using Wilcoxon matched-pairs t-test or Friedman’s test with Dunn’s *post hoc* test for non-parametric data. Error bars indicate SEM. Arrowheads indicate detected protein bands; molecular weights are shown in kDa. Refer to Figures S6-S12 for full length Western blots and gels.

To determine whether whole bacterial exposure modulates IGFBP-6 abundance, naïve monocytes from healthy individuals were stimulated with live *Porphyromonas gingivalis* (W83) at bacteria-to-monocyte ratios of 20:1, 50:1, or 100:1 for 2 h. Live bacterial exposure resulted in a reduction in intracellular IGFBP-6 protein levels, reaching statistical significance at the highest multiplicity of infection (*p* = 0.0190; Fig. 2A and B), consistent with the reduction observed in monocytes from individuals with periodontitis.

To model the inflammatory environment of periodontitis in a controlled and reproducible manner for subsequent mechanistic experiments, naïve monocytes and macrophages derived from healthy individuals were stimulated with LPS from *Porphyromonas gingivalis*, a key pathogen implicated in the disease^27^. Monocytes and macrophages were treated with 1 µg/mL or 0.1 µg/mL LPS, respectively, for 2 h in serum-free RPMI 1640 (supplemented with 40 ng/ml macrophage colony-stimulating factor (M-CSF) for macrophages), as serum was found to contain detectable levels of IGFBP-6^28^. LPS treatment significantly reduced IGFBP-6 protein levels in cell lysates of monocytes (*p* = 0.0469; Fig. 2C and D) and macrophages (*p* = 0.0312; Fig. 2E and F). By contrast, IGFBP-6 protein levels in conditioned media were significantly increased in both monocytes (*p* = 0.0022; Fig. 2G and H) and macrophages (*p* = 0.0135; Fig. 2I and J). In macrophage-conditioned media, IGFBP-6 appeared as a dimer. The functional significance of this form has not been defined in the literature and was not assessed in this study.

Egr-1 protein abundance was significantly elevated in both monocytes (*p* = 0.0421; Fig. 2K and L) and macrophages (*p* = 0.0070; Fig. 2M and N), consistent with the increased abundance of Egr-1 protein observed in cells from individuals with periodontitis.

### IGFBP-6 protein was located within extracellular vesicles in plasma and in vitro

Because IGFBP-6 protein was elevated in plasma from individuals with periodontitis, it was important to determine whether it circulated in a free, potentially bioactive form or was instead packaged within extracellular vesicles (EVs). This distinction is biologically important, as EV-associated proteins often have restricted receptor accessibility and reduced functional activity compared with freely soluble proteins.

**Fig. 3:**
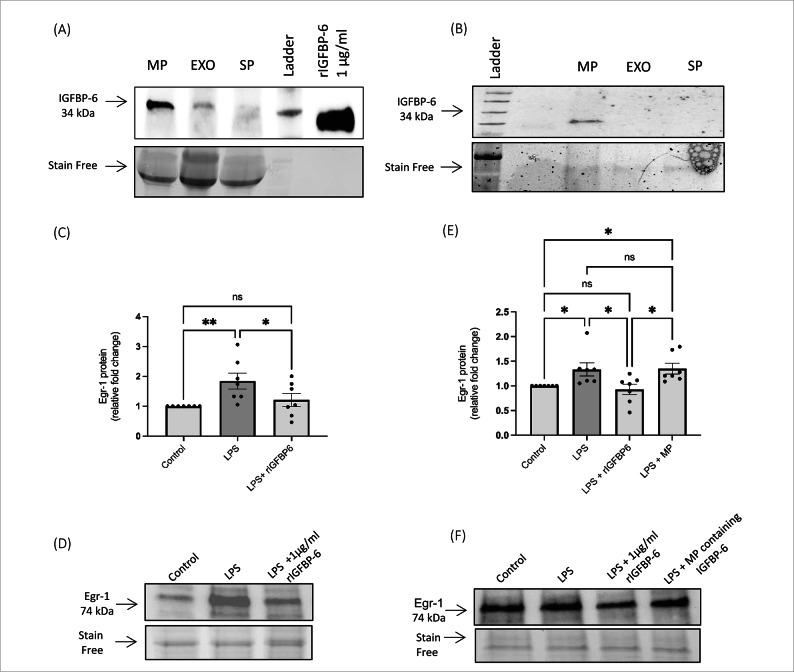
**IGFBP-6 is packaged within microparticles and attenuates Egr-1 expression in macrophages stimulated with** ***P. gingivalis*** **LPS.** **(A)** Plasma was collected from individuals with periodontitis (*n* = 6, mean age ± SD: 49.00 ± 7.97 years), pooled, and ultracentrifuged to isolate EVs. IGFBP-6 protein was detected in MP, EXO and SP fractions by Western blotting. **(B)** Dectection of IGFBP-6 protein by Western blotting in MP, EXO and SP fractions from the conditioned media of macrophages stimulated with 0.1 µg/mL *P. gingivalis* LPS for 2 h. **(C)** Quantification of Egr-1 protein in macrophages from healthy donors stimulated with LPS in the presence or absence of rIGFBP-6 (1 µg/mL) for 2 h (*p* = 0.0092, *n* = 7), with **(D)** representative Western blot. **(E)** Quantification of Erg-1 protein in macrophages treated with LPS in the presence or absence of either rIGFBP-6 or MPs containing IGFBP-6 (*p* = 0.0048, *n* = 7)), with **(F)** representative Western blot. Protein expression was quantified by densitometry and normalised using stain-free gel technology. Protein levels are expressed as fold change relative to untreated control. *ns*, not significant, *p < 0.05, **p < 0.01, using ANOVA for parametric repeated measures and Student-Newman-Keuls *post hoc* test. Error bars indicate SEM. Arrowheads indicate the detected protein bands; molecular weights are noted in kDa. Refer to Figures S12-S15 for full length Western blots and gels.

Plasma samples from six females with periodontitis (mean age ± SD: 49.00 ± 7.97 years) were pooled and subjected to differential ultracentrifugation. Western blotting showed IGFBP-6 enrichment in the larger EV or microparticle (MP) fraction, with lower levels in smaller EVs or exosomes (EXO) and minimal levels in the remaining EV-depleted soluble protein (SP) fraction. This suggests that IGFBP-6 in plasma is largely vesicle-associated rather than freely circulating (Fig. 3A).

To evaluate whether a similar mechanism occurs in vitro, LPS-treated macrophages from healthy donors were cultured in serum-free conditions, and conditioned media was collected and separated into MP, EXO and SP fractions by ultracentrifugation (refer to Figure S16 for EV characterisation). Western blotting revealed that IGFBP-6 protein was detectable only in MPs, confirming that vesicle-associated release also occurred in vitro under inflammatory conditions (Fig. 3B).

## Recombinant IGFBP-6 attenuated Egr-1 protein in macrophages, but not when delivered within extracellular vesicles

To investigate whether IGFBP-6 directly modulates inflammatory signalling in macrophages, cells were stimulated with *P. gingivalis* LPS in the presence or absence of recombinant IGFBP-6 (rIGFBP-6). After 2 h, Western blot analysis of cell lysates revealed that rIGFBP-6 significantly reduced Egr-1 protein levels compared to LPS alone (*p* = 0.0092; Fig. 3C and D), supporting an anti-inflammatory role for IGFBP-6 under these conditions.

To test whether this effect was preserved when IGFBP-6 was delivered within EVs, MP fractions containing IGFBP-6 protein were isolated from the conditioned media of LPS-treated macrophages and added to newly differentiated macrophages from the same donors. Cells were then re-stimulated with LPS, and Egr-1 protein levels were analysed. Comparable amounts of rIGFBP-6 protein were added to control cells but unlike rIGFBP-6, MPs containing IGFBP-6 did not reduce Egr-1 levels (*p* = 0.0048; Fig. 3E and F). This suggests that vesicle-associated IGFBP-6 protein is not biologically active, possibly due to limited access to the receptor.

## Recombinant IGFBP-6 did not alter monocyte adhesion but inhibited macrophage transmigration

**Fig. 4:**
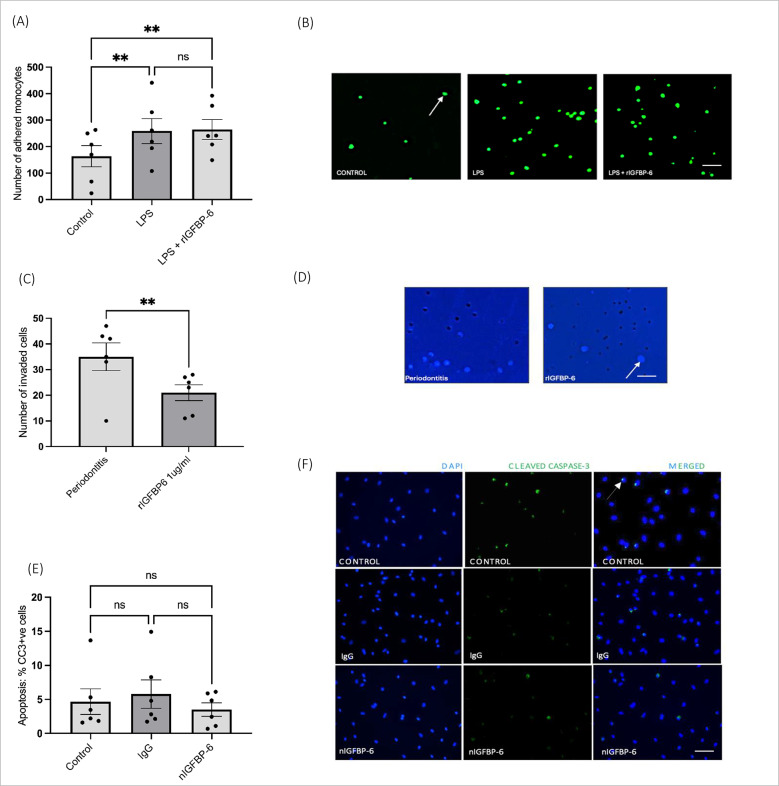
**Effects of IGFBP-6 on monocyte adhesion**,** macrophage transmigration**,** and foam cell apoptosis.** **(A)** To assess the role of IGFBP-6 in monocyte adhesion, naïve monocytes from healthy individuals were labelled with calcein-AM and incubated for 2 h with 0.1 µg/mL *P. gingivalis* LPS, with or without 1 µg/mL recombinant IGFBP-6 (rIGFBP-6). Monocytes were then seeded onto human coronary artery endothelial cells (HCAECs) for 30 min. Adherent monocytes were visualised using fluorescence microscopy and quantified (*p* = 0.0002, *n* = 6). **(B)** Representative fluorescence images; white arrow indicates an adhered monocyte. **(C)** To evaluate the effect of rIGFBP-6 on transmigration, monocyte-derived macrophages from individuals with periodontitis were cultured in a transwell chamber and treated with 1 µg/mL rIGFBP-6 or left untreated for 72 h. Transmigration was induced with 30 ng/mL CCL2, and nuclei of migrated cells on the underside of the membrane were stained with DAPI. Transmigration was quantified as the average number of cells that migrated through the transwell chamber pores per view. Ten views were analysed per condition (*p* = 0.0099, *n* = 6). **(D)** Representative images of migrated cells; white arrow indicates a transmigrated macrophage. **(E)** To investigate the role of IGFBP-6 in foam cell apoptosis, macrophages from healthy donors were exposed to 10 µg/mL oxLDL for 24 h in the presence of 5 µg/mL IgG or neutralising IGFBP-6 antibody (nIGFBP-6). Apoptotic cells were identified using immunocytochemistry for cleaved caspase-3 (CC3) (*p* = 0.2875, *n* = 6). Apoptosis was calculated by counting the number of CC3-positive cells and expressing as a percentage of the total number of cells, with ~ 50 cells counted per condition. **(F)** Representative images show CC3-positive cells (green) and DAPI-stained nuclei (blue); white arrow indicates apoptotic cell. Scale bar represents 50 μm and is applicable to all panels. *ns*, not significant, *p < 0.05, **p < 0.01, using one-way ANOVA with Student–Newman–Keuls *post hoc* test (A, E) or two-tailed, paired t-test (C). Error bars indicate SEM.

To assess whether IGFBP-6 modulates early monocyte-endothelial interactions relevant to atherogenesis, adhesion assays were performed using calcein-labelled naïve monocytes stimulated with LPS (0.1 µg/ml) ± rIGFBP-6. While LPS significantly increased monocyte adhesion to human coronary artery endothelial cells (HCAECs) (*p* = 0.0002; Fig. 4A and B), rIGFBP-6 had no additional effect, indicating that it does not regulate adhesion under these conditions.

In contrast, rIGFBP-6 significantly inhibited the transmigration of monocyte-derived macrophages from individuals with periodontitis across 8.0 μm Millicell inserts in response to 30 ng/ml CCL2 (*p* = 0.0099; Fig. 4C and D). These results suggest that IGFBP-6 is associated with macrophage transmigration and may limit macrophage recruitment to inflammatory sites.

## IGFBP-6 did not affect apoptosis in macrophage-derived foam cells

Given the role of foam cell apoptosis in promoting necrotic core formation and plaque instability^29,30^, we determined whether IGFBP-6 influenced macrophage apoptosis. Macrophages from healthy donors were treated with 10 µg/ml oxidised low-density lipoprotein (oxLDL) in the presence of 5 µg/ml neutralising IGFBP-6 antibody (nIGFBP-6; validated in Figure S17) or non-immune IgG control for 24 h. Apoptosis was assessed via immunocytochemistry for cleaved caspase-3 (CC3). No significant difference in CC3-positive cells was observed between nIGFBP-6-treated and control groups (*p* = 0.2875; Fig. 4E and F), suggesting that IGFBP-6 does not affect foam cell apoptosis under these experimental conditions.

### IGFBP-6 regulated foam cell formation in macrophages

**Fig. 5:**
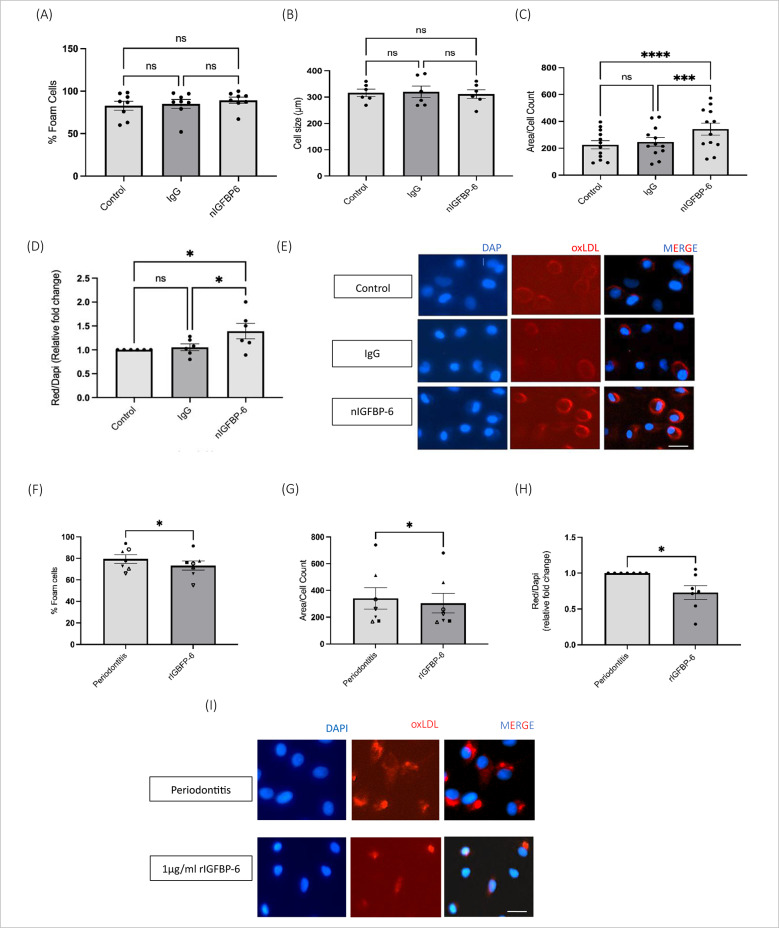
Modulation of macrophage-derived foam cell formation by IGFBP-6.**(A–E)** Macrophages from healthy individuals were treated with 10 µg/ml dil oxLDL for 24 h in the presence of either IgG or neutralising IGFBP-6 (nIGFBP-6) antibody and compared to untreated controls. **(A)** The percentage of foam cells was quantified by counting dil oxLDL-positive cells (red) and expressing this as a percentage of total nuclei stained with DAPI, with ~ 250 cells counted per condition (*p* = 0.0848, *n* = 8). **(B)** The size of foam cells was measured in µm² (*p* = 0.9897, *n* = 6). **(C)** Dil oxLDL area per foam cell was quantified in pixels and expressed as area divided by the total number of cells (*p* < 0.0001, *n* = 12). Ten views were analysed per condition. **(D)** The amount of intracellular oxLDL was quantified by measuring dil oxLDL fluorescence using a microplate reader (520 nm), normalised to cell number via DAPI fluorescence (365 nm) (*p* = 0.0202, *n* = 6). Representative images of macrophage-derived foam cells from **(E)** healthy individuals and **(I)** those with periodontitis showing nuclei stained with DAPI (blue) and oxLDL uptake (red). Scale bar represents 20 μm and is applicable to all panels. **(F-I)** Macrophages from individuals with periodontitis were treated with 1 µg/ml rIGFBP-6 protein in the presence of 10 µg/ml oxLDL for 24 h, and the number of foam cells and intracellular lipid content were compared to untreated control cells. **(F)** The number of foam cells formed was quantified by counting Dil-oxLDL (red)-positive cells and expressed as a percentage of the total number of cells (*p* = 0.0217, *n* = 7), with ~ 250 cells cells counted per condition. **(G)** Dil-oxLDL content in foam cells was quantified in pixels per cell (*p* = 0.0325, *n* = 7). **(H)** Total Dil-oxLDL fluorescence was measured using a microplate reader and normalised to cell number using DAPI fluorescence (*p* = 0.0325, *n* = 7). *p < 0.05, **p < 0.01, ***p < 0.001, ****p < 0.0001, using one-way ANOVA with Student-Newman-Keuls *post hoc* test or Friedman’s non-parametric test with Dunn’s *post hoc* test. Error bars indicate SEM.

Following transmigration into the subendothelial space, macrophages internalise retained lipoproteins and differentiate into foam cells; a hallmark of early atherosclerotic lesions^31^. To model the reduced IGFBP-6 protein levels observed in individuals with periodontitis, macrophages derived from healthy donor monocytes were treated with nIGFBP-6 and cultured with 10 µg/ml Dil-labelled oxLDL for 24 h. Compared to untreated and IgG controls, nIGFBP-6 treatment significantly increased intracellular oxLDL accumulation (*p* < 0.0001), although foam cell number and size remained unchanged (Fig. 5A-C). This effect was confirmed by quantitative fluorescence analysis (*p* = 0.0202), suggesting that reduced IGFBP-6 availability enhances lipid uptake in macrophages (Fig. 5D and E).

To assess whether restoring IGFBP-6 protein levels could attenuate foam cell formation in macrophages derived from individuals with periodontitis, where IGFBP-6 protein abundance was reduced, macrophages from those with periodontitis were treated with 1 µg/ml rIGFBP-6 in the presence of oxLDL. Recombinant IGFBP-6 significantly reduced both foam cell number (*p* = 0.0217, Fig. 5F) and intracellular lipid content (*p* = 0.0312; Fig. 5G, I). These findings were corroborated by microplate-based fluorescence quantification (*p* = 0.0325, Fig. 5H), confirming reduced oxLDL retention. These findings suggest that restoring IGFBP-6 levels can counteract the enhanced foam cell formation observed in individuals with periodontitis, consistent with a protective role for IGFBP-6 in macrophage lipid metabolism.

### IGFBP-6 does not alter scavenger receptor expression but is associated with changes in ABCG1 abundance and cholesterol efflux

To explore the mechanism underlying the enhanced lipid accumulation observed upon IGFBP-6 neutralisation, the expression of three key scavenger receptors involved in oxLDL uptake - CD36, LOX-1, SR-A1 - were examined. Macrophages from healthy donors were treated with 5 µg/ml nIGFBP-6 or IgG control and exposed to 10 µg/ml oxLDL. After 6 h (for mRNA) or 24 h (for protein), levels were measured by quantitative PCR (qPCR) or Western blotting, respectively. Neutralisation of IGFBP-6 did not significantly alter the amount of *CD36* (*p* = 0.3673), *LOX-1* (*p* = 0.1821), or *SR-A1* (*p* = 0.3673) mRNAs relative to untreated or IgG controls (Figure S17 A–C). Similarly, the abundance of CD36 (*p* = 0.6914), LOX-1 (*p* = 0.1821), and SR-A1 (*p* = 0.5216) proteins remained unchanged (Figure S18 D–I). These findings suggest that the observed increase in lipid accumulation is not mediated through upregulation of classical scavenger receptors.

**Fig. 6:**
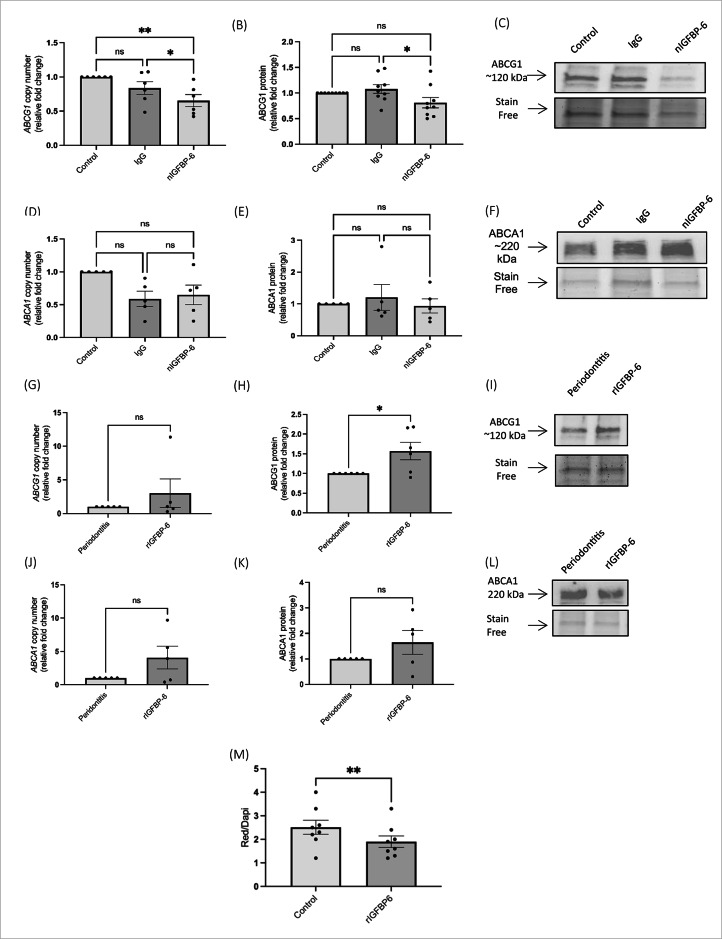
The effects of IGFBP-6 on reverse cholesterol transporter expression in macrophage-derived foam cells.**(A–F)** Macrophages from healthy individuals were treated with 10 µg/ml oxLDL in the presence of either 5 µg/ml IgG or 5 µg/ml nIGFBP-6 antibody for 6 h (for mRNA) or 24 h (for protein). Quantification of ABCG1 **(A)** mRNA expression (*p* = 0.0050, *n* = 6) or **(B**,** C)** protein expression (*p* = 0.0217, *n* = 9) in nIGFBP-6-treated cells compared to controls. Quantification of ABCA1 **(D)** mRNA expression (*p* = 0.6880, *n* = 5) or **(E**,** F)** protein expression (*p* = 0.6914, *n* = 5) in nIGFBP-6-treated cells compared to controls. **(G-L)** Macrophages from individuals with periodontitis were treated with 1 µg/ml rIGFBP-6 in the presence of 10 µg/ml oxLDL for 6 h (mRNA) or 24 h (protein). Quantification of ABCG1 **(G)** mRNA expression (*p* = 0.8125, *n* = 5) or **(H**,** I)** protein expression (*p* = 0.0358, *n* = 6) in rIGFBP-6-treated cells compared to controls. Quantification of ABCA1 **(J)** mRNA expression (*p* = 0.3125, *n* = 5) or **(K**,** L)** protein expression (*p* = 0.3125, *n* = 5) in rIGFBP-6-treated cells compared to controls. mRNA values are presented as relative fold change in copy number per ng of extracted RNA, calculated using a standard curve. Protein expression was quantified by densitometry, normalised to total protein using stain-free gel technology and expressed as fold change relative to control **(M)** Macrophages from individuals with periodontitis were cultured with 10 µg/ml dil oxLDL for 24 h to form foam cells, washed with PBS, and treated with 1 µg/ml rIGFBP-6 for an additional 24 h. The amount of intracellular oxLDL was quantified by measuring dil oxLDL fluorescence using a microplate reader (520 nm), normalised to cell number via DAPI fluorescence (365 nm) (*p* = 0.0071, *n* = 8). *ns*, not significant, *p < 0.05, **p < 0.01,using ANOVA for parametric repeated measures and Student–Newman–Keuls *post hoc* test or two-tailed, paired t-tests. Data are expressed in fold change relative to control. Error bars indicate SEM. Arrowheads indicate the detected band; molecular weights are indicated in kDa. Refer to Figures S19-S22 for full length Western blots and gels.

Given the absence of changes in scavenger receptors, the abundance of proteins involved in cholesterol efflux was also examined. Neutralising IGFBP-6 significantly reduced ATP-binding cassette subfamily G member 1 (*ABCG1*) mRNA (*p* = 0.0050; Fig. 6A) and the abundance of ABCG1 protein (*p* = 0.0217; Fig. 6B and C), while ATP-binding cassette subfamily A member 1 (*ABCA1*) mRNA (*p* = 0.6880; Fig. 6D) and protein (*p* = 0.6914; Fig. 6E and F) levels were unchanged. These data suggest that reduced IGFBP-6 availability is associated with impaired cholesterol efflux and decreased ABCG1 abundance, and consequently may contribute to foam cell formation in the early stages of atherosclerosis.

### Recombinant IGFBP-6 is associated with increased ABCG1 protein abundance and enhanced lipid efflux in established foam cells from individuals with periodontitis

To determine whether IGFBP-6 protein could restore impaired efflux mechanisms in disease, macrophages derived from individuals with periodontitis were treated with 1 µg/ml rIGFBP-6 in the presence of oxLDL. After 6 and 24 h, mRNA and protein levels, respectively, of ABCG1 and ABCA1 were assessed. Treatment with rIGFBP-6 did not alter the abundance of *ABCG1* mRNA (*p* = 0.8125; Fig. 6G) but was associated with a significant increase in ABCG1 protein levels (*p* = 0.0358; Fig. 6H and I), suggesting post-transcriptional regulation. *ABCA1* mRNA (*p* = 0.3125; Fig. 6J) and protein (*p* = 0.3125; Fig. 6K and L) were unaffected. These findings suggest a potential link between IGFBP-6 availability and ABCG1 protein abundance that may contribute to its protective effects on macrophage lipid handling.

To assess whether IGFBP-6 influences lipid efflux from already-formed foam cells, macrophages from individuals with periodontitis were preloaded with 10 µg/ml Dil oxLDL for 24 h. After washing to remove extracellular oxLDL, cells were incubated with or without 1 µg/ml rIGFBP-6 for a further 24 h. Fluorescence quantification revealed a significant reduction in intracellular oxLDL in rIGFBP-6-treated cells compared to controls (*p* = 0.0071; Fig. 6M). These findings suggest that rIGFBP-6 is associated with enhanced cholesterol removal from established foam cells, consistent with a role in promoting reverse cholesterol transport.

## IGFBP-6 effects were independent of IGF-2 inhibition

The primary function of IGFBP-6 is to prolong the half-life of IGF-2 in the circulation^15^. Therefore, it was important to compare the amount of IGF-2 protein in naïve monocytes and plasma from individuals with periodontitis relative to healthy controls. IGF-2 protein levels were comparable between groups in both monocytes (*p* = 0.6825) and plasma samples (*p* = 0.1404; (Figure S22), indicating that IGF-2 is not dysregulated in periodontitis.

To further assess whether IGFBP-6 affects foam cell formation through IGF-2 signalling, macrophages from individuals with periodontitis were treated with 5 µg/ml neutralising IGF-2 antibody (nIGF-2; validated in Figure S24) or IgG control in the presence of 10 µg/ml Dil-oxLDL for 24 h. Foam cell formation was assessed by fluorescence microscopy. Neutralisation of IGF-2 did not significantly alter the proportion of foam cells formed (*p* = 0.2505) or the amount of intracellular oxLDL (*p* = 0.9306; Figure S24). These findings suggest that the effect of IGFBP-6 on foam cell formation is independent of IGF-2 activity.

## Recombinant IGFBP-6 attenuated CXCL10 and IL-18 production in LPS-stimulated macrophages

**Fig. 7:**
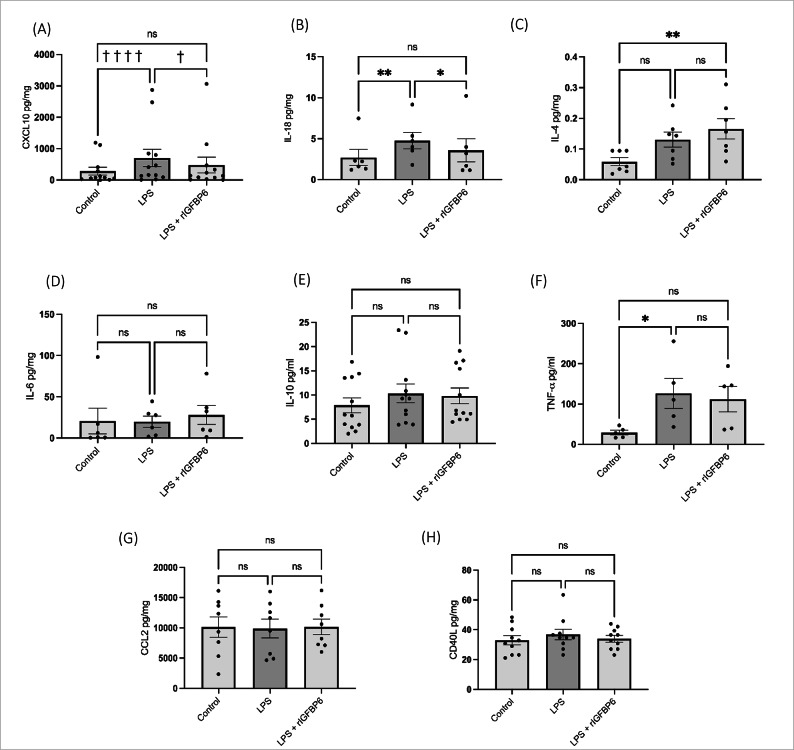
The effect of rIGFBP-6 protein on inflammatory cytokines from macrophages stimulated with LPS. Macrophages derived from PBMCs from healthy individuals were treated on day 7 of differentiation with 0.1 µg/ml *P. gingivalis* LPS, with or without 1 µg/ml rIGFBP-6, in serum-free RPMI supplemented with 40 ng/ml M-CSF for 2 h. Conditioned media was collected and concentrated for cytokine analysis. A Milliplex human cytokine kit was used to quantify the following inflammatory cytokines: CXCL10 (**A**; *p* < 0.0001, *n* = 12), IL-18 (**B**; *p* = 0.0057, *n* = 6), IL-4 (**C**; *p* = 0.0085, *n* = 7), IL-6 (**D**; *p* = 0.4861, *n* = 6), IL-10 (**E**; *p* = 0.0755, *n* = 12), TNF-α (**F**; *p* = 0.0394, *n* = 5), CCL2 (**G**; *p* = 0.9597, *n* = 8), and sCD40L (**H**; *p* = 0.0402, *n* = 10). IL-1β, IL-3, and IL-13 were undetectable in all conditions. Data are presented as mean cytokine concentration in pg/ml ± SEM. Statistical analysis was performed using one-way repeated measures ANOVA with Student–Newman–Keuls *post hoc* test for parametric data (*ns*, not significant, *p < 0.05, **p < 0.01) or Friedman’s test with Dunn’s *post hoc* test for non-parametric data (†*p* < 0.05, ††††*p* < 0.0001).

To investigate the immunomodulatory role of IGFBP-6 in macrophage-driven inflammation, cells were stimulated with *P. gingivalis* LPS (0.1 µg/ml) in the presence or absence of 1 µg/ml rIGFBP-6. Following 2 h of stimulation, conditioned media was collected, concentrated, and analysed using a Milliplex human cytokine panel. Recombinant IGFBP-6 significantly reduced the secretion of C-X-C motif chemokine ligand 10 (CXCL10) (*p* < 0.0001) and interleukin (IL) 18 (IL-18) (*p* = 0.0057; Fig. 7A and B). Other measured cytokines including IL-4 (*p* = 0.0085), IL-6 (*p* = 0.4861), IL-10 (*p* = 0.0755), tumour necrosis factor-α (TNF-α) (*p* = 0.0394), CCL2 (*p* = 0.9597), and soluble CD40 ligand (sCD40L) (*p* = 0.0402) showed no consistent modulation by rIGFBP-6 (Fig. 7). IL-1β, IL-3, and IL-13 were undetectable. These data support a selective anti-inflammatory effect of IGFBP-6 in macrophages, particularly on CXCL10 and IL-18, which are inflammatory mediators relevant to the pathogenesis of atherosclerosis.

## Discussion

### Proteomics and pathway analysis reveal metabolic and immune dysregulation with reduced IGFBP-6 protein in monocytes from individuals with periodontitis

Canonical pathway analysis of differentially expressed proteins in naïve monocytes from people with periodontitis identified marked changes in metabolic, inflammatory, and immune-regulatory pathways. Downregulation of oxidative phosphorylation and fatty acid.

β-oxidation, alongside predicted mitochondrial dysfunction, suggests metabolic reprogramming towards a pro-inflammatory state. This aligns with evidence that infections drive metabolic shifts in monocytes and macrophages, favouring glycolysis and inflammatory activation^32–34^.

Bioinformatic analysis identified IGFBP-6 as a protein of particular interest. IGFBP-6 abundance was significantly reduced in naïve monocytes from individuals with periodontitis compared with controls. Importantly, reduced IGFBP-6 expression persisted as monocytes differentiated into macrophages, suggesting a chronic and sustained effect that may influence immune cell function even after recruitment to atherosclerotic lesions. These results were replicated in vitro by the addition of *P. gingivalis* LPS to both naïve monocytes and macrophages, suggesting that IGFBP-6 is released from these cells in response to the dysbiotic microbiota associated with periodontitis. The clinical relevance of IGFBP-6 in the pathogenesis of atherosclerosis is supported by studies showing its reduced expression in unstable carotid plaques relative to stable lesions and normal arteries^20,24,25^. Notably, IGFBP-6 has been shown to localise with CD68⁺ macrophages in the vulnerable region between the fibrous cap and necrotic core, suggesting that local IGFBP-6 loss may affect plaque stability^20^. Supporting this, global deletion of IGFBP-6 in ApoE^⁻/⁻^ mice exacerbates atherosclerotic plaque burden and vascular inflammation, while endothelial-specific IGFBP-6 overexpression attenuates lesion development^19^. While this previous study focused on endothelial mechanisms, our findings extend the functional significance of IGFBP-6 to monocytes and macrophages, suggesting that reduced expression exacerbates atherosclerosis through distinct but complementary effects across vascular and immune cell types. Restoring IGFBP-6 protein in monocytes/macrophages may therefore represent a novel strategy to limit inflammation and atheroprogression, particularly in individuals with chronic inflammatory diseases such as periodontitis.

#### IGFBP-6 is associated with macrophage behaviours relevant to atherogenesis

Monocyte recruitment, foam cell formation, and macrophage-driven inflammation are pivotal events in the development and progression of atherosclerotic plaques^2,4,31,35^. Our findings indicate that IGFBP-6 is associated with each of these stages and exerts anti-atherosclerotic effects. Recombinant IGFBP-6 protein significantly inhibited transmigration of monocyte-derived macrophages from individuals with periodontitis, suggesting a potential role in limiting macrophage infiltration into sites of inflammation. Furthermore, macrophages with reduced IGFBP-6 levels, either through downregulation in periodontitis or experimental neutralisation, exhibited augmented foam cell formation. Neutralising IGFBP-6 increased intracellular lipid accumulation, whereas recombinant IGFBP-6 reduced foam cell formation and lipid retention in macrophages from individuals with periodontitis.

These observed effects on foam cell formation were not associated with changes in classical scavenger receptors (CD36, SR-A1, LOX-1), but instead correlated with reduced cholesterol efflux capacity and decreased abundance of the cholesterol transporter ABCG1. Importantly, recombinant IGFBP-6 restored ABCG1 protein levels and promoted lipid efflux even in pre-formed foam cells, supporting a functional association with cholesterol clearance.

Notably, the reduction in cholesterol efflux capacity and ABCG1 abundance under conditions of limited IGFBP-6 were accompanied by increased abundance of the transcription factor Egr-1 in patient-derived monocytes. Egr-1 is recognised as a regulator of inflammatory and metabolic gene networks and has been implicated in coordinating immune activation and lipid handling in myeloid cells^36,37^.The observed changes in Egr-1 and ABCG1 abundance suggest a broader association between inflammatory signalling and cholesterol efflux capacity, rather than a single defined molecular pathway.

In parallel, IGFBP-6 selectively attenuated macrophage-derived inflammatory responses. In LPS-stimulated primary human macrophages, rIGFBP-6 significantly reduced secretion of the pro-inflammatory chemokine CXCL10 and cytokine IL-18, both implicated in plaque progression and destabilisation^38–40^.This reduction was accompanied by decreased abundance of the transcription factor Egr-1, a known regulator of cytokine production via NF-κB^41,42^, suggesting that modulation of Egr-1 could contribute to altered inflammatory signalling. However, the lack of effect of IGFBP-6 on other canonical NF-κB regulated mediators, including TNF-α and CCL2, indicates that this pathway is unlikely to fully account for the observed effects. While the availability of IGFBP-6 is clearly associated with selective changes in macrophage inflammatory output, the precise molecular mechanism remains to be determined and will require more detailed investigation beyond the scope of the present study. These results align with broader evidence for an anti-inflammatory role of IGFBP-6, where recombinant IGFBP-6 reduced IL-1β, IL-6, and TNF-α expression in epithelial cells, while IGFBP-6 neutralisation enhanced cytokine production^43^. In vivo, decreased IGFBP-6 expression has been observed in inflamed tissues obtained from asthmatic lung, while its restoration was associated with resolution of inflammation^44^. However, context-dependent effects have also been reported, with pro-inflammatory activity observed in non-myeloid or transformed cells^18,45,46^, indicating that IGFBP-6 function may vary by cell type and stimulus.

Together, these findings position IGFBP-6 as a novel factor associated with macrophage behaviour that constrains transmigration, foam cell formation and inflammatory signalling, thereby supporting a potential protective role in modulating plaque development and instability.

#### IGFBP-6 is secreted within EVs and is not biologically active

In this study, plasma IGFBP-6 protein levels were found to be increased in individuals with periodontitis; an observation that might initially be expected to be protective. However, the epidemiological evidence shows that people with periodontitis have higher rates of atherosclerosis^8–10^. To address this paradox, the mechanism of IGFBP-6 release from macrophages was examined; specifically, whether IGFBP-6 was secreted in EVs, as this might explain the apparent disconnect between elevated circulating protein and persistent vascular risk. Plasma IGFBP-6 from individuals with periodontitis was primarily found to be packaged within EVs. Moreover, while the anti-inflammatory effects of IGFBP-6 were evident when applied as recombinant protein, EV-associated IGFBP-6 derived from LPS-stimulated macrophages and plasma lacked this activity. Specifically, EV-bound IGFBP-6 did not reduce Egr-1 expression in LPS-treated macrophages, unlike free rIGFBP-6. This suggests that packaging within EVs is associated with reduced IGFBP-6 bioactivity, potentially by limiting receptor access or altering its structure. Consistent with this, IGFBP-6 has been shown to be secreted in either free or vesicle-bound forms depending on the cell type and the nature of the stimulus. For example, monocyte-derived dendritic cells secrete IGFBP-6 in EVs under hyperthermic stress, but as free protein under oxidative stress^45^. In the context of periodontitis, elevated plasma levels of IGFBP-6 may therefore not confer an anti-inflammatory effect as the protein is located within EVs. Further research is needed to clarify the biological fate and intercellular signalling potential of EV-associated IGFBP-6, particularly its relevance in modulating macrophage behaviour and vascular inflammation.

#### Limitations

This study used a discovery-based approach to examine IGFBP-6-associated changes in monocyte and macrophage function using in vitro and ex vivo models. As periodontitis is a polymicrobial infection, future studies should explore the effects of additional bacterial species, their virulence factors (including gingipains, outer membrane vesicles and microbial metabolites), and mixed bacterial communities to better reflect the complexity of the in vivo environment. Future work incorporating detailed clinical phenotyping, monocyte-endothelial interactions and in vivo models will help define the broader biological and clinical relevance of IGFBP-6. Additional studies examining downstream signalling pathways, including targeted modulation of candidate transcriptional regulators such as Egr-1 and surface expression of lipid-handling receptors may also provide additional insight into the mechanisms linking inflammation and cholesterol efflux.

## Conclusions

Proteomic analysis of naïve monocytes from individuals with periodontitis revealed widespread changes in immune and metabolic pathways, supporting a shift toward a pro-inflammatory and dysfunctional phenotype. Among the differentially expressed proteins, IGFBP-6 emerged as a key candidate, with consistently reduced abundance in monocytes and macrophages. Functional experiments demonstrated that IGFBP-6 deficiency promotes cholesterol accumulation, pro-inflammatory cytokine release, and macrophage transmigration, which are all processes central to atherogenesis. Notably, IGFBP-6 protein was associated with changes in foam cell formation and ABCG1 abundance, an effect that was independent of IGF-2 in this experimental context. While recombinant IGFBP-6 restored several protective macrophage functions, EV-associated IGFBP-6 lacked bioactivity. These findings suggest that reduced IGFBP-6 protein within monocytes and macrophages contributes to the altered immune response in individuals with periodontitis, and may exacerbate cardiovascular risk through its effects on monocyte/macrophage behaviour.

### Methods

#### Human monocyte and plasma isolation from peripheral blood

Peripheral blood (10–20 mL) was collected via venepuncture from individuals with severe periodontitis or healthy controls under approved ethical protocols (REC: 10/H0107/32, 15/WA/0209, 21/PR/1095). The demographics and characteristics of donors are provided in Tables S1-3. The protocol for collecting peripheral blood from these individuals is outlined in a study registered with ISRCTN (Reference: ISRCTN10537092). Blood was diluted 1:1 with sterile Dulbecco’s phosphate-buffered saline (DPBS) without calcium or magnesium and layered over 15 mL of Ficoll-Paque Plus (Merck) in SepMate-50 tubes (Stemcell Technologies). Density gradient centrifugation was performed at 1,200 *g* for 10 min at room temperature. Following centrifugation, 1 mL of plasma was collected and stored at − 80 °C. The peripheral blood mononuclear cell (PBMC) layer was poured into a sterile tube, centrifuged at 400 *g* for 10 min, and washed twice with Hank’s Balanced Salt Solution (HBSS).

Monocytes were isolated by adherence, as described previously^47^. Briefly, PBMCs were resuspended in RPMI-1640 (ThermoFisher Scientific) supplemented with 10% foetal bovine serum (FBS), 2 mM L-glutamine, 100 IU/ml penicillin and 100 µg/ml streptomycin, and then 500–1,000 µL of cell suspension was plated in 24- or 12-well plates. After 2 h incubation at 37 °C with 5% CO₂, non-adherent cells were removed.

To generate monocyte-derived macrophages, monocytes were cultured for 7 days in RPMI-1640 containing 10% FBS and 20 ng/mL monocyte colony stimulating factor (M-CSF; Miltenyi Biotec), with media changes every 2 days.

For functional assays, recombinant IGFBP-6 (R&D Systems; 876-B6), neutralising IGFBP-6 antibody (R&D Systems; MAB8762) or isotype IgG control (R&D Systems; MAB004) were used, where indicated. In separate experiments, recombinant IGF-2 (R&D Systems; 292-G2), neutralising IGF-2 antibody (R&D Systems; MAB292) or IgG control (R&D Systems; MAB002) were applied to assess whether IGFBP-6 effects were mediated via IGF-2 signalling.

To assess the effect of whole bacterial exposure, naïve monocytes were stimulated with live *Porphyromonas gingivalis* W83 (ATCC; BAA-308), cultured as previously described^48^, at bacteria-to-monocyte ratios of 20:1, 50:1 or 100:1 for 2 h under serum-free conditions. To model the inflammatory environment of periodontitis, cells were stimulated with ultrapure *P. gingivalis* LPS (InvivoGen) at 0.1 µg/mL for macrophages and 1 µg/mL for monocytes, providing a defined TLR4-biased inflammatory stimulus. These concentrations were selected based on preliminary dose-response optimisation to identify the lowest doses that produced consistent modulation of IGFBP-6 and downstream functional readouts and most closely reflected patterns observed in patient-derived cells.

### Human coronary artery endothelial cell culture

Frozen HCAECs (Promocell) were thawed in a 37 °C water bath and seeded into T150 flasks containing endothelial cell growth medium MV (Promocell) supplemented with endothelial growth supplement mix, penicillin (100 IU/mL), and streptomycin (100 µg/mL). The demographics and characteristics of donors are shown in Table S4. Cultures were maintained at 37 °C and 5% CO₂. Media were replaced after 24 h to remove non-viable cells and refreshed every 2–3 days thereafter. Upon reaching confluence, cells were washed with DPBS and detached using 5 mL of 0.05% trypsin-EDTA (Gibco) for 5 min at 37 °C. Trypsin activity was quenched with an equal volume of endothelial growth medium. Detached cells were confirmed by microscopy and used for experiments at passages 4–5.

### Proteomic analysis of monocytes

The sample size for the proteomic discovery analysis (*n* = 15 per group) was selected in line with prior quantitative proteomic studies of primary human monocytes and immune cell populations, where cohort sizes in the range of 10–15 donors have been shown to provide robust and reproducible detection of biologically meaningful differences in protein abundance^49,50^. Naïve monocytes were isolated from PBMCs. Adherent monocytes were lysed in RIPA buffer (ThermoFisher) supplemented with protease inhibitor cocktail (Sigma-Aldrich) and universal nuclease (ThermoFisher). Proteomic analysis was performed at the University of Bristol Proteomics Facility. Proteins (25 µg per sample) were digested with trypsin, labelled using 10-plex Tandem Mass Tag (TMT) reagents (ThermoFisher), and pooled. Peptides were desalted using SepPak cartridges, fractionated by high-pH reversed-phase chromatography (Ultimate 3000, Thermo Scientific), and analysed by nano-LC-MS/MS using an Orbitrap Fusion Lumos mass spectrometer. Chromatographic separation used C18 columns and a 150-minute gradient. MS data were acquired in SPS-MS3 mode with defined instrument settings, including Orbitrap FTMS1 resolution (120,000), ITMS2 CID energy (35%), FTMS3 HCD energy (60%), and AGC targets and injection times per MS level.

Raw data files were processed with Proteome Discoverer v2.1 (Thermo Scientific), and peptide identification was performed against the UniProt Human database (September 2018 release, 152,927 entries) using the SEQUEST algorithm. Peptide precursor mass tolerance was set at 10 ppm, and MS/MS tolerance was set at 0.6 Da. Search criteria included oxidation of methionine (+ 15.9949) as a variable modification and carbamidomethylation of cysteine (+ 57.0214) and the addition of the TMT mass tag (+ 229.163) to peptide N-termini and lysine as fixed modifications. Searches were performed with full tryptic digestion and a maximum of 2 missed cleavages were allowed. The reverse database search option was enabled, and all data were filtered to satisfy false discovery rate (FDR) of 5%.

Protein abundance values from pooled donor samples were log₂-transformed. Differential expression between periodontitis and control monocyte proteomes was calculated using unpaired t-tests, and proteins with p-values < 0.05 were considered statistically significant. A log₂ fold change (logFC) > 0 indicated upregulation in periodontitis samples, while a negative logFC indicated downregulation. Pathway analysis was performed using IPA (Qiagen Bioinformatics). Proteins with *p* < 0.01 and logFC > 1.3 were included. IPA was used to identify canonical pathways, upstream regulators, and predicted functional networks. Significance thresholds included an absolute z-score ≥ 2 for activation or inhibition predictions.

### mRNA expression analysis by quantitative PCR

Total RNA was extracted using the miRNeasy Mini Kit (Qiagen) following cell lysis in QIAzol reagent. Phase separation was performed with chloroform, and RNA was purified using spin columns with ethanol and proprietary wash buffers. RNA concentration and purity were assessed using a Nanodrop spectrophotometer, and samples with 260/280 ratios < 1.8 or 260/230 ratios > 2.2 were excluded. Complementary DNA (cDNA) was synthesized from 100 ng of RNA using the High-Capacity cDNA Reverse Transcription Kit (Applied Biosystems). Quantitative PCR was performed using the LightCycler 480 System (Roche) with SYBR Green I Master Mix. Each reaction contained 5 µl SYBR Green mix, 3 µl water, 1 µl primers, and 1 µl cDNA. Reactions were run in triplicate alongside no-template and no-RT controls. Thermocycling conditions included an initial denaturation step, 45 amplification cycles, and melting curve analysis to confirm product specificity.

Relative mRNA expression was determined using the cycle threshold (Ct) method and only replicates within 1 Ct value were included. Absolute quantification was performed using a standard curve generated from serial dilutions of pooled qPCR product. Amplicon copy number was calculated based on molecular weight and fragment length. Details of the primers used can be found in Table S5.

### Western blotting

Cells were lysed using sodium dodecyl sulfate (SDS) lysis buffer (50 mM Tris-HCl, pH 6.8, 5% (w/v) SDS, 10% (v/v) glycerol) and stored at − 80 °C until analysis. Conditioned media was concentrated 30-fold using Amicon Ultra-0.5 centrifugal filter units (10 kDa cutoff; Millipore). Protein concentrations were quantified using a microBCA protein assay kit (ThermoFisher Scientific) against a bovine serum albumin (BSA) standard curve, following manufacturer’s instructions.

For SDS–polyacrylamide gel electrophoresis (SDS-PAGE), protein lysates were diluted to equal protein concentrations, mixed with Laemmli sample buffer, and heated at 95 °C for 5 min. Samples were separated on 4–15% Mini-PROTEAN TGX stain-free gels (Bio-Rad) and visualized under UV light using a ChemiDoc MP system. Proteins were transferred to nitrocellulose membranes using the Trans-Blot Turbo system (Bio-Rad). Membranes were blocked in 5% (w/v) BSA/TBS-T and incubated overnight at 4 °C with primary antibodies, followed by horseradish peroxidase (HRP)-conjugated secondary antibodies. Detection was performed using Luminata Forte substrate (Merck), and images were captured with a ChemiDoc MP system. Band intensity was quantified using Image Lab software (Bio-Rad). Densitometry values were normalized to total protein detected via stain-free imaging technology (BioRad™) or using β-actin as a loading control. Molecular weights were determined using a protein ladder (Geneflow).

### Visualisation of intracellular lipids

Foam cell formation was assessed in macrophages by uptake of fluorescently labelled oxidized low-density lipoprotein (Dil oxLDL; ThermoFisher Scientific). Validation of the neutralising IGFBP-6 and IGF-2 antibodies was performed (Figure S17 and S24). Macrophages were cultured on sterile glass coverslips placed in 24-well plates. On day 7 of differentiation, macrophages cultured in 24-well plates were incubated with Dil oxLDL at a concentration of 10 µg/mL in RPMI-1640 supplemented with 10% (v/v) FBS, 2 mM L-glutamine, 100 IU/ml penicillin and 100 µg/ml streptomycin for 24 h at 37 °C in 5% CO₂. Non-adherent cells were removed by washing three times with PBS. Cells were fixed in 3% (w/v) paraformaldehyde for 15 min and mounted with ProLong™ Gold Antifade Mountant containing 4′,6-diamidino-2-phenylindole (DAPI; ThermoFisher Scientific). Slides were covered with glass coverslips, and fluorescence imaging was performed using the EVOS FL Color Imaging System (20× magnification). Ten fields per well were imaged (~ 250 cells per view), and lipid uptake (red fluorescence) and nuclei (blue) were quantified. Foam cell number was expressed as a percentage of total cells. Foam cell size was quantified as cell area (µm²) by manual delineation of DiI-oxLDL–positive cells in Fiji (ImageJ) on merged brightfield and fluorescence images, following calibration using the embedded scale bar (see Figure S26). DiI-oxLDL intensity was quantified as red fluorescent signal per cell.

In parallel, fluorescence intensity was quantified in macrophages cultured in black 96-well clear-bottom plates (Greiner Bio-one). Following 24 h exposure to Dil oxLDL (10 µg/mL), wells were washed and fluorescence was measured at 520 nm using a GloMax^®^ Explorer Microplate Reader (Promega). Cells were then permeabilized with cold methanol (150 µL, 10 min), washed, and stained with DAPI for quantification of nuclei. DAPI fluorescence was measured at 365 nm. Final Dil oxLDL uptake was calculated by subtracting background fluorescence and normalizing to DAPI fluorescence.

#### Macrophage transmigration assay

On day 7, macrophages cultured from individuals with periodontitis were detached using 200 µL Accutase (ThermoFisher Scientific) for 10 min at 37 °C, followed by gentle scraping. Detached cells were centrifuged at 300 *g* for 10 min, and the pellet was resuspended in 500 µL of pre-warmed RPMI-1640 culture medium. Millicell hanging inserts (8.0 μm pore size; Millipore) were rehydrated in 24-well plates by adding 500 µL of warm, serum-free RPMI medium and incubating at 37 °C in 5% CO₂ for 2 h. After rehydration, the medium was removed. The lower chamber was filled with 750 µL RPMI medium supplemented with 30 ng/mL chemokine ligand 2 (CCL2; PeproTech), with or without 1 µg rIGFBP-6. In the upper chamber, 2.5 × 10⁴ macrophages in 500 µL serum-free RPMI medium containing 40 ng/mL M-CSF, with or without rIGFBP-6, were added to the inserts. After a 72-h incubation at 37 °C, non-migrated cells were removed by gently swabbing the upper membrane surface with a PBS-soaked cotton bud. Chambers were washed twice with PBS. Cells were fixed with ice-cold methanol and washed again. Inserts were mounted bottom-side down on slides using ProLong™ Gold Antifade Mountant with DAPI and glass coverslips. Migrated cells were imaged using an Olympus BX41 microscope and Q-Capture Pro software. Cell migration was quantified by counting DAPI-stained nuclei in 10 randomly selected fields per insert (10–20 cells per view), and expressed as the average number of migrated cells per field.

### Extracellular vesicle isolation

Macrophages were stimulated with 0.1 µg/mL ultra-pure *P. gingivalis* LPS in serum-free RPMI medium containing 40 ng/mL M-CSF for 2 h at 37 °C. Conditioned media were collected and centrifuged at 1,000 *g* for 10 min at room temperature to remove cellular debris. EVs were isolated by differential ultracentrifugation based on a modified protocol^51^, while maintaining sterility for EVs used in functional assays. Conditioned media were diluted with PBS to a final volume of 35 mL and transferred into ultracentrifuge tubes (Beckman). Samples were centrifuged at 20,000 *g* for 30 min at 4 °C to isolate microparticles (MP fraction). Pellets were either lysed in 1% (w/v) SDS for Western blotting or resuspended in PBS for functional assays. The supernatant was transferred to fresh tubes and centrifuged at 100,000 *g* for 2 h at 4 °C to isolate exosomes. Resulting pellets (EXO fraction) and final supernatants (soluble proteins) were stored at − 80 °C or used immediately in downstream applications. EVs were characterized by Western blotting for established protein markers to confirm the purity of the isolation (Figure S16).

#### Multiplex cytokine assay

Cytokine and chemokine secretion from macrophages was assessed using the MILLIPLEX^®^ MAP Human Cytokine/Chemokine Magnetic Bead Panel (Millipore). Reagents and conditioned media samples were brought to room temperature before use. A 96-well magnetic plate was washed with 200 µL of wash buffer and shaken at 650 rpm for 10 min. Wells were prepared with 25 µL assay buffer and 25 µL of serum-free RPMI for standards, controls, and blanks. Samples were loaded with 10 µL of concentrated conditioned media per well. Mixed magnetic beads were added, and the plate was sealed, wrapped in foil, and incubated overnight (18 h) at 4 °C on a plate shaker at 650 rpm. Following incubation, plates were washed three times using a magnetic separation block. Wells were then incubated with 25 µL of detection antibodies for 1 h, followed by incubation with 25 µL Streptavidin-Phycoerythrin (30 min, room temperature). After final washes, 150 µL of Sheath Fluid PLUS was added to each well, and the beads were resuspended on a plate shaker for 5 min. Fluorescence was detected using a Luminex MAGPIX^®^ system with xPONENT^®^ software. Wells with ≥ 35 beads per region and coefficient of variation (CV) < 20% were included in the final analysis.

#### Immunocytochemistry for cleaved caspase 3

Macrophages were cultured on sterile glass coverslips placed in 24-well plates. After removal of conditioned media, cells were washed with PBS and fixed with 250 µL of 3% (w/v) paraformaldehyde in PBS for 10 min at room temperature. Cells were then washed three times with PBS and permeabilized by incubating in 0.2% (v/v) Triton X-100 in PBS for 5 min three times. After two additional PBS washes, cells were blocked with 250 µL of 20% (v/v) goat serum (Sigma-Aldrich) in PBS for 30 min at room temperature. Following one PBS wash, cells were incubated overnight at 4 °C with rabbit anti-cleaved caspase-3 antibody (R&D Systems; AF835; working concentration of 1 µg/mL in 5% (w/v) BSA/PBS). The next day, cells were washed three times in PBS and incubated with 250 µL biotinylated anti-rabbit secondary antibody (Sigma-Aldrich; B7389; 1:200 in 5% (w/v) BSA/PBS) for 30 min at room temperature. After washing, Streptavidin-Alexa Fluor 488 (Vector Laboratories; SA5488; 1:200 in 5% (w/v) BSA/PBS) was added for 30 min in the dark. Cells were washed again and mounted on Superfrost™ glass slides using ProLong™ Gold Antifade Reagent with DAPI. Ten fields per sample, each containing approximately 50 cells, were imaged at 20× magnification. Apoptotic cells (green) were quantified and expressed as a percentage of total cells (DAPI-stained nuclei, blue).

### Statistical analysis

Statistical analysis was performed using GraphPad Prism (versions 9–10.3.0) for macOS. Data are presented as mean ± standard error of the mean (SEM). Normality of data was assessed using a Shapiro-Wilk test prior to selecting statistical tests. For normally distributed data, t-tests or ANOVA with Student-Newman-Keuls *post hoc* tests were used. For non-normal data, appropriate non-parametric tests were applied: Mann-Whitney (unpaired), Wilcoxon signed-rank (paired), or Friedman with Dunn’s multiple comparisons. Paired analyses were used when the same biological samples were tested under different conditions. Statistical significance is reported as *p < 0.05, **p < 0.01, ***p < 0.001, ****p < 0.0001 and † is used when non-parametric tests were applied. All experiments used biological replicates from different individuals with numbers stated in the figure legends.

## Supplementary Information

Below is the link to the electronic supplementary material.

Supplementary Material 1

## Data Availability

Data are provided within the manuscript or supplementary information files.
