# Supplementary material for: Loss of IGFBP-6 promotes monocyte-driven atherogenesis in periodontal disease
Source: Sci Rep. 2026 May 12;16:21658. doi: 10.1038/s41598-026-47023-6 (PMC13357806; doi:10.1038/s41598-026-47023-6)
Supplement: Supplementary file 1 — Supplementary Material 1 [file 41598_2026_47023_MOESM1_ESM.docx]

Loss of IGFBP-6 promotes monocyte-driven atherogenesis in periodontal disease

Dáire Shanahan^1.2*^, Georgios Kremastiotis^1^, Kerry Wadey^1^, Claudine Hodgson^1^, Joon Seong^2^, Nicola West^2^, Angela H. Nobbs^3^ ,Jason Johnson^1^ ,Sarah George^1^

^1^ Laboratory of Cardiovascular Pathology, Translational Health Sciences, Bristol Medical School, Faculty of Health Sciences, University of Bristol, Level 7, Bristol Royal Infirmary, Bristol, BS2 8HW, England, UK

^2^Bristol Dental School, University of Bristol, Bristol BS2 0PT, UK

^3^Bristol Dental School Research Laboratories, Dorothy Hodgkin Building, University of Bristol, Bristol BS1 3NY, UK.

Table S1: Demographics and characteristics of naïve monocyte donors for use in proteomics-Periodontally healthy controls.

Blood collected under REC reference:10/H0107/32

| **Screen number** | **Pooled group number** | **Age at time of sample** | **Sex** | **Ethnicity** | **Smoker** |
| --- | --- | --- | --- | --- | --- |
| 19006 | 126 | 30 | F | White | No |
| 19007 |  | 39 | F | White | No |
| 19010 |  | 60 | F | White | No |
| 19001 | 127N | 32 | M | White | No |
| 19017 |  | 48 | M | White | No |
| 19005 |  | 53 | M | White | No |
| 19002 | 127C | 34 | F | White | No |
| 19008 |  | 47 | F | White | Ex-smoker, currently vaping |
| 19012 |  | 58 | F | White | No |
| 19009 | 128N | 30 | M | Asian | No |
| 19015 |  | 41 | M | White | No |
| 19004 |  | 53 | M | Asian | No |
| 19013 | 128C | 34 | F | White | No |
| 19016 |  | 43 | F | White | No |
| 19011 |  | 52 | F | White | No |

Table S2: Demographics and characteristics of naïve monocyte donors for use in proteomics-Individuals with periodontitis

Blood collected under REC reference: 15/WA/0209

| **Screen number** | **Pooled group number** | **Age at time of sample** | **Sex** | **Ethnicity** | **Type of Periodontitis** | **Smoker** |
| --- | --- | --- | --- | --- | --- | --- |
| P142 | 129N | 37 | F | White | Aggressive | No |
| P144 |  | 46 | F | White | Aggressive | No |
| P127 |  | 55 | F | White | Chronic | No |
| P247 | 130N | 37 | M | Asian | Aggressive | No |
| P180 |  | 48 | M | Asian | Chronic | No |
| P184 |  | 56 | M | White | Chronic | No |
| P203 | 129C | 38 | F | White | Chronic | No |
| P213 |  | 48 | F | White | Chronic | No |
| P093 |  | 56 | F | White | Chronic | No |
| P072 | 131 | 39 | M | White | Aggressive | No |
| P222 |  | 41 | M | White | Chronic | No |
| P224 |  | 61 | M | Not stated | Chronic | No |
| P103 | 130C | 39 | F | White | Aggressive | No |
| P132 |  | 42 | F | White | Chronic | No |
| P139 |  | 57 | F | White | Chronic | No |

Table S3: Demographics and characteristics of naïve monocyte donors with periodontitis for *in vitro* studies

Blood collected under REC reference: 21/PR/1095. The protocol for collecting peripheral blood from these individuals is outlined in a study registered with ISRCTN (Reference: ISRCTN10537092).

Peripheral blood was collected from consenting male (n=11) and female (n=14) donors who were periodontally and medically healthy with mean age ± SD of 35.5±12.39 years and who did not smoke (REC reference: 10/H0107/32). Some individuals donated blood on more than one occasion for different experiments.

| **Screen number** | **Age at time of sample** | **Sex** | **Ethnicity** | **BPE** | **BOP** |
| --- | --- | --- | --- | --- | --- |
| 001 | 43 | F | Asian | 4* 4 4*  4 3 4 | 38.7% |
| 002 | 55 | F | White | 4 4 4  4 3 4 | 85.8% |
| 003 | 39 | M | Not specified | 4 4 4  3 3 4 | 0% |
| 004 | 21 | F | White | 4* 4 4*  4* 4 4* | 100% |
| 005 | 47 | F | White | 4 4 4  4 4 4 | 23.4% |
| 006 | 32 | M | White | 4 4 4  4 4 4 | Not completed |
| 007 | 42 | F | White | 4 4 4  4 4 4 | 20.3% |
| 008 | 46 | F | Asian | 4 4 4  4 4 4 | Not completed |
| 009  *Screen fail | 47 | Not specified | Black | 4* 4 4*  4* 4 4* | Not completed |
| 011 | 37 | F | White | 4 4 4  4 4 4 | 14.1% |
| 012 | 40 | M | White | 4 4 4  4 4 4 | 40.7% |
| 013 | 17 | Not specified | White | 1 4 4  4 2 3 | 10.1% |
| 014 | 50 | M | White | 4 4 4  4 3 4 | 5.9% |
| 015 | 60 | F | White | 3 1 3  3 3 2 | Not completed |
| 016 | 51 | F | White | 4 4 4  4 4 4 | Not completed |
| 017 | 54 | F | White | 4* 4 4*  3* 4 4* | No completed |

BPE= Basic Periodontal Examination. BOP=bleeding on probing.

Table S4: Demographics and characteristics of Human Coronary Artery Endothelial Cell (HCAEC) for *in vitro* studies

Promocell; catalogue number: C-12221

| Lot Number | Age | Sex | Race | Tissue Origin |
| --- | --- | --- | --- | --- |
| 458Z035.3 | 61 | Female | Caucasian | Coronary artery |
| 447Z026.1 | 63 | Male | Caucasian | Coronary artery |

Table S5: Details of primers used for RT-qPCR

| **Gene** | **Primer Sequence** | **Company** |
| --- | --- | --- |
| *ABCG1* | Forward: ATCTCCTATGTCAGGTATGG | Sigma |
|  | Reverse: AGGGAGATGAAGAAAATCCC |  |
| *ABCA1* | Forward: GTGTTTCTGGATGAACCC | Sigma |
|  | Reverse: TTCCATTGACCATGATTGC |  |
| *SR-A1* | Forward: AAGACCAGTACAACTCACTG | Sigma |
|  | Reverse: GCCTTTTAATGAGATGGTAGC |  |
| *CD36* | Forward: AGCTTTCCAATGATTAGACG | Sigma |
|  | Reverse: GTTTCTACAAGCTCTGGTTC |  |
| *LOX1* | Forward: AATGATAGAAACCCTTGCTC | Sigma |
|  | Reverse: AGTCCTGAACAATTTGCTAC |  |
| *IGFBP-6* | Forward: AGAATCCTAAGGAGAGTAAACC | Sigma |
|  | Reverse: CACTGAGTCCAGATGTCTACGG |  |

**Western blot statement**

Full-length, uncropped Western blot images corresponding to all cropped blots presented in the main figures are provided in this Supplementary Information. All images are shown as originally acquired from the imaging system without adjustment of brightness or contrast. Cropped panels in the main manuscript were derived directly from these full-length blots.

| 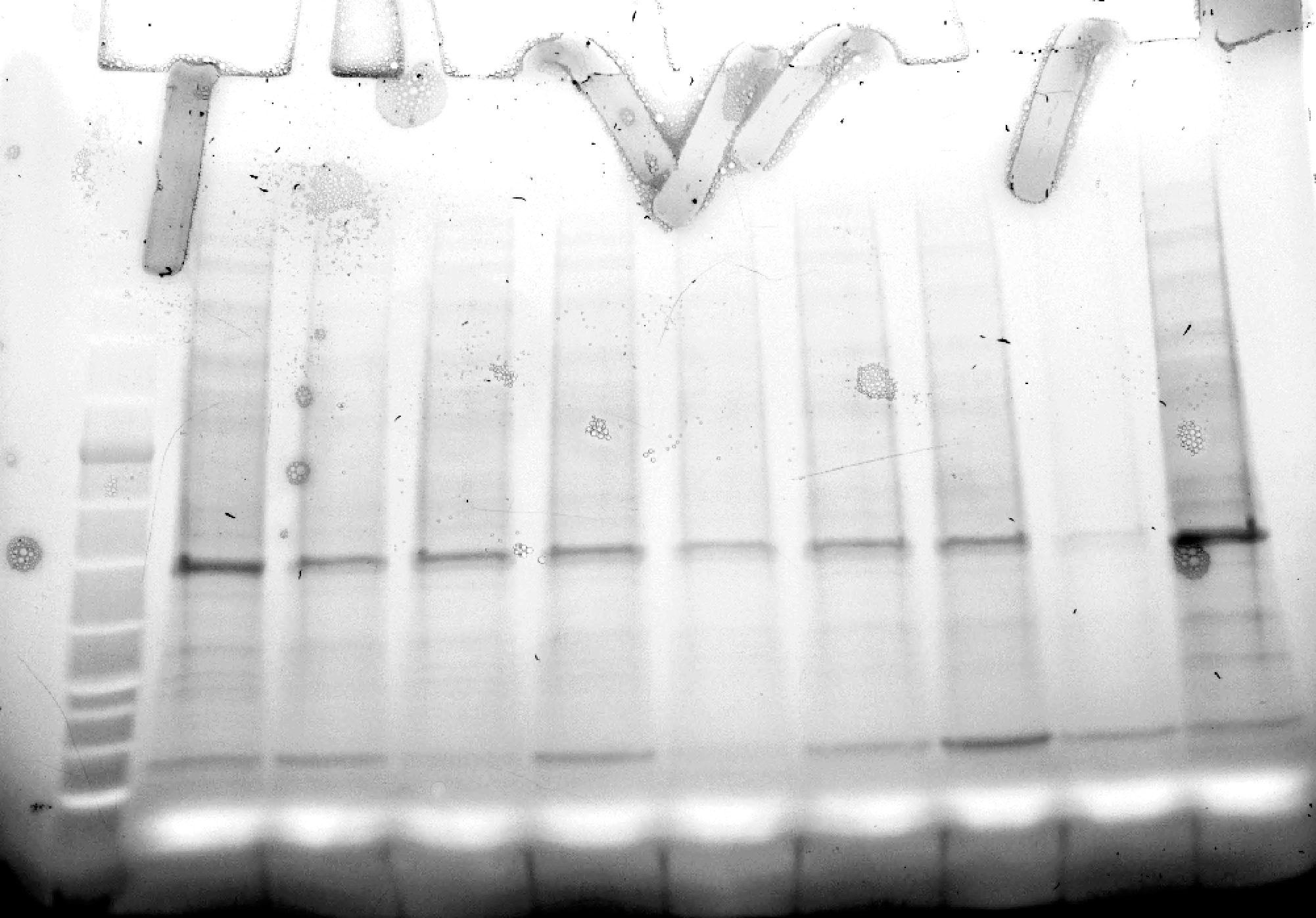 Control  Periodontitis 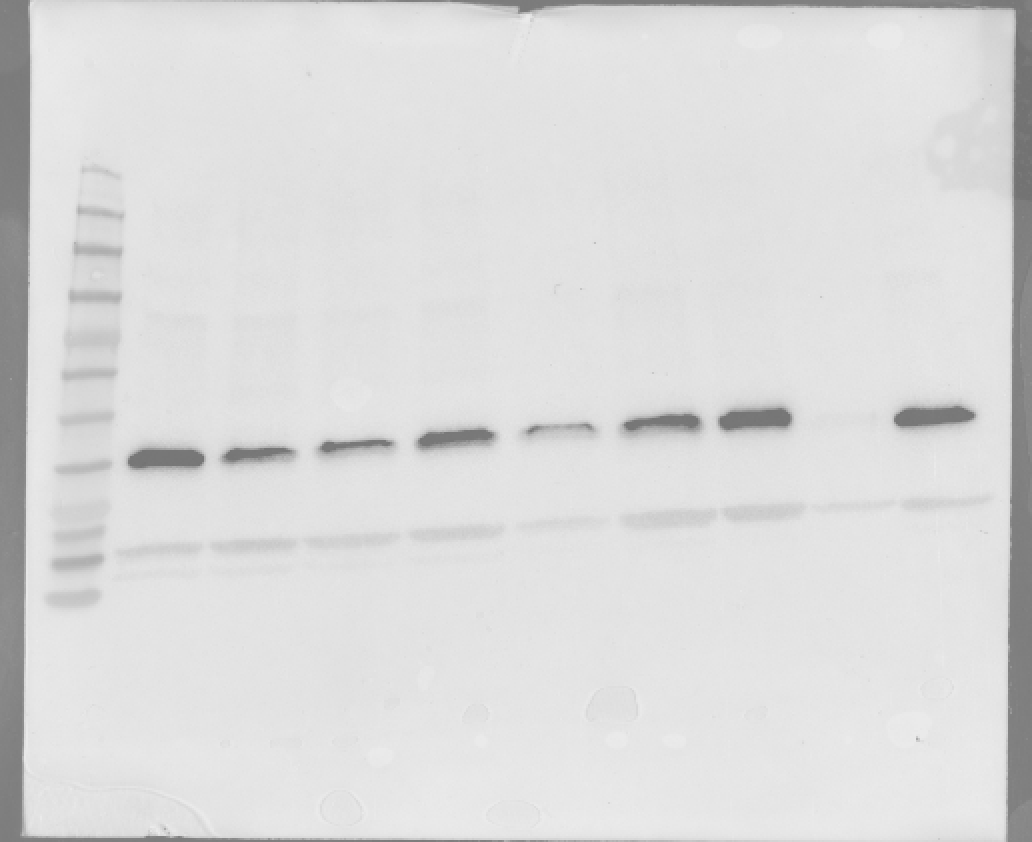 Control  Periodontitis  IGFBP-6  34kDa    Stain free gel  Chemiluminescence |
| --- |

**Figure S1: Full length Western blots and stain-free gels to accompany Figure 1(E).**

Representative full-length Western blot images are shown in their original, unprocessed form. Black boxes indicate the regions corresponding to the cropped panels presented in the main manuscript.

Periodontitis

Periodontitis

Periodontitis

Control

Control

Control

Control

Periodontitis

Periodontitis

Chemiluminescence


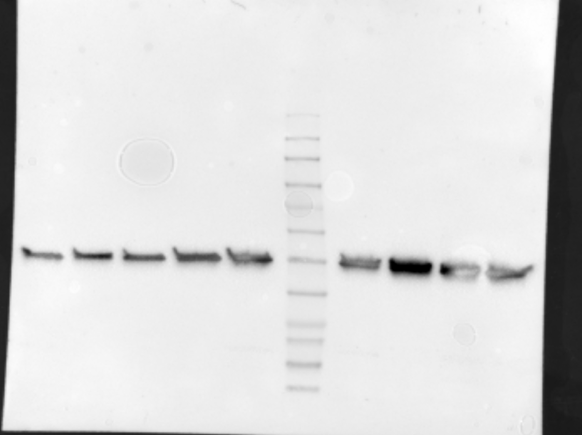


Periodontitis

Periodontitis

Periodontitis

Control

Control

Control

Control

Periodontitis

Periodontitis

β-actin

42kDa

**
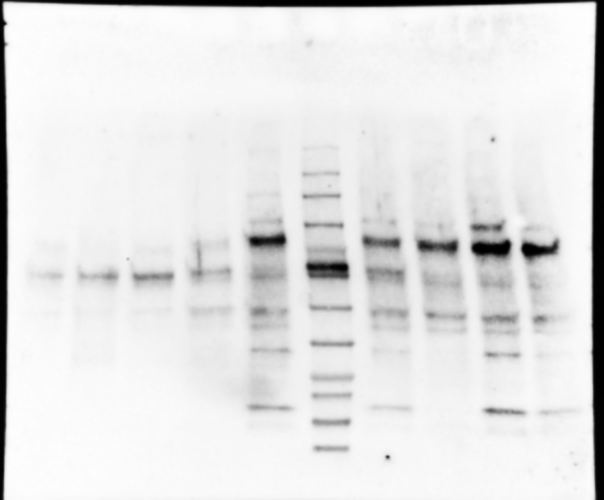
**

Egr-1

75kDa

β-actin

**Figure S2: Full length Western blots and stain-free gels to accompany Figure 1(H).**

Representative full-length Western blot images are shown in their original, unprocessed form. Black boxes indicate the regions corresponding to the cropped panels presented in the main manuscript.

| Control  Periodontitis 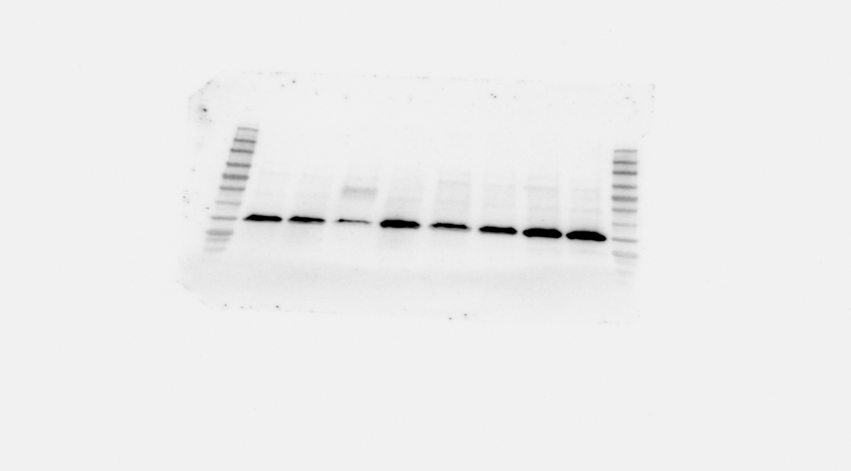 Control  Periodontitis  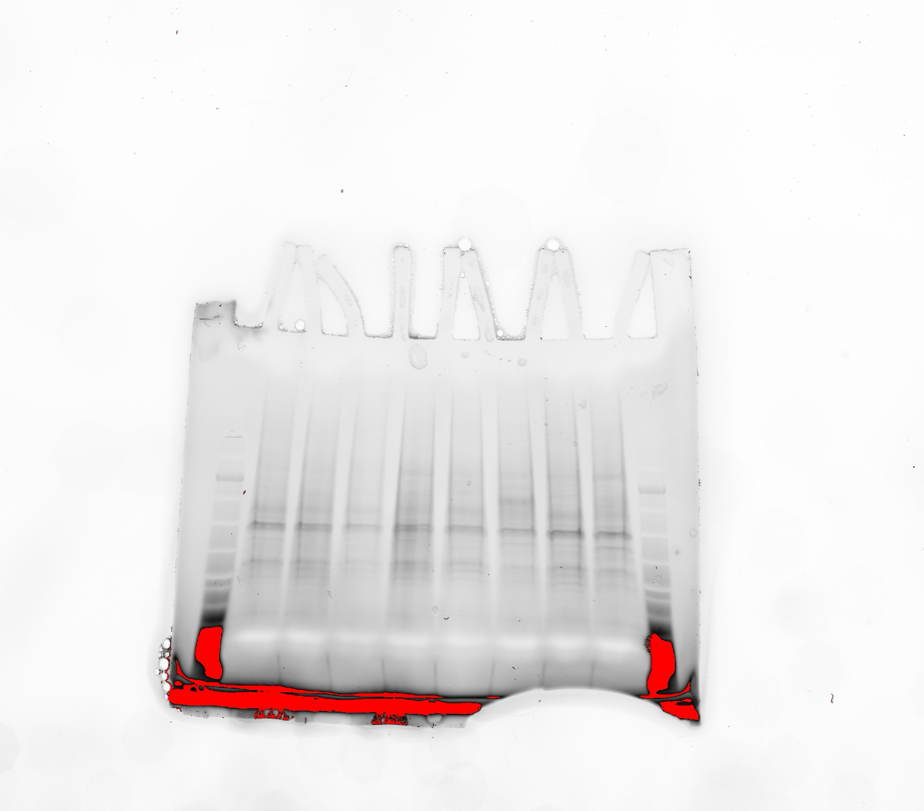  IGFBP-6  34kDa  IGFBP-6  34kDa  Stain free gel  Chemiluminescence |
| --- |

**Figure S3: Full length Western blots and stain-free gels to accompany Figure 1(J).**

Representative full-length Western blot images are shown in their original, unprocessed form. Black boxes indicate the regions corresponding to the cropped panels presented in the main manuscript. Quantification was performed across independent biological replicates as described in the main figure.

Macrophage

Foam cell


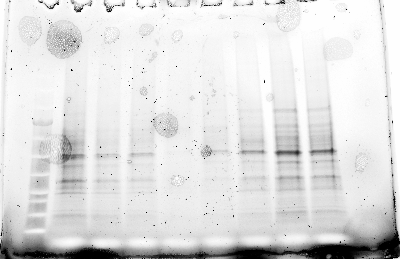


| 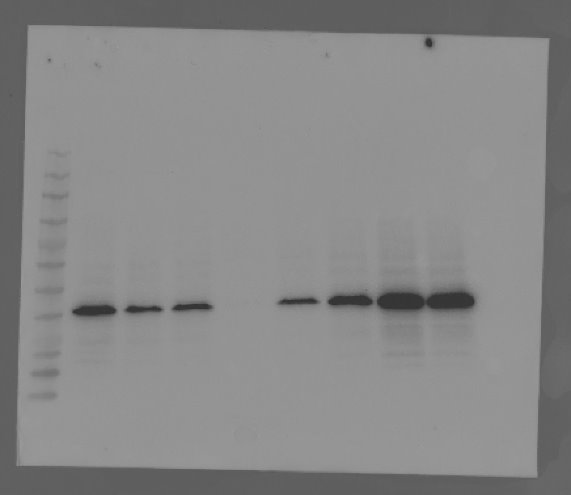 Macrophage  Foam cell    IGFBP-6  34kDa  Stain free gel  Chemiluminescence |
| --- |

**Figure S4: Full length Western blots and stain-free gels to accompany Figure 1(L).**

Representative full-length Western blot images are shown in their original, unprocessed form. Black boxes indicate the regions corresponding to the cropped panels presented in the main manuscript.

| 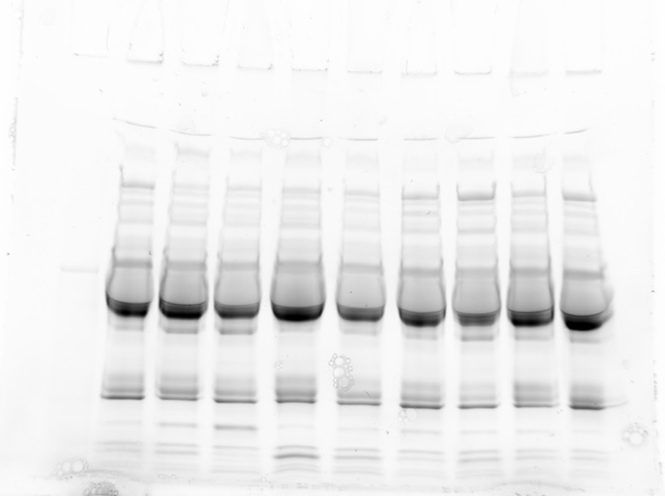 Stain free gel  IGFBP-6  34kDa 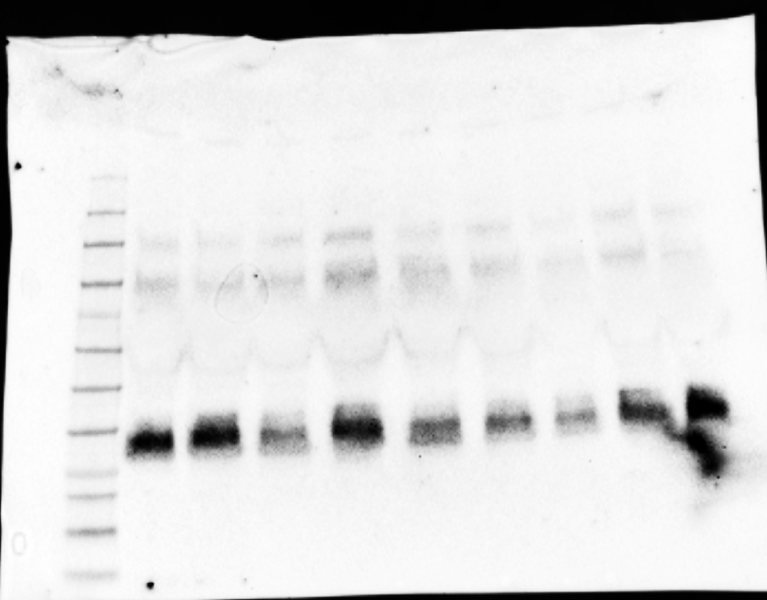 Chemiluminescence  Periodontitis  Periodontitis  Periodontitis  Periodontitis  Control  Control  Control  Control  Periodontitis  Periodontitis  Periodontitis  Periodontitis  Control  Control  Control  Control |
| --- |

**Figure S5: Full length Western blots and stain-free gels to accompany Figure 1(N).**

Representative full-length Western blot images are shown in their original, unprocessed form. Black boxes indicate the regions corresponding to the cropped panels presented in the main manuscript. Lane order in the cropped panels shown in the main manuscript has been adjusted for clarity of presentation (control versus periodontitis); the full-length blots reflect the original lane arrangement. No image manipulation was performed beyond cropping.

Control

Pg 100:1

Pg 50:1

Pg 20:1

β-actin

45kDa


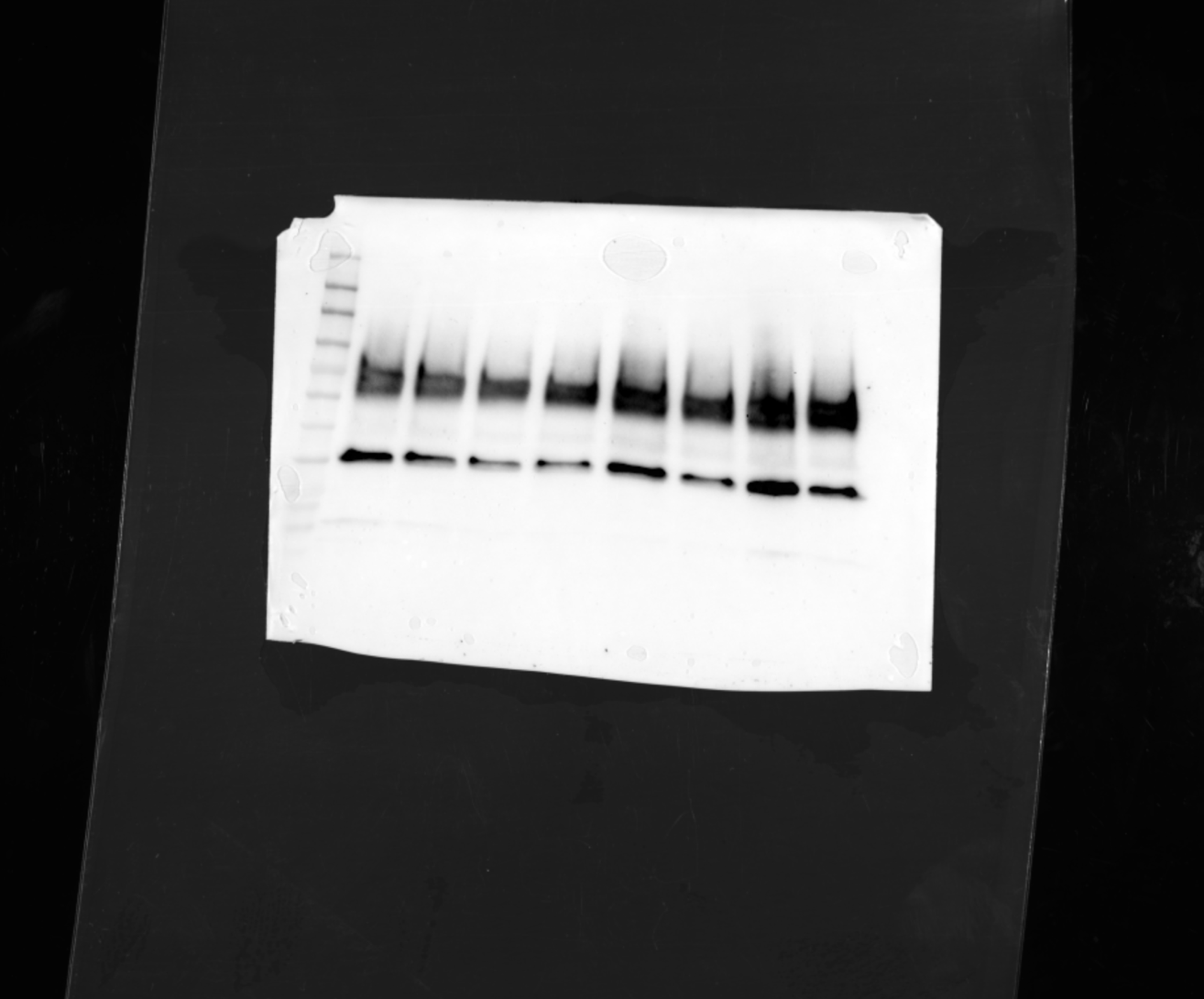


Control

Pg 100:1

Pg 50:1

Pg 20:1

IGFBP-6

34kDa


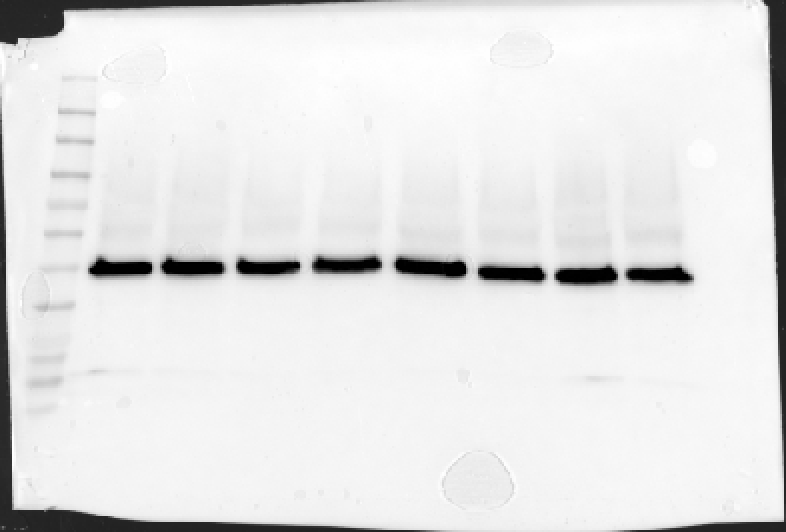


CD226


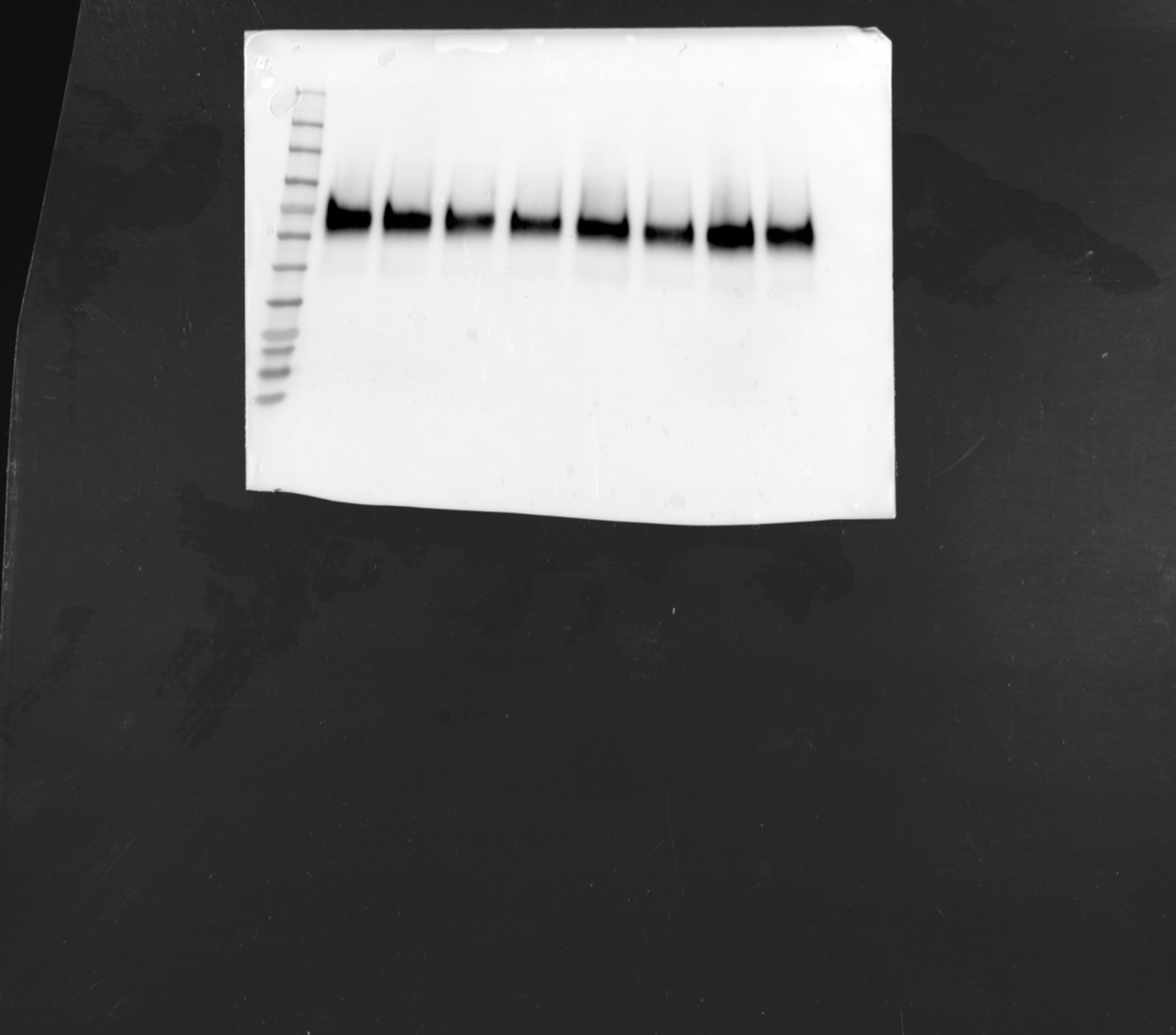


Control

Pg 100:1

Pg 50:1

Pg 20:1

CD226

**Figure S6: Full length Western blots and stain-free gels to accompany Figure 2(B).**

Representative full-length Western blot images are shown in their original, unprocessed form. Black boxes indicate the regions corresponding to the cropped panels presented in the main manuscript.Membranes were initially probed for CD226 and subsequently stripped and reprobed for IGFBP-6. Molecular weight markers are shown.

Control

1μg/ml LPS

0.1μg/ml LPS

0.01μg/ml LPS

| Control  1μg/ml LPS  0.1μg/ml LPS  0.01μg/ml LPS 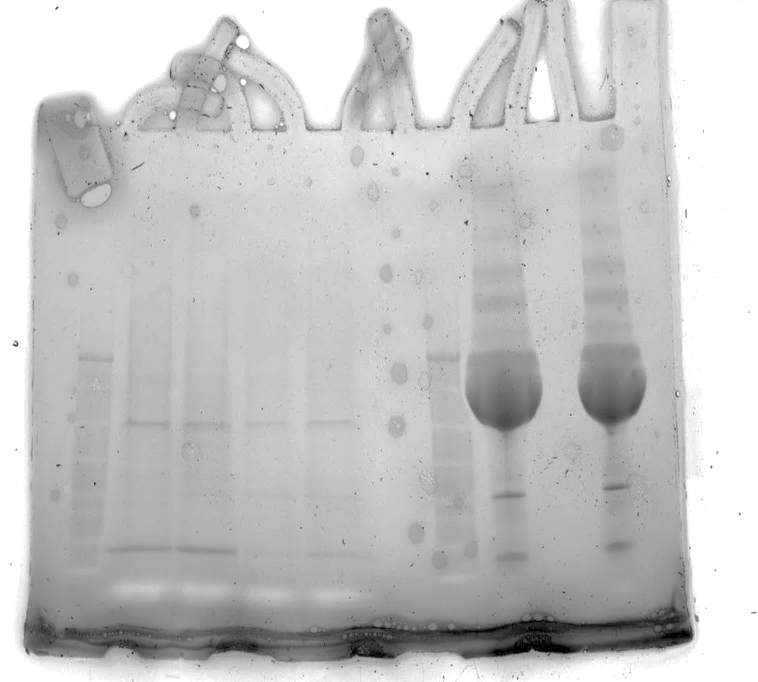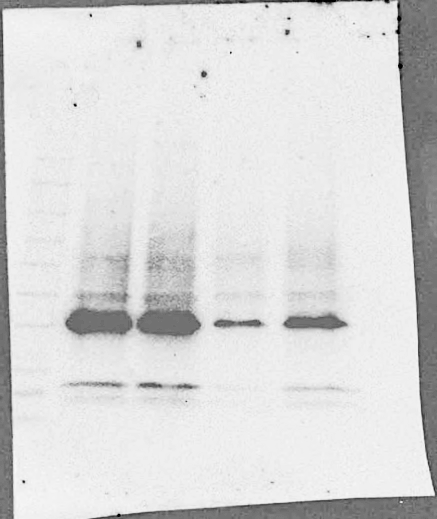 IGFBP-6  34kDa  Chemiluminescence  Stain free gel |
| --- |

**Figure S7: Full length Western blots and stain-free gels to accompany Figure 2(D).**

Representative full-length Western blot images are shown in their original, unprocessed form. Black boxes indicate the regions corresponding to the cropped panels presented in the main manuscript. The IGFBP-6 western blots shown in Figures 2B were derived from dose-response optimisation experiments and therefore include representative lanes of the controls and optimal doses (1 μg/mL) from the same gels.

| Control  1 μg/ml LPS  10 μg/ml LPS  0.01 μg/ml LPS  0.1 μg/ml LPS  Control  1 μg/ml LPS  10 μg/ml LPS  0.01 μg/ml LPS  0.1 μg/ml LPS  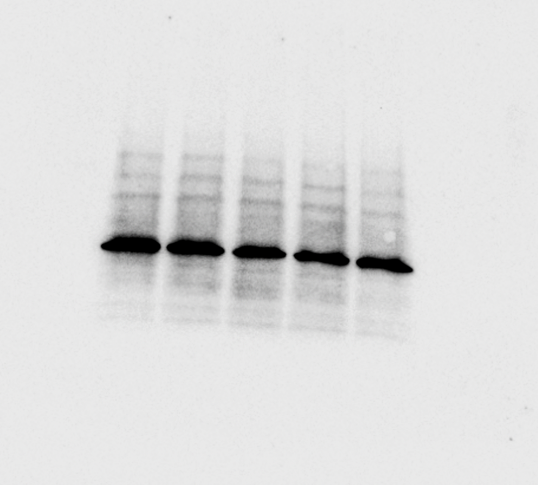 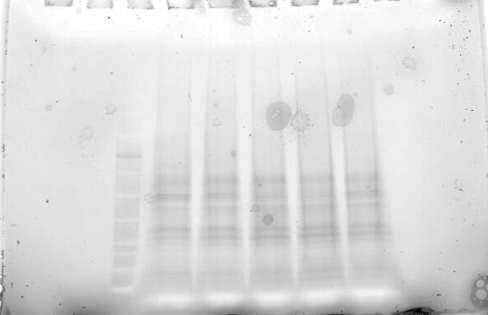 IGFBP-6  34kDa  Stain free gel  Chemiluminescence |
| --- |

**Figure S8: Full length Western blots and stain-free gels to accompany Figure 2(F).**

Representative full-length Western blot images are shown in their original, unprocessed form. Black boxes indicate the regions corresponding to the cropped panels presented in the main manuscript. The IGFBP-6 western blots shown in Figures 2D were derived from dose-response optimisation experiments and therefore include representative lanes of the controls and optimal doses (0.1μg/mL) from the same gels.

0.1 μg/ml LPS

Control

| Control  0.1 μg/ml LPS  1 μg/ml LPS  1 μg/ml LPS 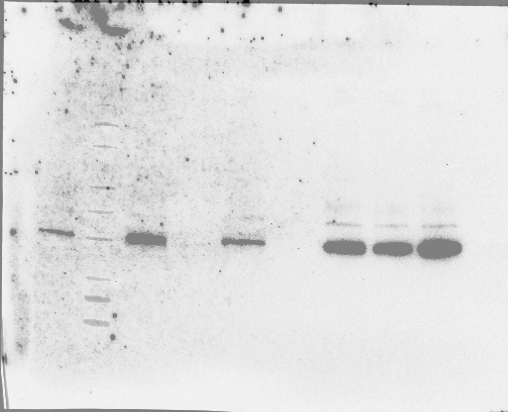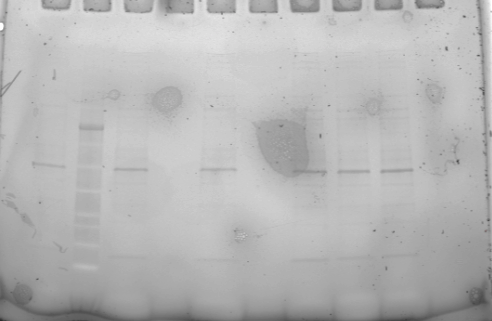   IGFBP-6  34kDa  Chemiluminescence  Stain free gel |
| --- |

**Figure S9: Full length Western blots and stain-free gels to accompany Figure 2(H).**

Representative full-length Western blot images are shown in their original, unprocessed form. Black boxes indicate the regions corresponding to the cropped panels presented in the main manuscript. The IGFBP-6 western blots shown in Figures 2F were derived from dose-response optimisation experiments and therefore include representative lanes of the controls and optimal doses (1 μg/mL) from the same gels.

| Control  0.1 μg/ml LPS  Control  0.1 μg/ml LPS  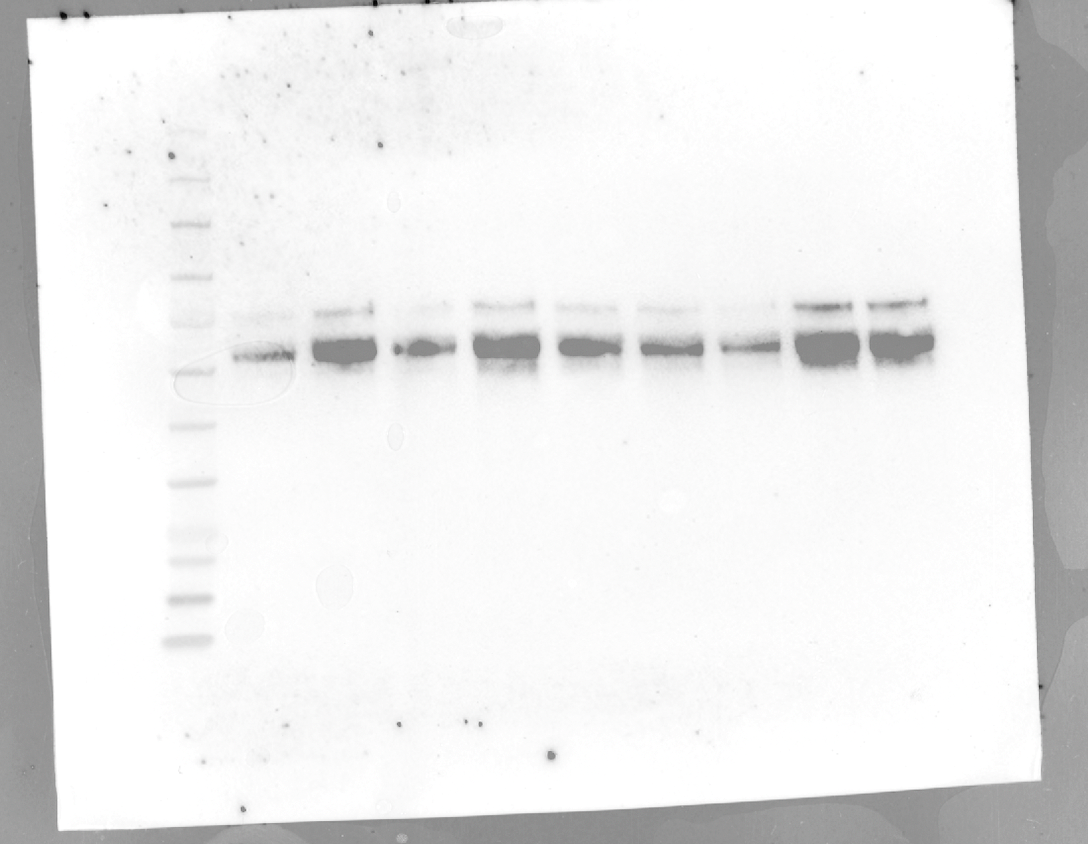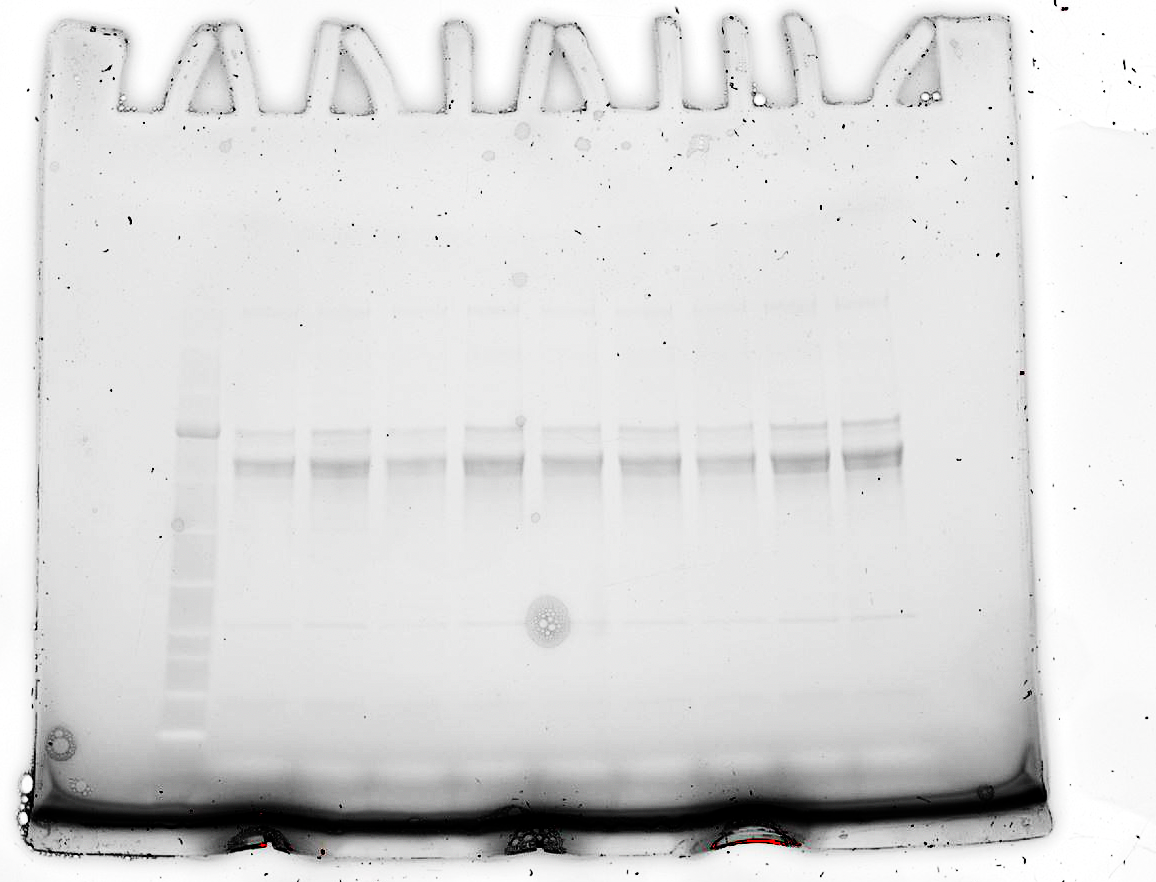  IGFBP-6  70kDa  Stain free gel  Chemiluminescence |
| --- |

**Figure S10: Full length Western blots and stain-free gels to accompany Figure 2(J).**

Representative full-length Western blot images are shown in their original, unprocessed form. Black boxes indicate the regions corresponding to the cropped panels presented in the main manuscript.

| Control  1 μg/ml LPS  10 μg/ml LPS  0.01 μg/ml LPS  0.1 μg/ml LPS 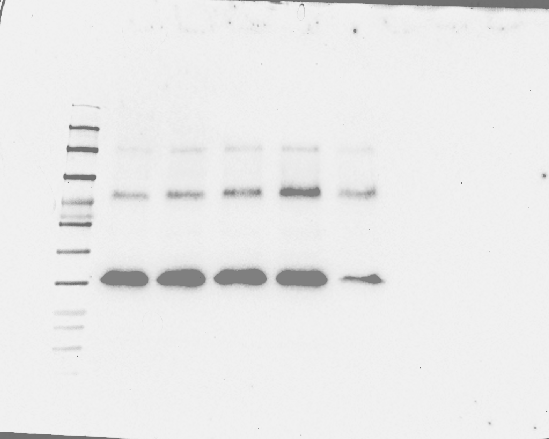 Control  1 μg/ml LPS  10 μg/ml LPS  0.01 μg/ml LPS  0.1 μg/ml LPS 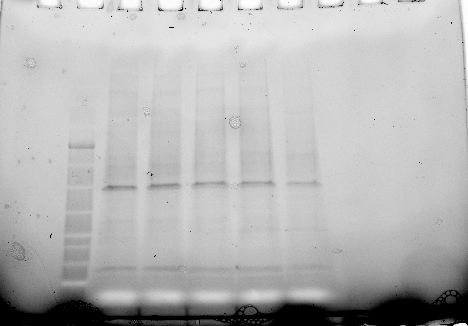 Egr-1  75kDa  Stain free gel  Chemiluminescence |
| --- |

**Figure S11: Full length Western blots and stain-free gels to accompany Figure 2(K).**

Representative full-length Western blot images are shown in their original, unprocessed form. Black boxes indicate the regions corresponding to the cropped panels presented in the main manuscript. A band observed at ~34 kDa corresponds to IGFBP-6; this signal was detected after reprobing the same blot. The Erg-1 western blots shown in Figures 2J were derived from dose-response optimisation experiments and therefore include representative lanes of the controls and optimal doses (1 μg/mL) from the same gels.

| 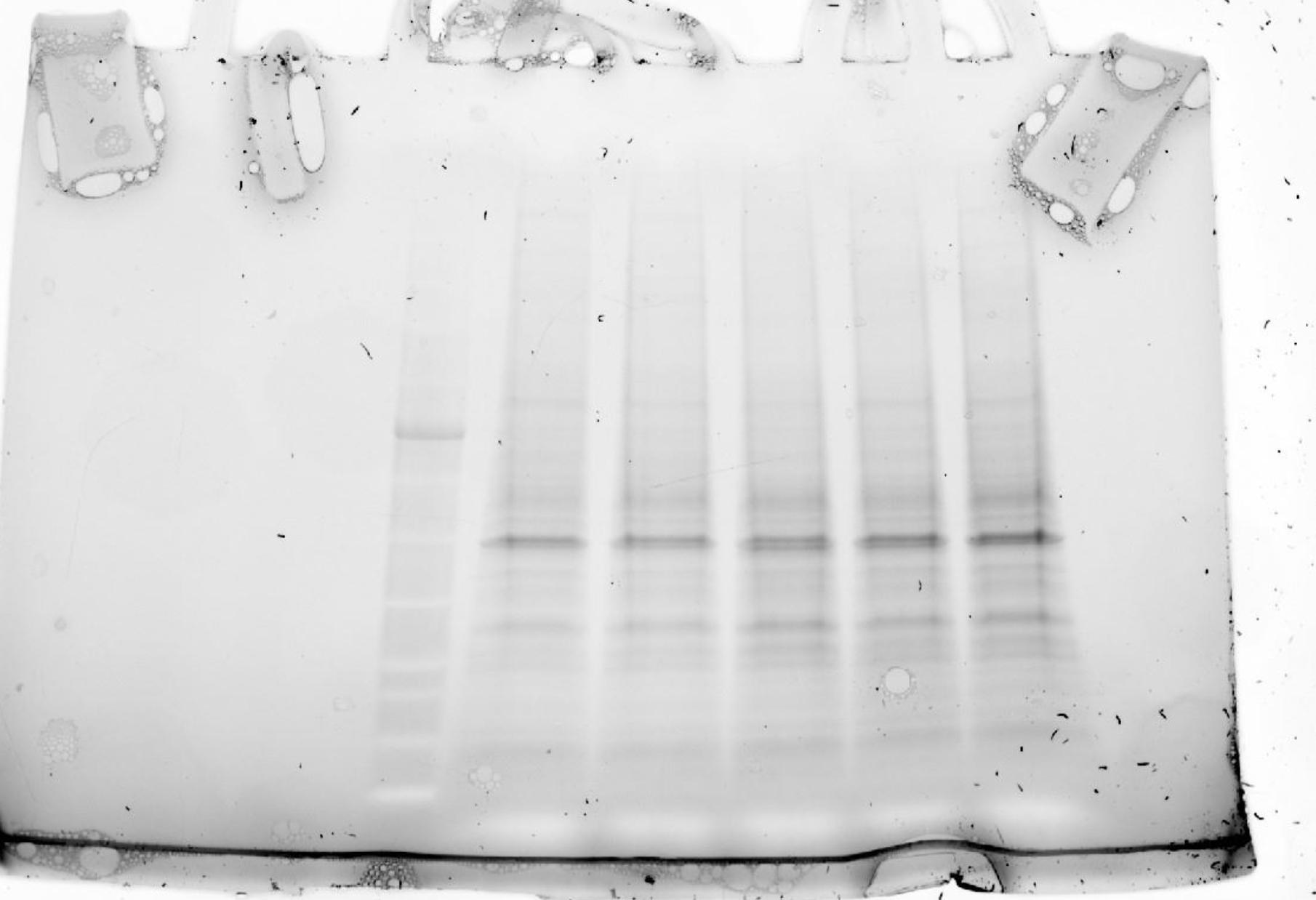 Control  0.1 μg/ml LPS  LPS + rIGFBP-6  LPS + rIGFBP-6  Control  0.1 μg/ml LPS  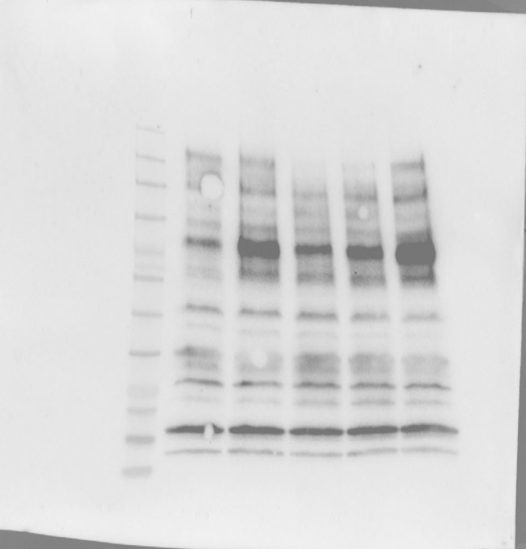  Egr-1  75kDa  Chemiluminescence  Stain free gel |
| --- |

**Figure S12: Full length Western blots and stain-free gels to accompany Figure 2(N) and Figure 3(D).**

Representative full-length Western blot images are shown in their original, unprocessed form. Black boxes indicate the regions corresponding to the cropped panels presented in the main manuscript. For Figure 2(L), the Western blot was cropped to show the control and *P. gingivalis* LPS-treated lanes for Egr-1 protein. For Figure 3(D), the cropped blot includes lanes for control, *P. gingivalis* LPS, and *P. gingivalis* LPS with 1 μg/ml recombinant IGFBP-6 (rIGFBP-6). Additional lanes present on the full blot correspond to experimental conditions not included in the final analysis and are shown here for transparency.

| MP  EXO  SP  Ladder  rIGFBP-6  1 µg/ml  MP  EXO  SP  Ladder  rIGFBP-6  1 µg/ml  IGFBP-6  34kDa  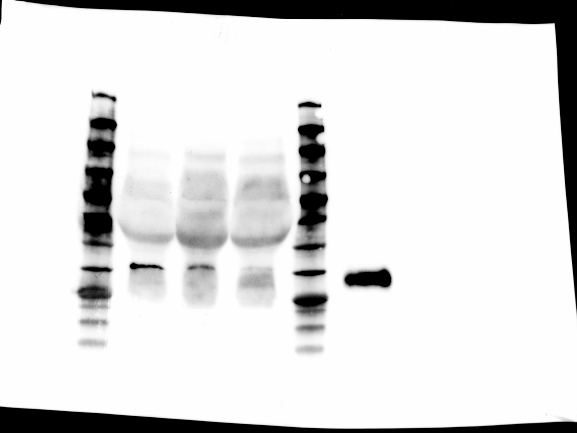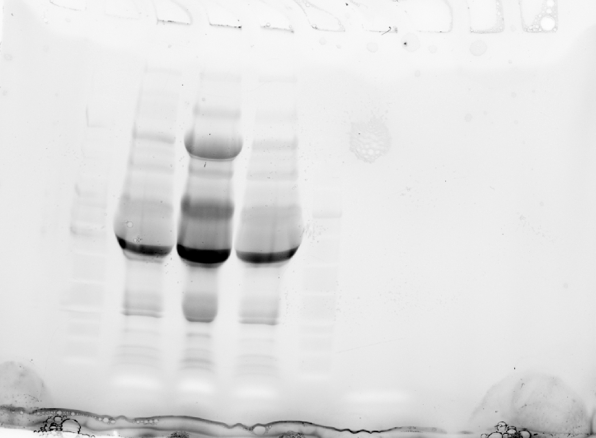  Stain free gel  Chemiluminescence |
| --- |

**Figure S13: Full length Western blots and stain-free gels to accompany Figure 3(A).**

Representative full-length Western blot images are shown in their original, unprocessed form. Black boxes indicate the regions corresponding to the cropped panels presented in the main manuscript.

| 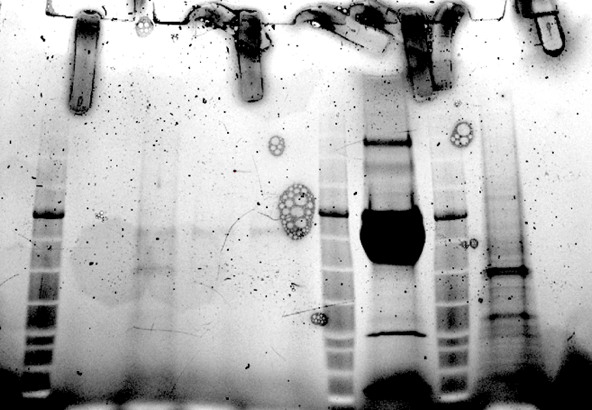  Stain free gel  MP  EXO  SP  Ladder  MP  EXO  SP  Ladder  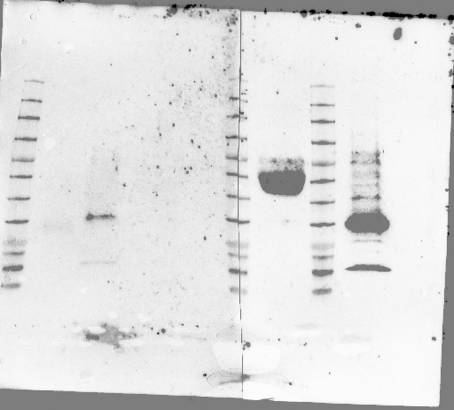  IGFBP-6  34kDa  Chemiluminescence |
| --- |

**Figure S14: Full length Western blots and stain-free gels to accompany Figure 3(B).**

Representative full-length Western blot images are shown in their original, unprocessed form. Black boxes indicate the regions corresponding to the cropped panels presented in the main manuscript.

| Control  LPS  LPS + rIGFBP-6  LPS + MP  Control  LPS  LPS + rIGFBP-6  LPS + MP  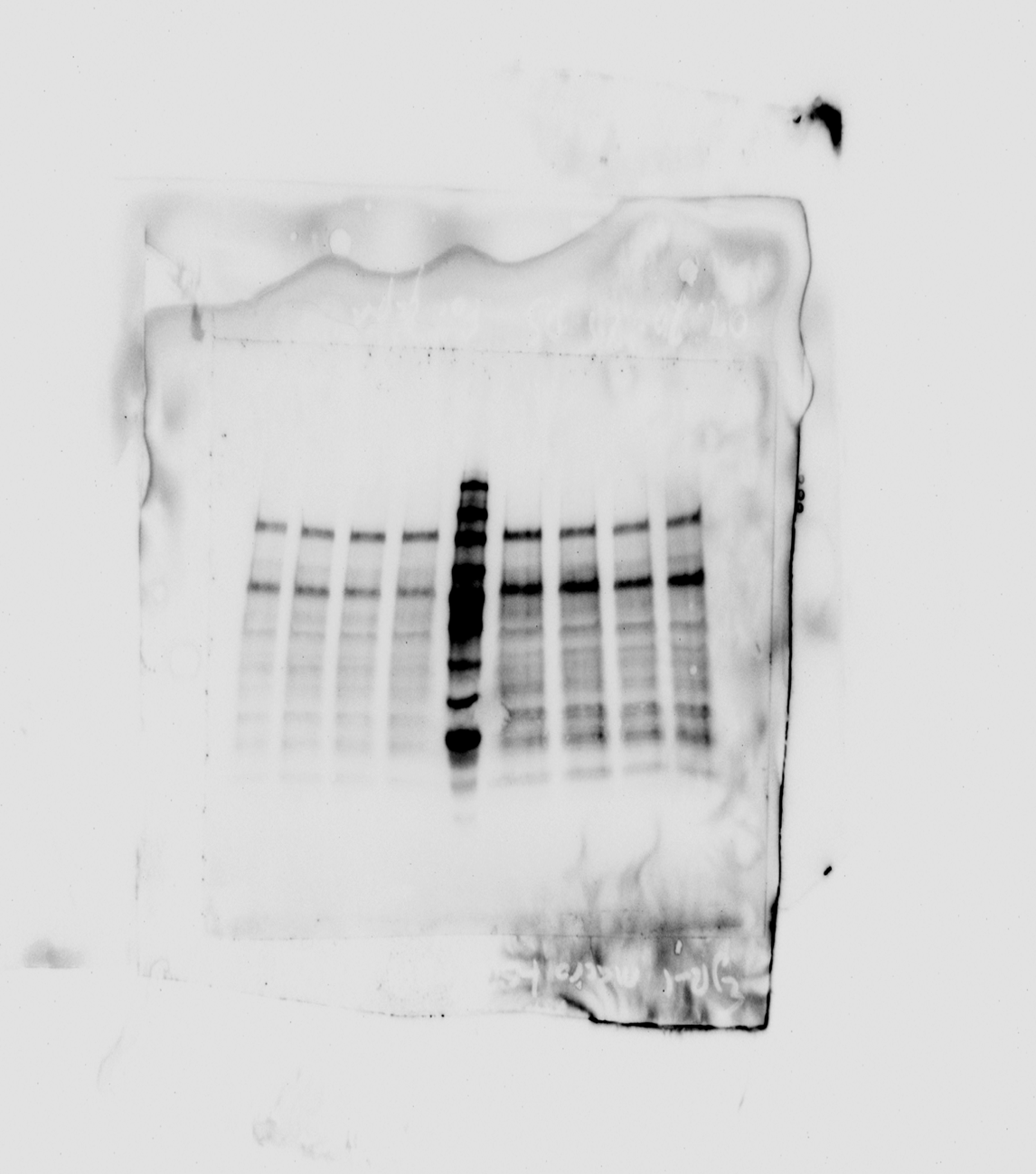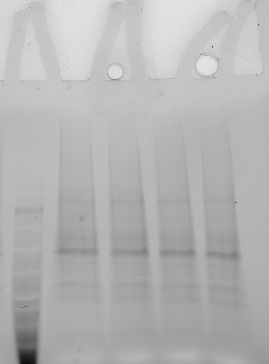  Egr-1  75kDa  Chemiluminescence  Stain free gel |
| --- |

**Figure S15: Full length Western blots and stain-free gels to accompany Figure 3(F).**

Representative full-length Western blot images are shown in their original, unprocessed form. Black boxes indicate the regions corresponding to the cropped panels presented in the main manuscript.

| β-tubulin  55kDa 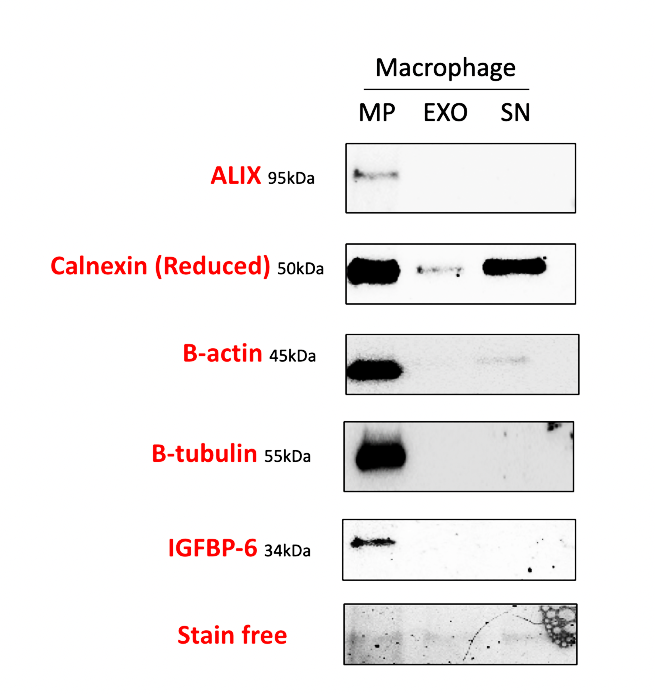 ALIX  95kDa  Stain Free  IGFBP-6  34kDa  β-actin  45kDa 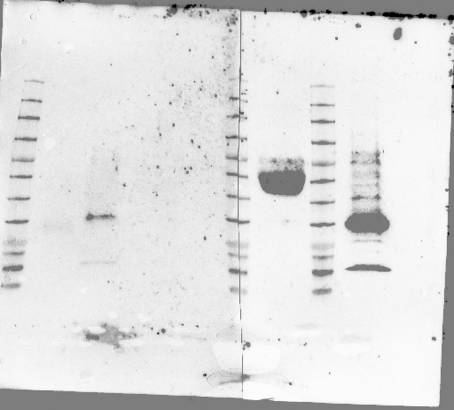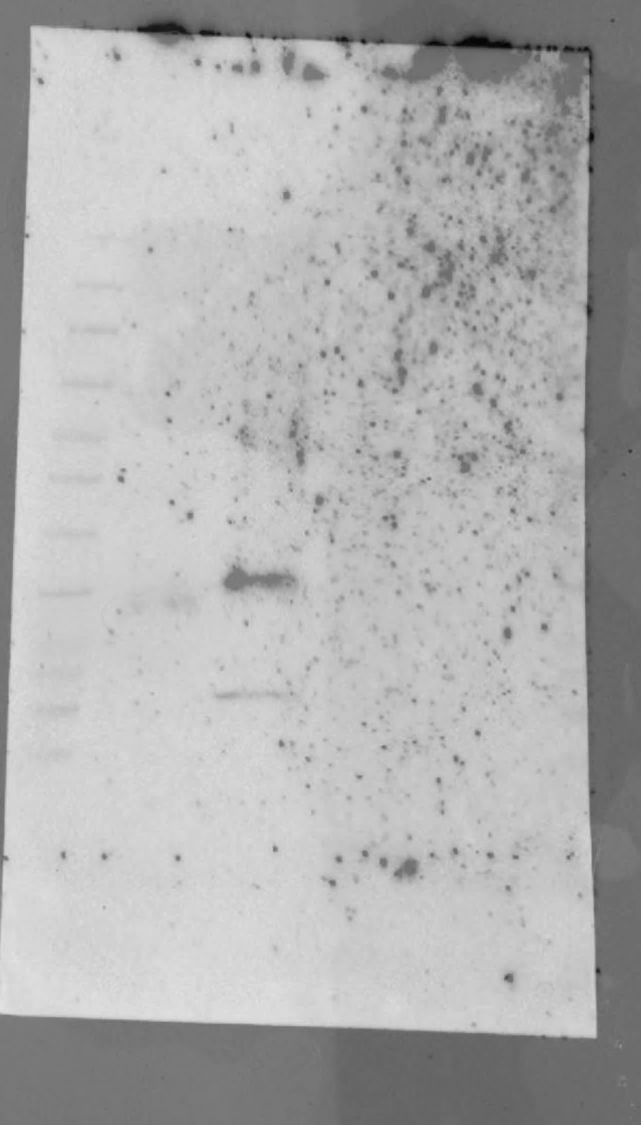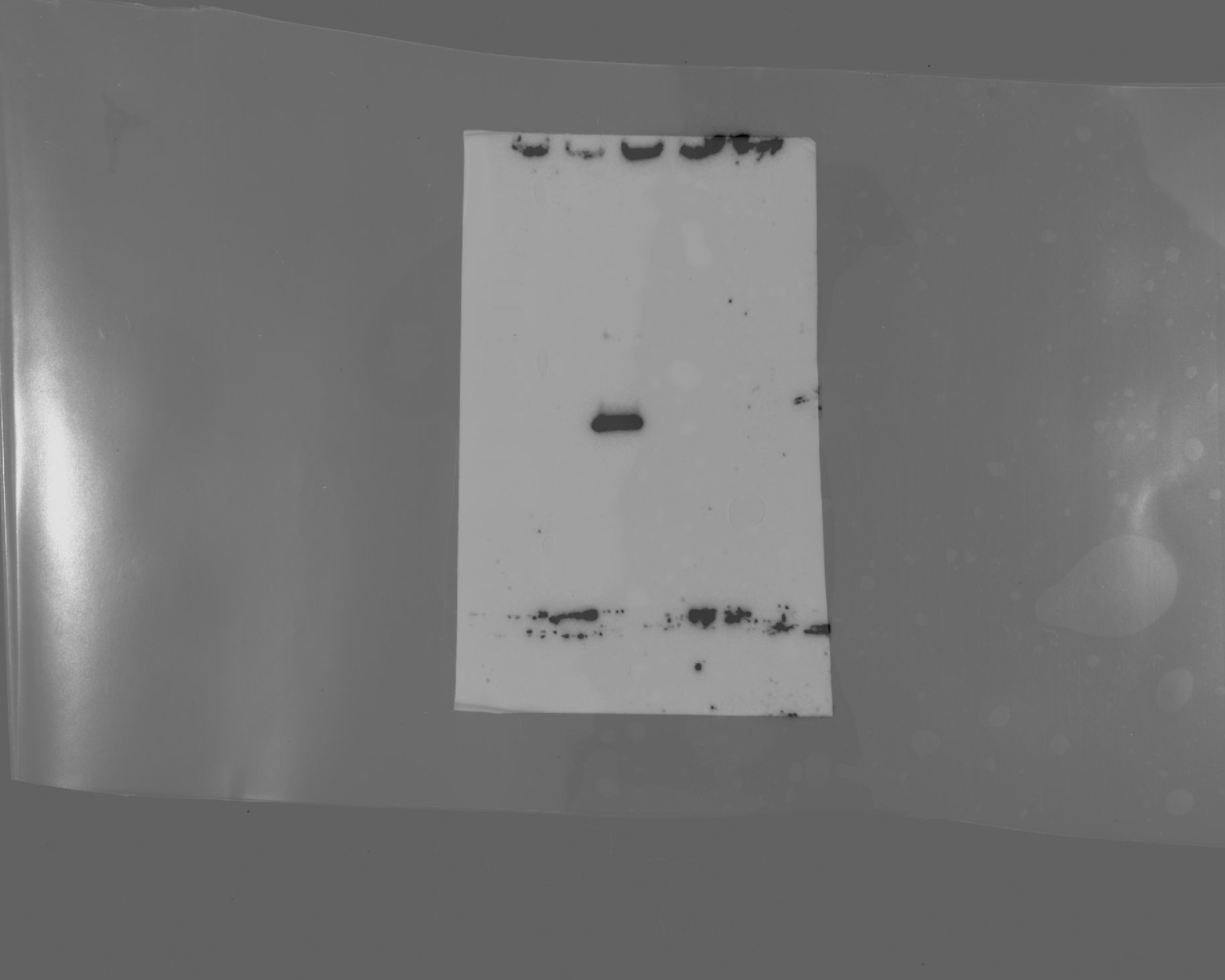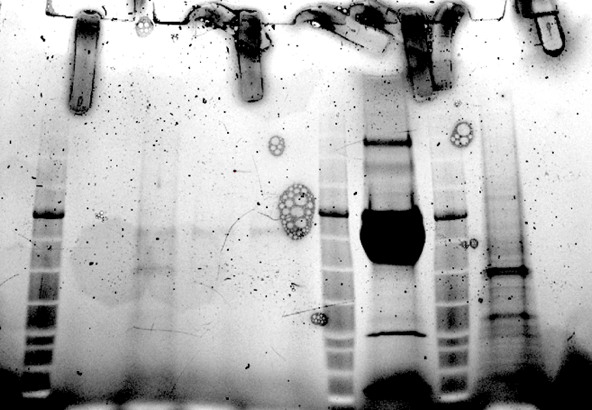 (A)  MP  EXO  SP  Ladder  (E)  (D)  MP  EXO  SP  Ladder  MP  EXO  SP  Ladder  MP  EXO  SP  Ladder |
| --- |
| (C)  (B)  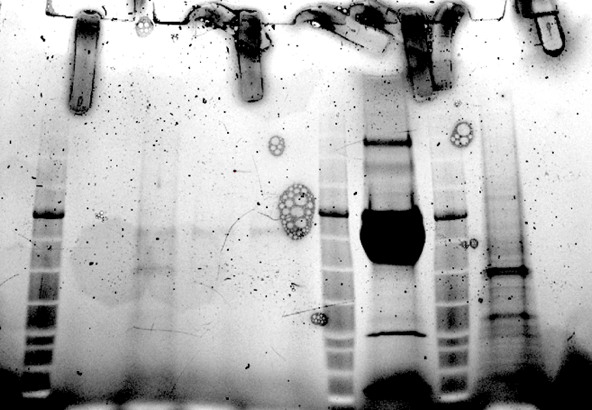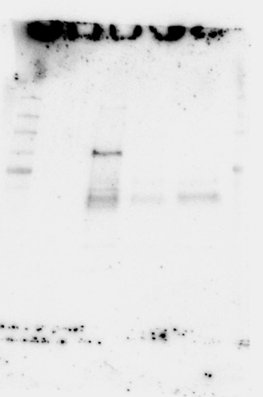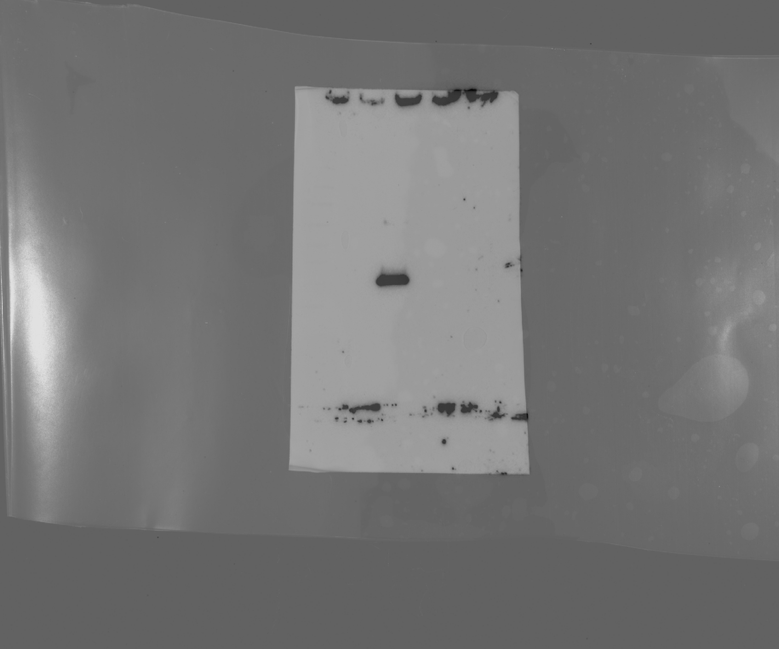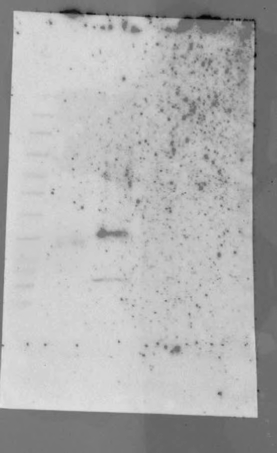  ALIX  Stain free gel  β-actin  β-tubulin |

**Figure S16: Characterization of extracellular vesicles (EVs) isolated from LPS-stimulated macrophages.**

Macrophages were treated with 0.1 µg/mL P. gingivalis LPS for 2 h. Conditioned media were collected and processed via differential ultracentrifugation to isolate extracellular vesicle subtypes, including microparticles (MPs) and exosomes (EXOs). **(A)** Western blotting was performed to assess EV marker expression and the localization of IGFBP-6. IGFBP-6 was detected exclusively in the MP fraction and was absent from the exosome (EXO) and supernatant (SN) fractions, suggesting selective packaging within MPs. The EV preparation was characterized by Western blot using: ALIX (an exosome-enriched marker involved in ESCRT-mediated biogenesis), β-tubulin (a cytoskeletal protein commonly found in large EVs), and β-actin (a general cytoskeletal marker used to assess vesicle integrity and loading). Stain-free gel images served as loading controls. Full-length, uncropped blots corresponding to **(B)** stain free gel, **(C)** β-actin,**(D)** β-tubulin, and **(E)** ALIX are shown in their original, unprocessed form. Black boxes indicate the regions corresponding to the cropped panels shown in panel (A). Full-length, uncropped IGFBP-6 blots corresponding to these experiments are provided in Figure S14. Arrowheads indicate detected bands; molecular weights are shown in kDa. MP, microparticles; EXO, exosomes; SN, supernatant

| (A)  †    † |
| --- |
| 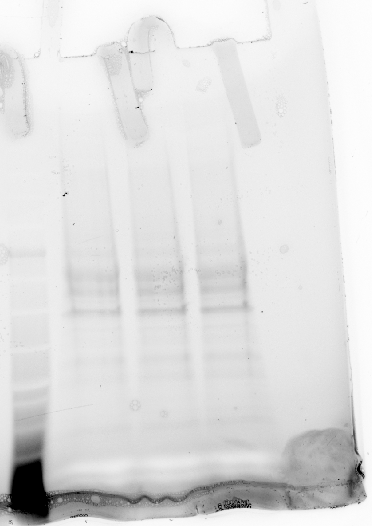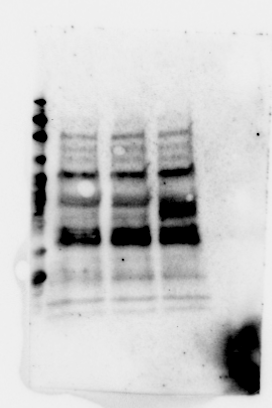  Control  IgG  nIGFBP-6  Erg-1  74kDa  Stain Free  (B) |
| (C) 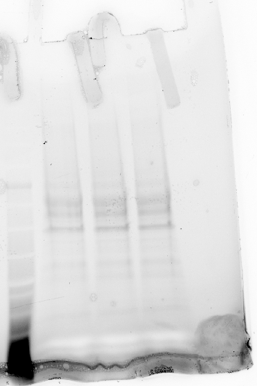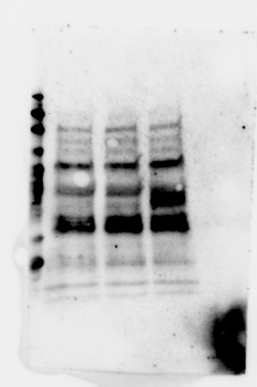 Egr-1  75kDa  Stain free gel  Chemiluminescence |

**Figure S17: Validation of neutralising IGFBP-6 (n-IGFBP-6) antibody in macrophage-derived foam cells.**

Macrophages from healthy individuals (n = 8) were incubated with 10 μg/ml oxLDL for 24 h in the presence of either 5μg/ml neutralising IGFBP-6 antibody (nIGFBP-6), an IgG control antibody, or left untreated (control). IGF signalling activation was assessed by quantifying Egr-1 protein levels as a downstream readout. **(A)** Densitometric analysis of Egr-1 protein normalised to total protein using stain-free gel technology shows a significant increase in Egr-1 expression following nIGFBP-6 treatment compared to both controls (p=0.0080). Data are presented as fold change relative to control. ns, not significant or †p < 0.05 using Friedman’s test with Dunn’s *post hoc* analysis. Error bars represent SEM. **(B)** Representative Western blot of Egr-1 protein expression and corresponding stain-free total protein loading control. Arrowheads indicate the Egr-1 band at ~74 kDa, additional lower molecular weight bands likely represent non-specific binding or degradation products and were not included in densitometric analysis. **(C)** Representative full-length Western blot images are shown in their original, unprocessed form. Black boxes indicate the regions corresponding to the cropped panels presented in panel (B).

| (A)  (B)  (C)  (D)  (I)  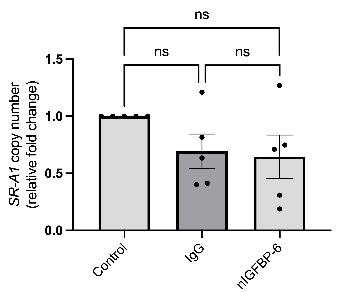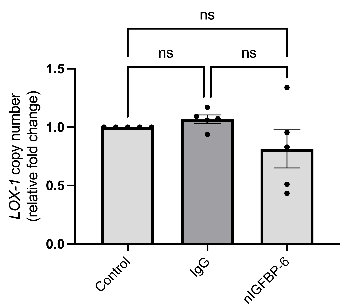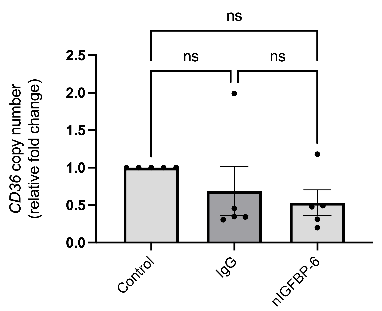  (H)  (F)  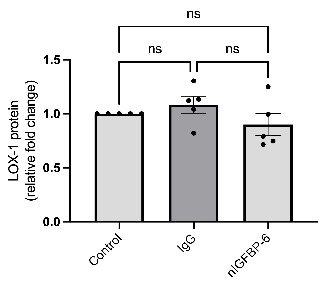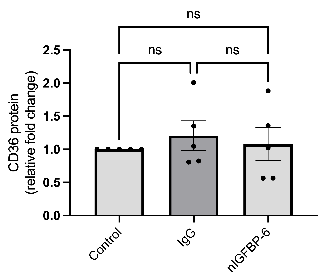  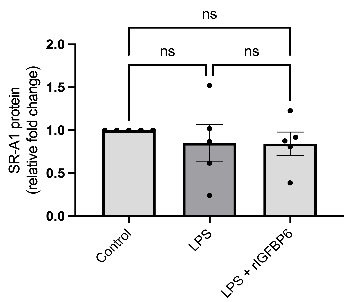  (G)  (E)  Control  IgG  nIGFBP-6  Stain  Free  SR-A1  50kDa 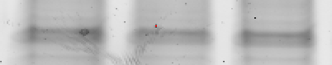 CD36  80kDa  Stain  Free  Control  IgG  nIGFBP-6  Control  IgG  nIGFBP-6 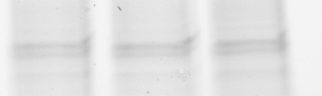 Stain  Free 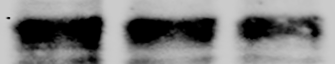 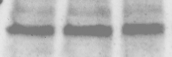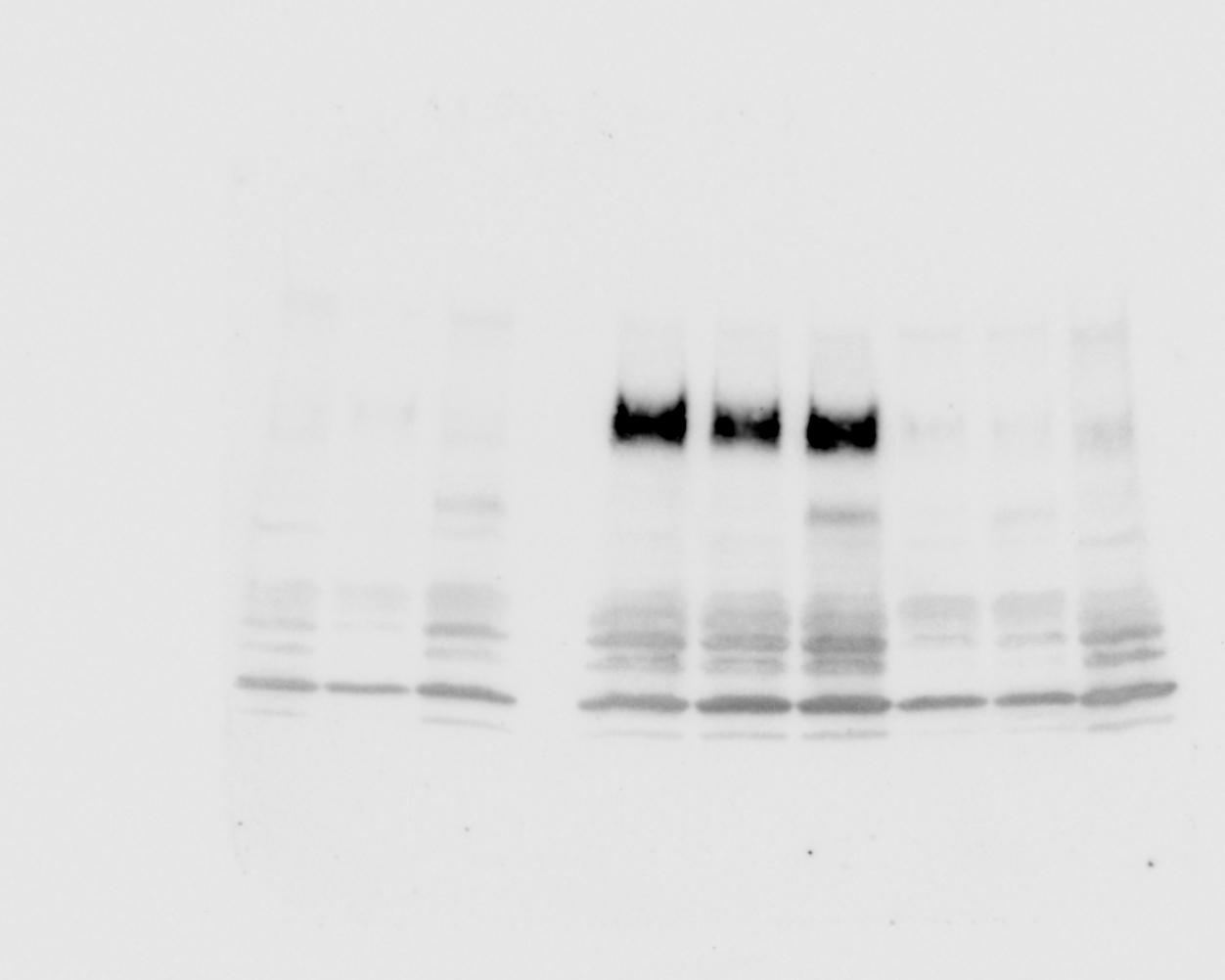  LOX-1  31kDa  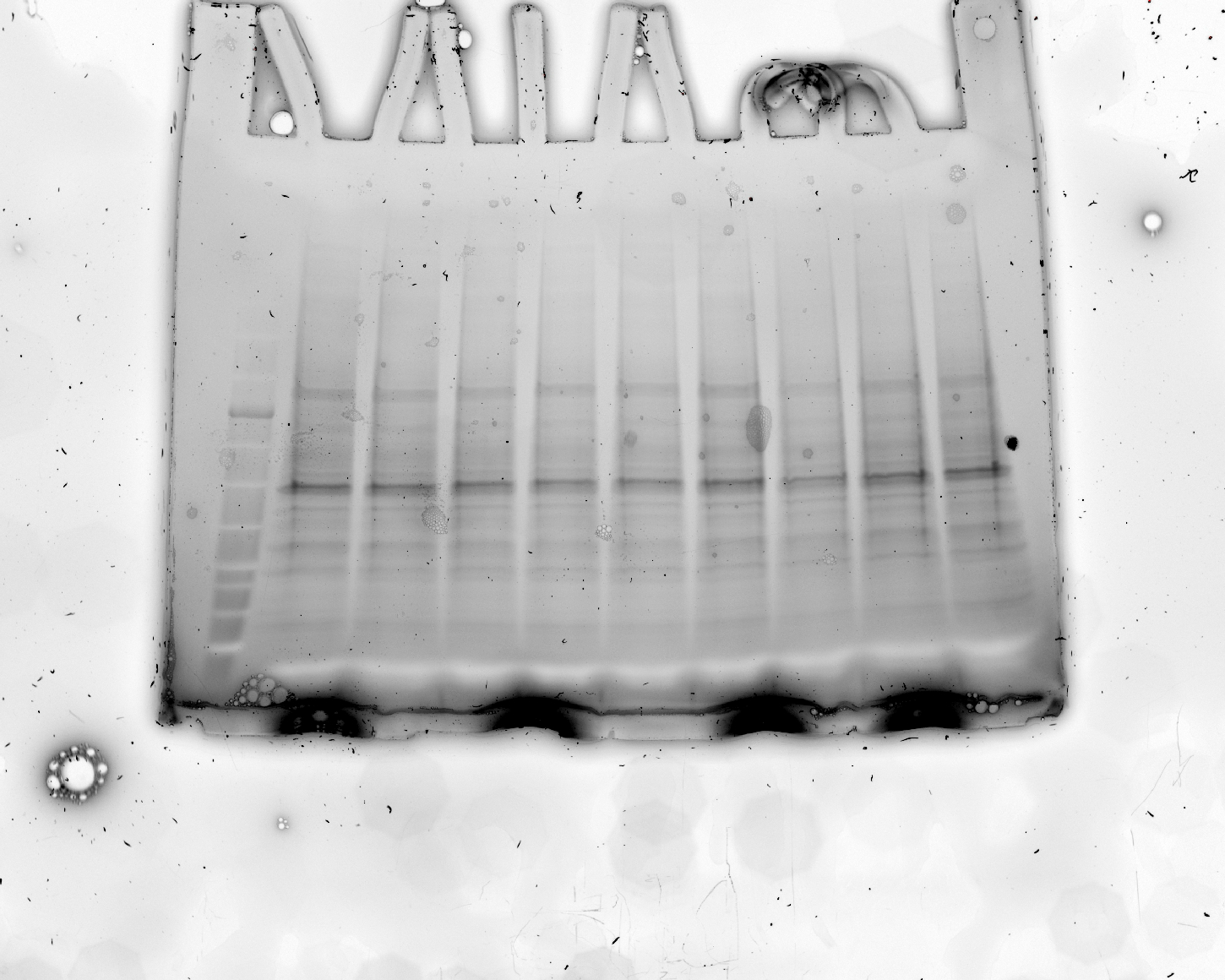 |
| --- |
| 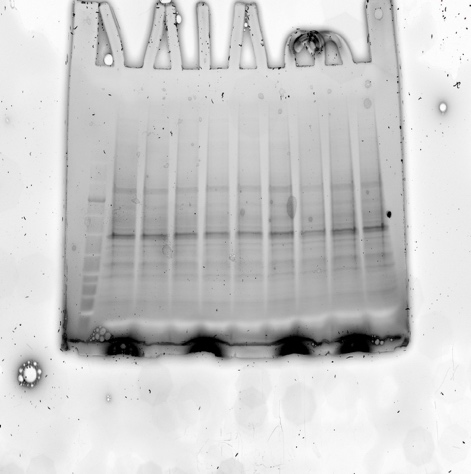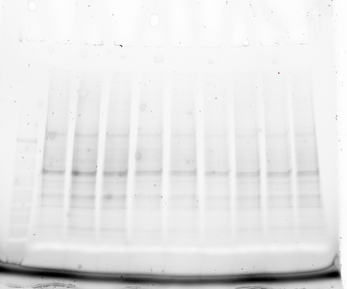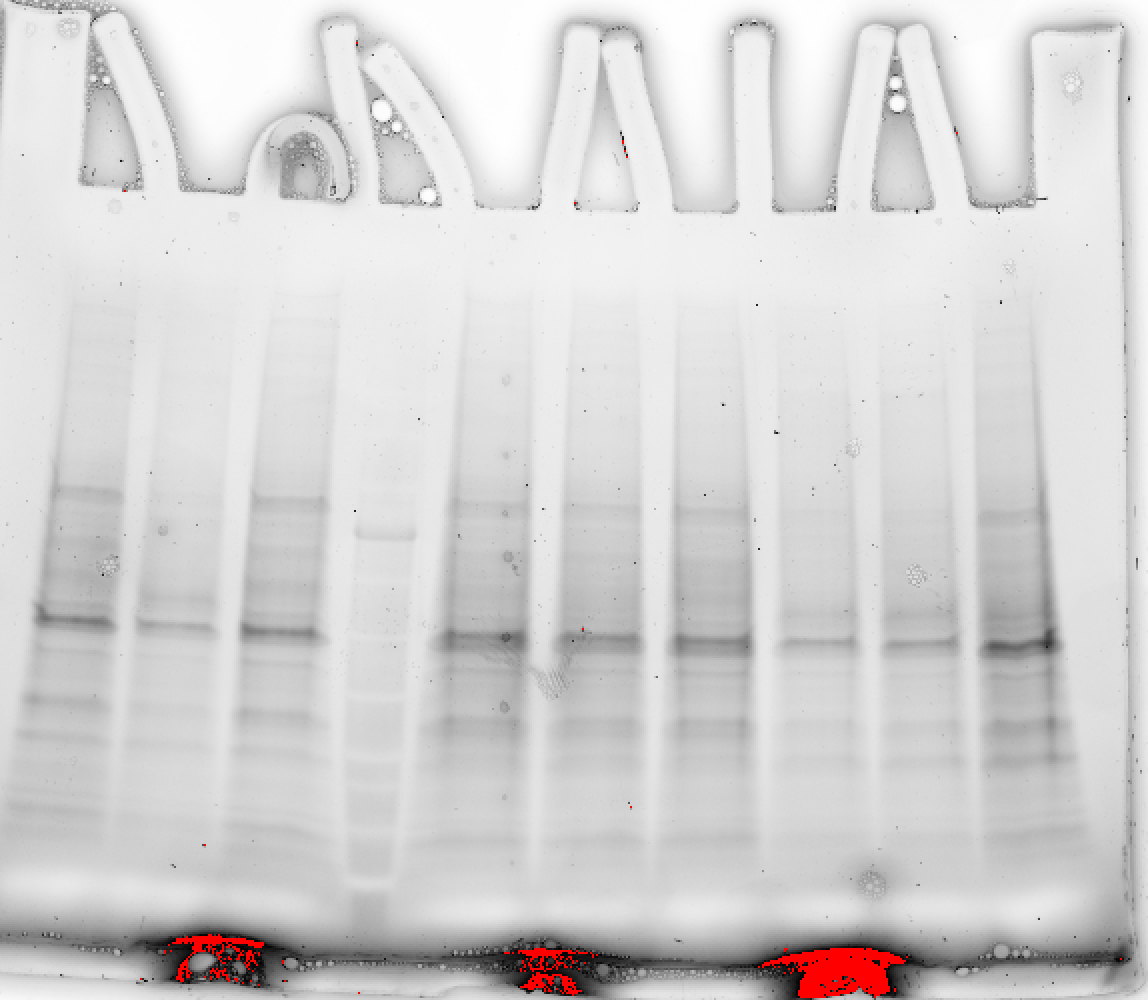  (L)  (K)  (J)  Stain free gel  Stain free gel  Stain free gel  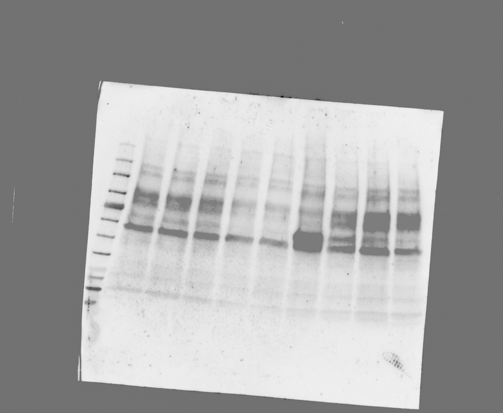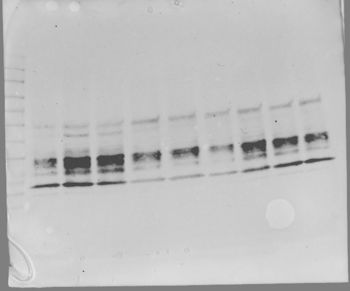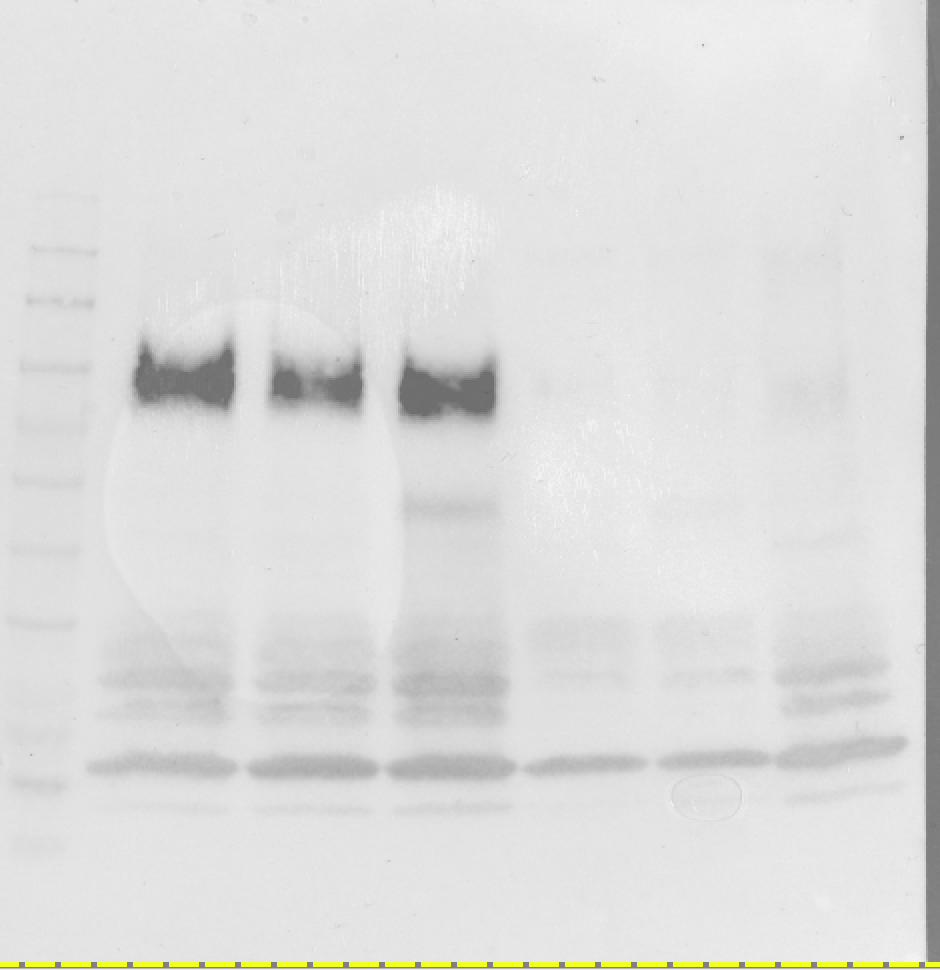  LOX-1  31kDa  Chemiluminescence  SR-A1  50kDa  Chemiluminescence  Chemiluminescence |

**Figure S18: The effects of neutralising IGFBP-6 protein on the expression of scavenger receptors in macrophage-derived foam cells.**

CD36

80kDa

Macrophages were cultured with 10 μg/ml oxLDL in the presence of 5 μg/ml neutralising IGFBP-6 (nIGFBP-6) antibody, 5 μg/ml IgG control, or left untreated. mRNA expression was analysed at 6 h by qPCR for **(A)** CD36 (p=0.3673; n=5), **(B)** LOX-1 (p=0.1821; n=5), and **(C)** SR-A1 (p=0.3673; n=5), and is shown as copy number per ng RNA relative to untreated controls. Protein expression was measured at 24 h by Western blotting for **(D, E)** CD36 (p=0.6914; n=5), **(F,G)** LOX-1 (p=0.1821; n=5), and **(H,I)** SR-A1 (p=0.5216; n=5), normalised to stain-free gel total protein loading controls. Representative full -length Western blot images corresponding to **(J)** CD36, **(K)** LOX-1,and **(L)** SR-A1 are shown in their original, unprocessed form. Black boxes indicate the regions corresponding to the cropped panels presented in panels (E, G and I).Arrowheads indicate the detected protein bands; molecular weights are shown in kDa. ns, not significant, using Friedman’s test with Dunn’s *post hoc* test. Error bars indicate SEM.

| nIGFBP-6  IgG  Control  Control  nIGFBP-6  IgG  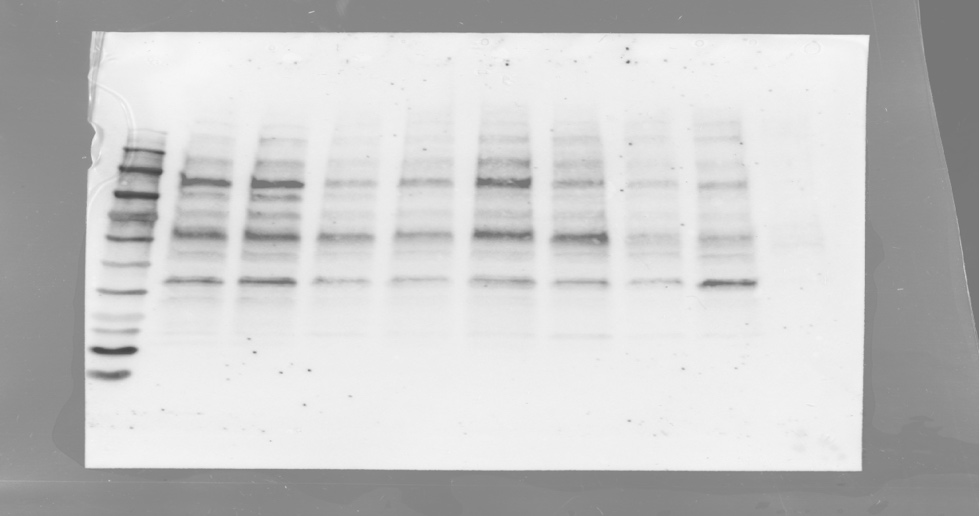 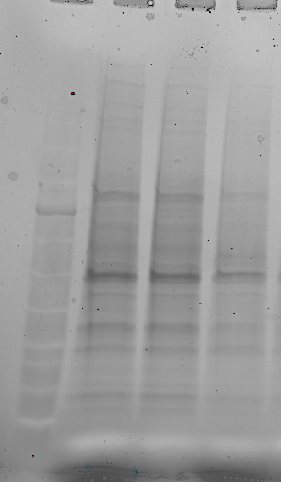 ABCG1  125kDa  63kDa  40kDa  Chemiluminescence  Stain free gel |
| --- |

**Figure S19: Full length Western blots and stain-free gels to accompany Figure 6(C).**

Representative full-length Western blot images are shown in their original, unprocessed form. Black boxes indicate the regions corresponding to the cropped panels presented in the main manuscript. Bands at ~40 kDa and ~63 kDa may represent degradation products or non-specific binding.

| Control  IgG  nIGFBP-6  Control  IgG  nIGFBP-6  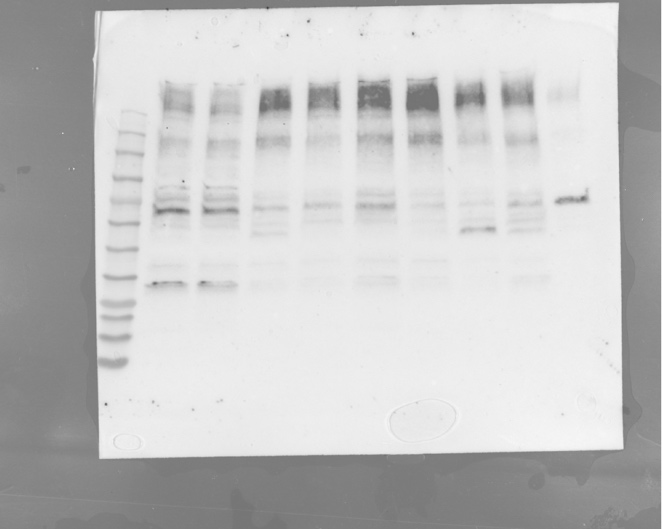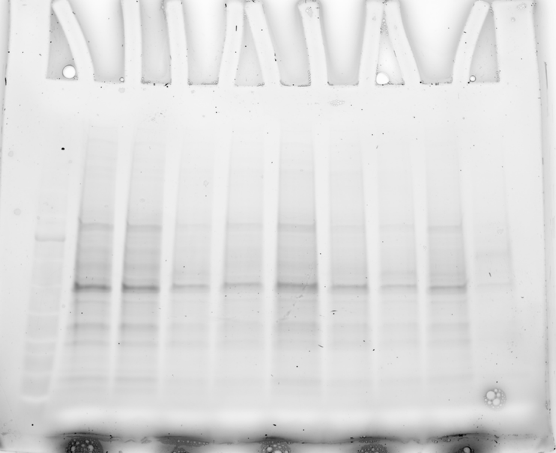  ABCA1  220kDa  Stain free gel  Chemiluminescence |
| --- |

**Figure S20: Full length Western blots and stain-free gels to accompany Figure 6(F).**

Representative full-length Western blot images are shown in their original, unprocessed form. Black boxes indicate the regions corresponding to the cropped panels presented in the main manuscript.


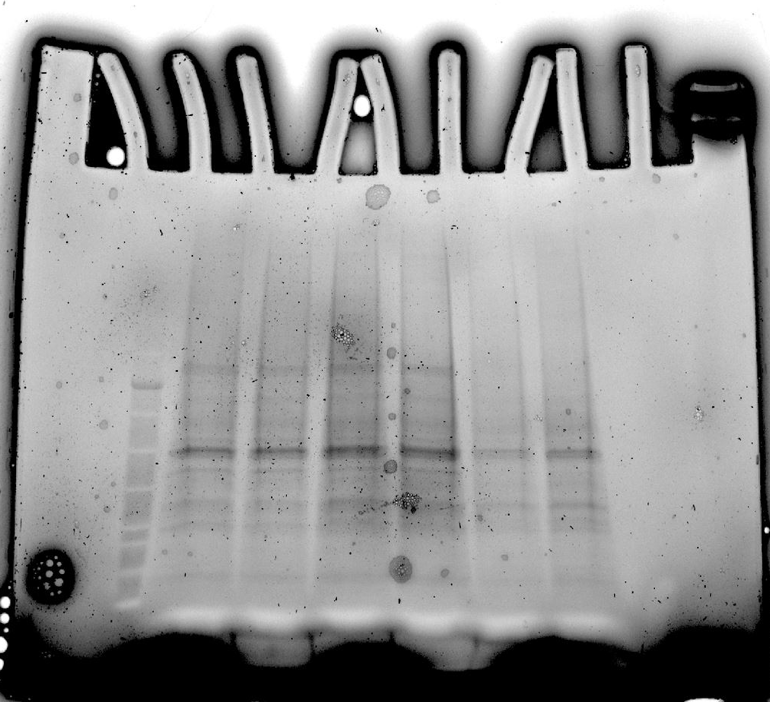


rIGFBP-6

| Control  rIGFBP-6  Control  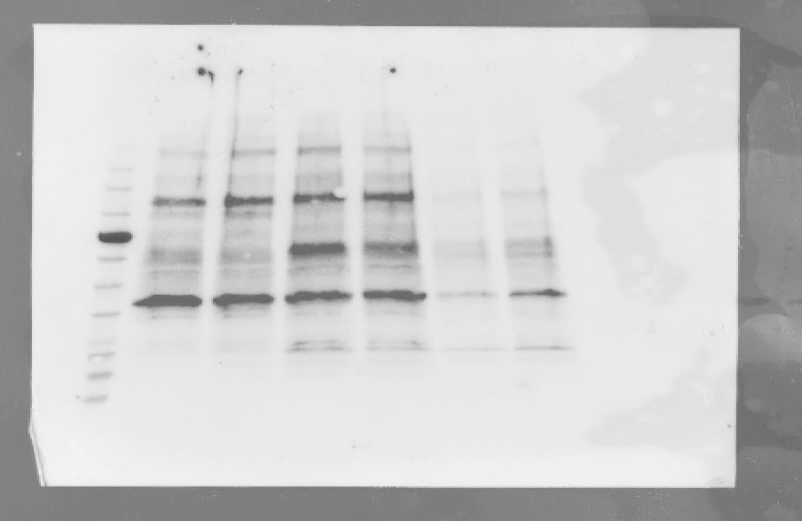  ABCG1  125kDa  63kDa  40kDa  Chemiluminescence  Stain free gel |
| --- |

**Figure S21: Full length Western blots and stain-free gels to accompany Figure 6(I).**

Representative full-length Western blot images are shown in their original, unprocessed form. Black boxes indicate the regions corresponding to the cropped panels presented in the main manuscript. Western blot analysis of foam cell lysates revealed that treatment with recombinant IGFBP-6 (rIGFBP-6) increased ABCG1 protein expression. This was most evident at the expected ~125 kDa band, corresponding to full-length ABCG1, and was confirmed by densitometric analysis showing a statistically significant increase following rIGFBP-6 treatment. Additional bands at ~63 kDa and ~40 kDa, likely representing isoforms or proteolytic fragments, also showed increased intensity in the rIGFBP-6-treated samples, although these changes were not statistically significant.

rIGFBP-6


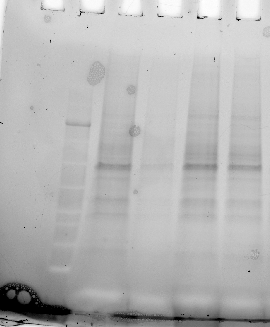


Control

rIGFBP-6

| Control  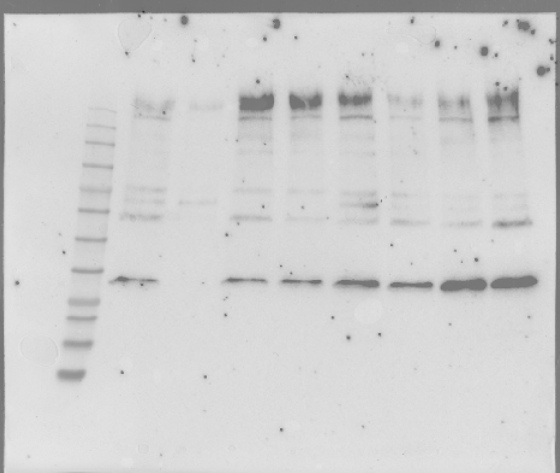  ABCA1  220kDa  Stain free gel  Chemiluminescence |
| --- |

**Figure S22: Full length Western blots and stain-free gels to accompany Figure 6(L).**

Representative full-length Western blot images are shown in their original, unprocessed form. Black boxes indicate the regions corresponding to the cropped panels presented in the main manuscript.

| 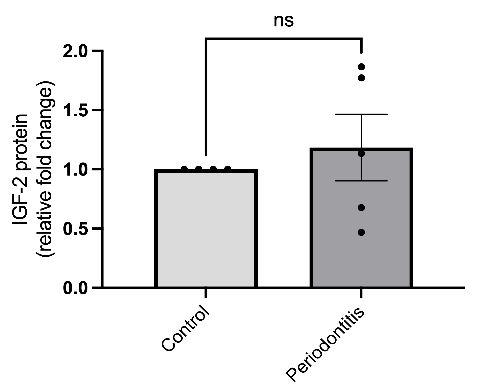  (A) | 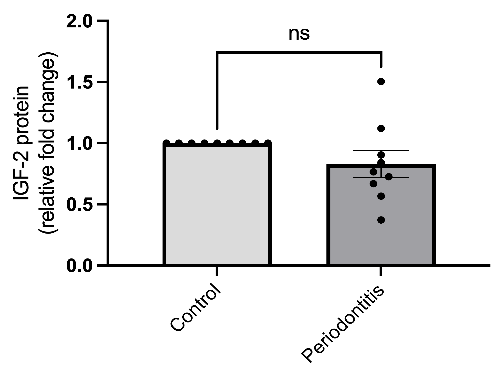  (B) |
| --- | --- |
| (C)  Periodontitis  Control  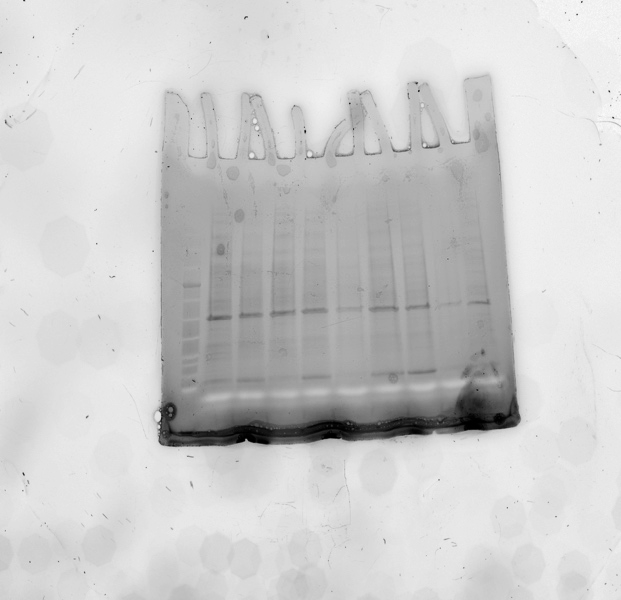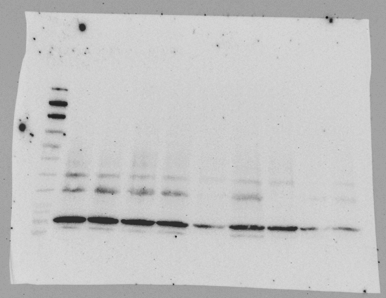  Stain Free  IGF-2 ~20kDa | Control  (D)  Periodontitis  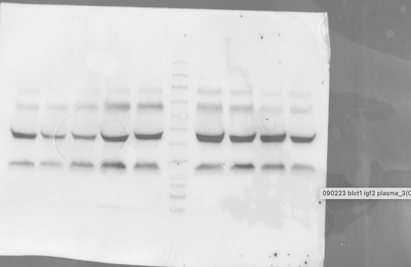  IGF-2 ~30kDa  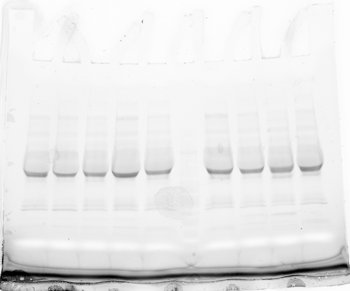  Stain Free |
| Chemiluminescence 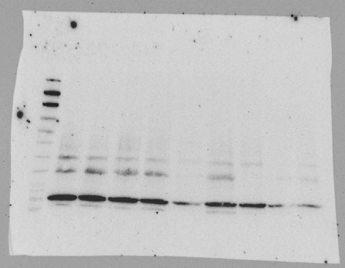 IGF-2  ~20kDa 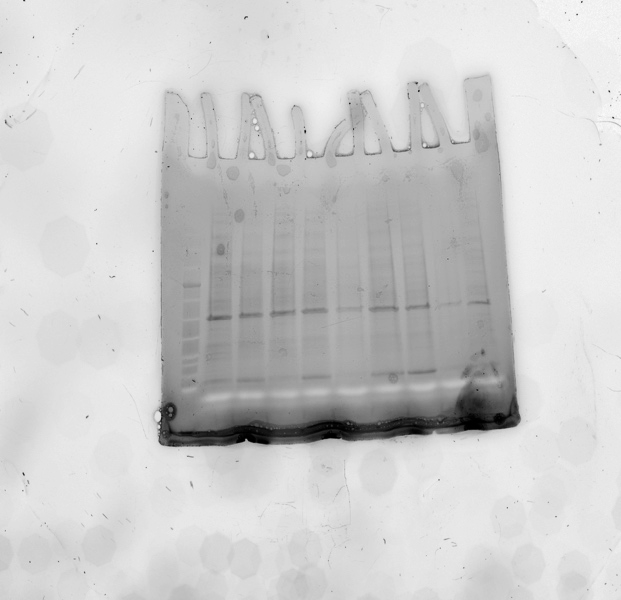 Stain free gel  (E) 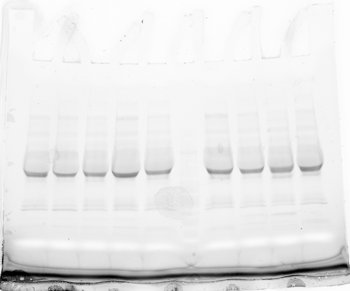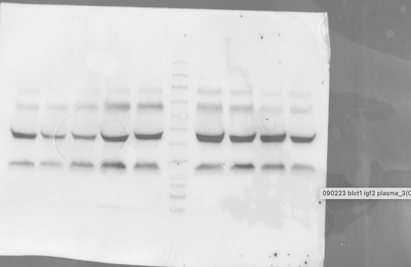 IGF-2  ~30kDa  Stain free gel  Chemiluminescence  (F) | |

**Figure S23: Comparable levels of IGF-2 protein in naïve monocytes and plasma from individuals with periodontitis and healthy controls.**

IGF-2 protein abundance was assessed in both **(A)** naïve monocytes and **(B)** plasma to determine whether altered IGFBP-6 expression was accompanied by changes in its high-affinity ligand. No significant differences in IGF-2 levels were observed between periodontitis and control groups in either sample type (monocytes: p = 0.6825; plasma: p = 0.1404). Protein quantification was performed by densitometry of Western blots, normalised using stain-free gel technology as a loading control, and expressed as fold change relative to controls. ns, no significant difference (two-tailed unpaired t-test). Error bars represent SEM. Representative Western blots for IGF-2 and corresponding stain-free gel images are shown for **(C)** naïve monocytes and **(D)** plasma. Representative full length Western blot images are shown in their original, unprocessed form for **(E)** naïve monocytes and **(F)** plasma, with black boxes corresponding to cropped samples in panels (C-D). Arrowheads indicate the detected IGF-2 bands detected at ~20kDa in monocytes and ~30kDa in plasma, consistent with reported IGF-2 proteoforms. Naïve monocyte samples included individuals with periodontitis (n=6; males=2, females=3, undeclared=1; mean age = 39.8 ± 14.62 years) and controls (n=4; males=3, females=1; mean age = 38.25 ± 13.57 years; p=0.7302, Mann–Whitney test). Plasma samples included individuals with periodontitis (n=9; males=3, females=5, undeclared=1; mean age = 36.89 ± 11.54 years) and controls (n=9; males=4, females=5; mean age = 33.22 ± 11.69 years; p=0.5126, unpaired t-test).

| (A)  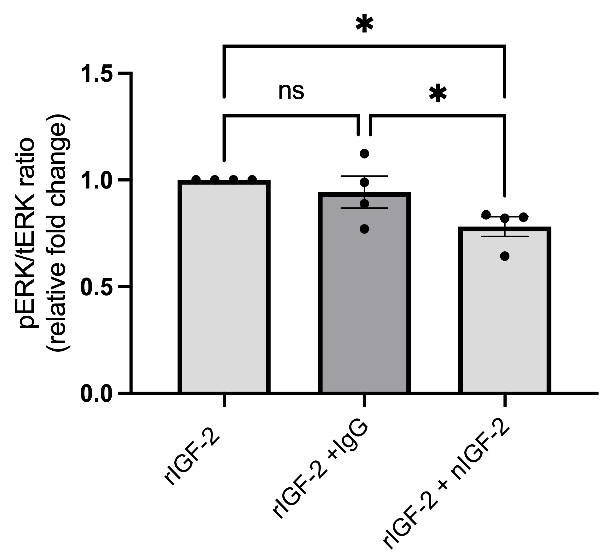 |
| --- |
| p-ERK1/2  Stain free  rIGF-2  rIGF-2  + IgG  rIGF-2  + nIGF-2  42 kDa  44 kDa  t-ERK1/2  44 kDa 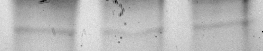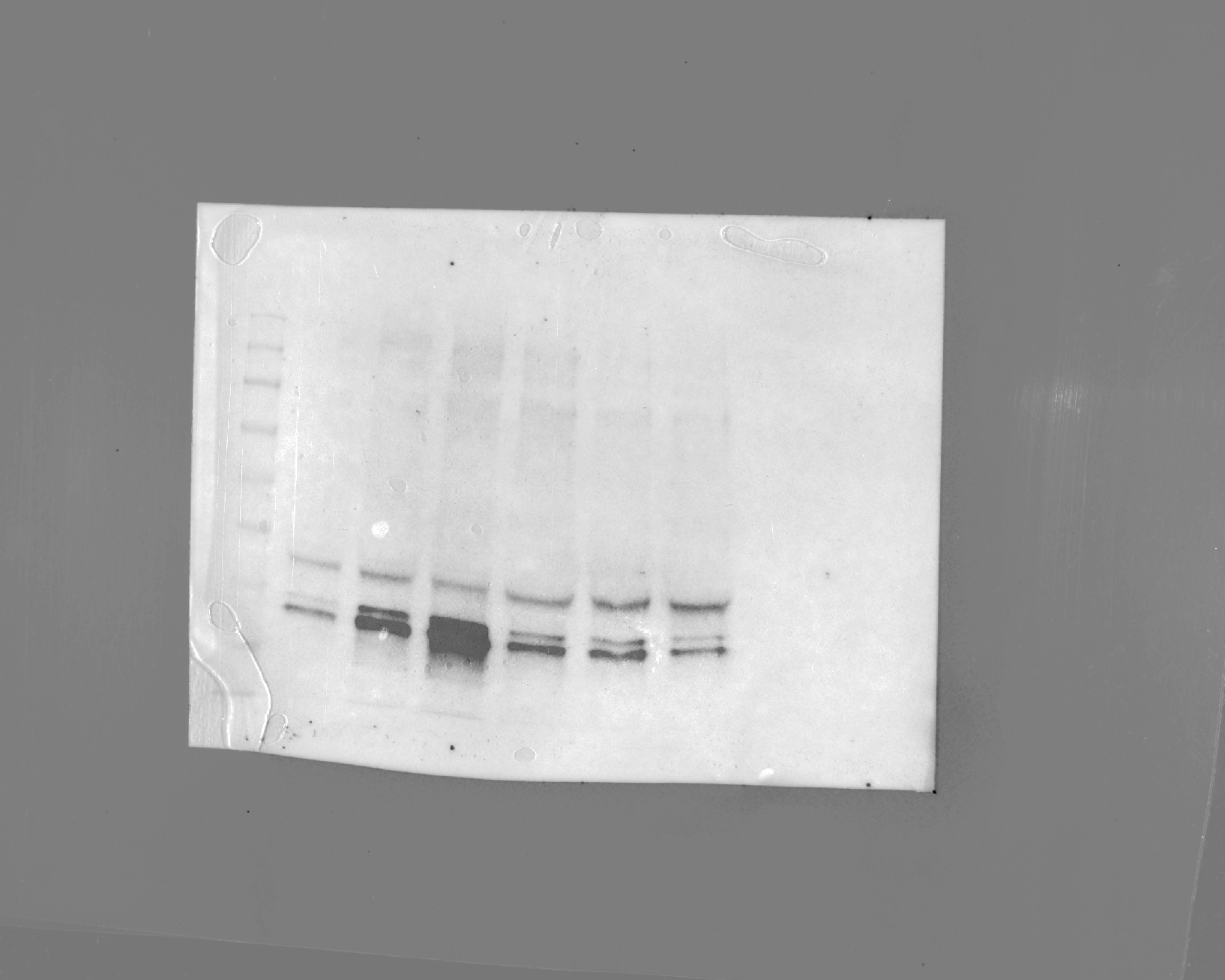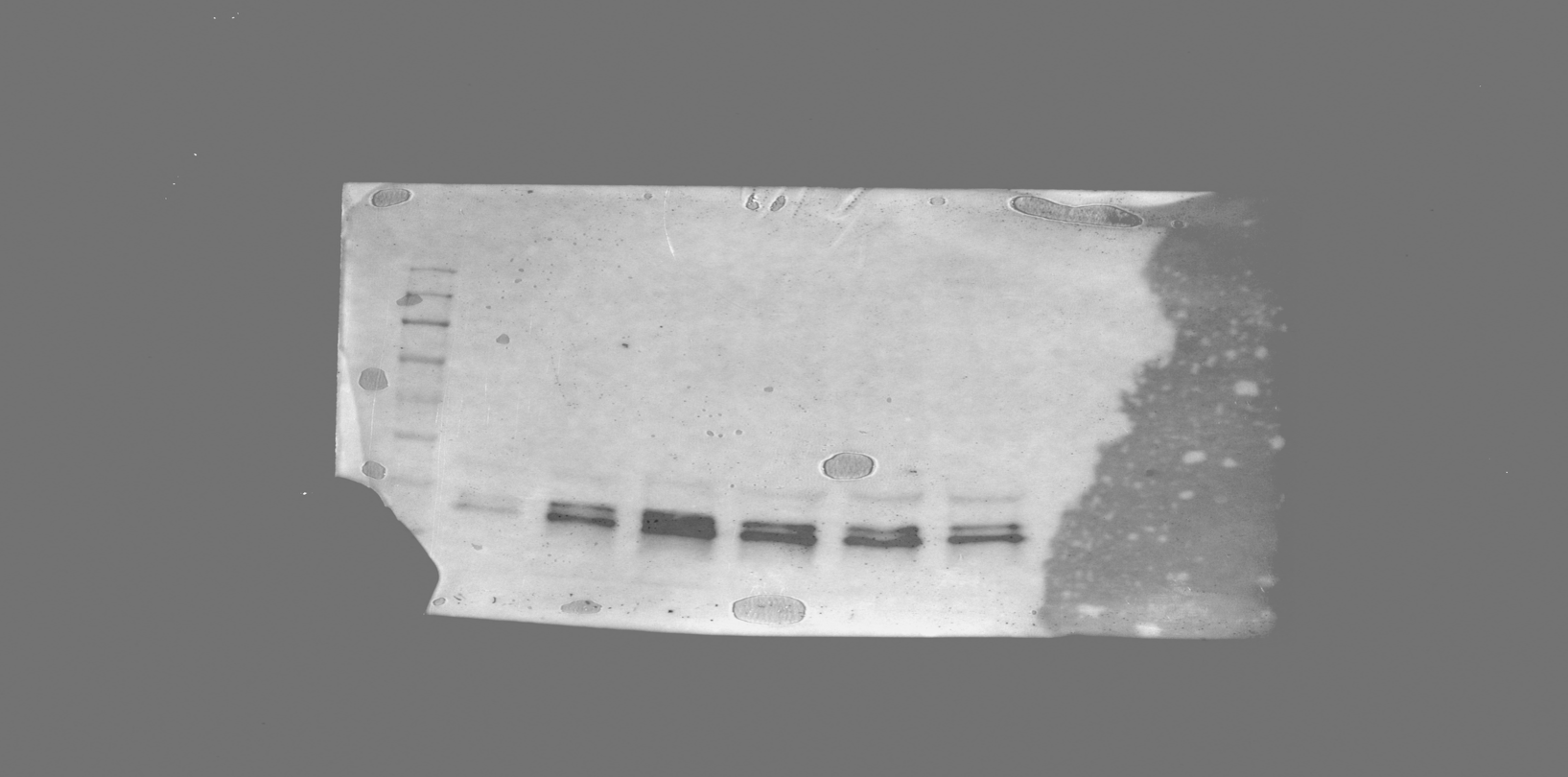 42 kDa  (B) |
| (C) 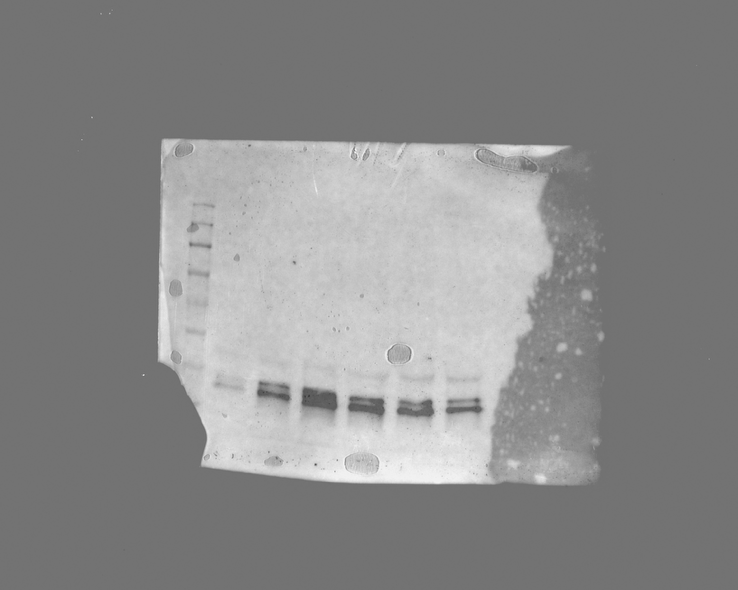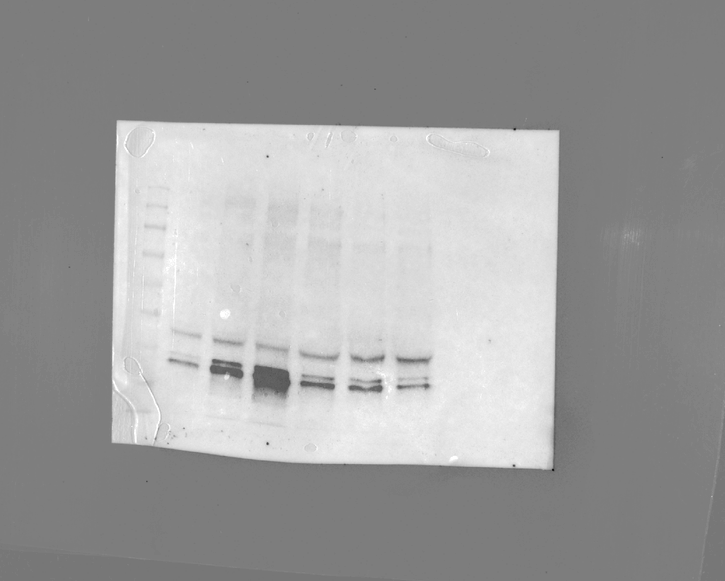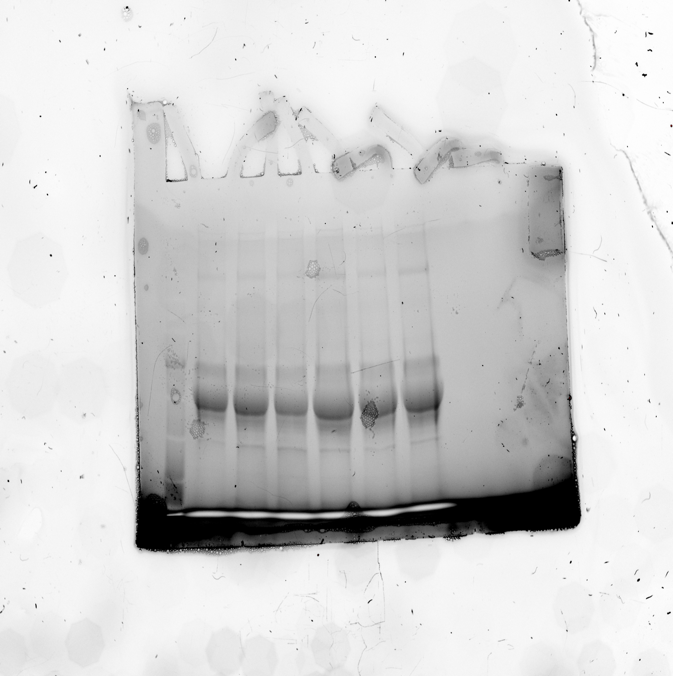 Chemiluminescence  Chemiluminescence  Stain free gel  t-ERK1/2  44 kDa  42 kDa  42 kDa  44 kDa  p-ERK1/2 |

**Figure S24: Validation of IGF-2 (nIGF-2) neutralising antibody in macrophages.**

To validate the efficacy of the IGF-2 neutralising antibody, macrophages derived from naïve monocytes of healthy individuals (n = 4) were treated with 20 ng/ml recombinant IGF-2 (rIGF-2) in the presence or absence of 5 μg/ml neutralising IGF-2 antibody (nIGF-2) or IgG control. Cells were lysed after 30 min and analysed for ERK1/2 activation. **(A)** The ratio of phosphorylated ERK1/2 (pERK1/2) to total ERK1/2 (tERK1/2) was calculated following densitometric quantification of Western blot bands, normalised using stain-free gel technology as a loading control. Data are expressed as fold change relative to rIGF-2-treated control. ns, not significant, * p < 0.05, using repeated measures ANOVA with Student–Newman–Keuls *post hoc* test. Error bars indicate SEM.**(B)** Representative Western blots for pERK1/2 and tERK1/2, with corresponding stain-free gel images.**(C)** Representative full-length Western blot images are shown in their original, unprocessed form. Black boxes indicate the regions corresponding to the cropped panels presented in panel (B). Arrowheads indicate detected bands; molecular weight markers are shown in kDa.

| 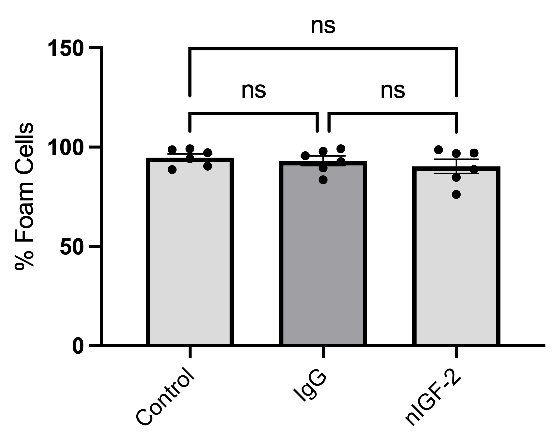  (A) No. of foam cells | 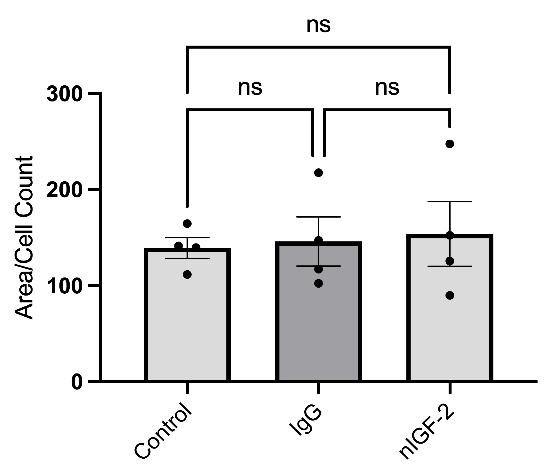  (B) Fluorescent microscopy |
| --- | --- |
| MERGE 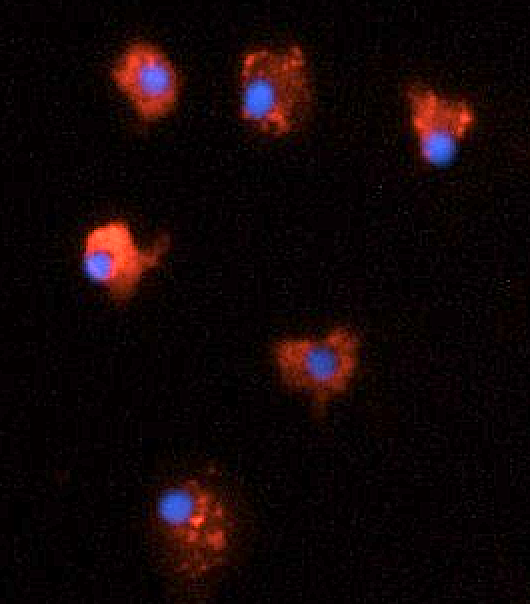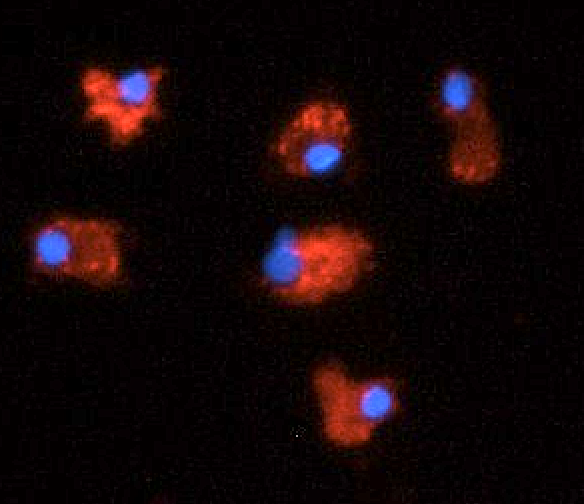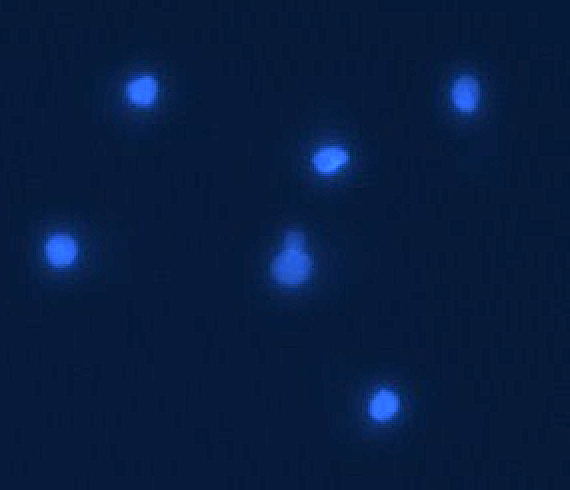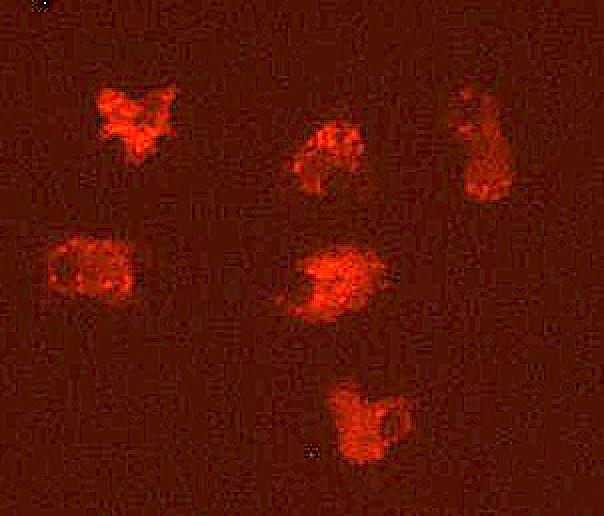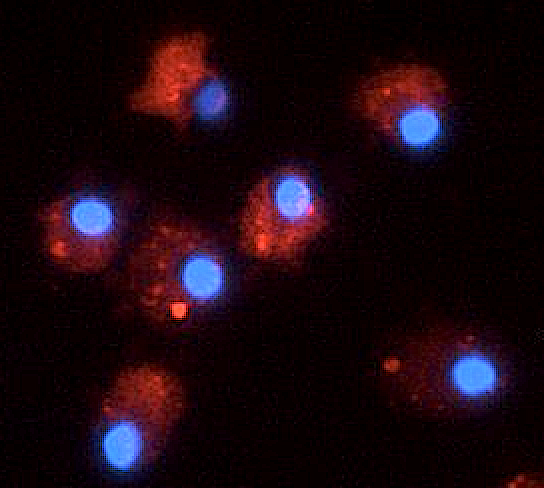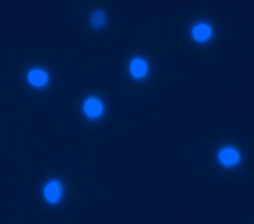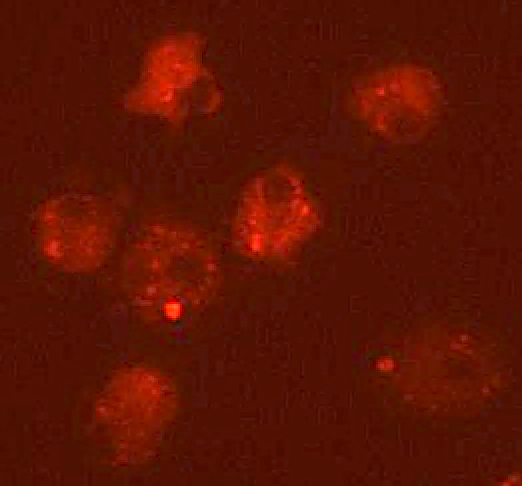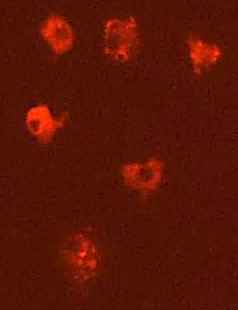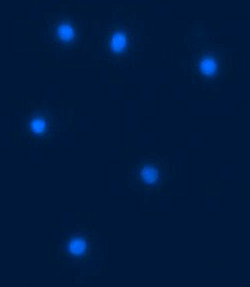 Control  IgG  nIGF-2  DAPI  oxLDL  (C) | |

**Figure S25: Foam cell formation was not affected by neutralising IGF-2 in macrophages from healthy individuals.**

Macrophages from individuals with periodontitis were treated with 10 μg/ml Dil-labelled oxLDL in the presence of 5 μg/ml neutralising IGF-2 antibody (nIGF-2) or 5 μg/ml IgG control for 24 h. (A) Foam cell number was quantified by calculating the percentage of Dil oxLDL (red) cells relative to total DAPI (blue) nuclei per field (p=0.2505; n=6), with ~250 cells counted per condition. (B) Intracellular Dil oxLDL was quantified as red fluorescent area per cell across 10 fields using ImageJ (p=0.9306; n=4). (C) Representative fluorescence microscopy images of foam cells showing nuclei stained with DAPI (blue) and Dil oxLDL (red). ns, not significant, using repeated measures ANOVA with Student–Newman–Keuls post hoc test (lipid area) and Friedman’s test with Dunn’s post hoc correction (foam cell number). Error bars indicate SEM. These results suggest that the enhanced lipid accumulation observed following IGFBP-6 neutralisation is independent of IGF-2 signalling.

(C)

(A)

(B)


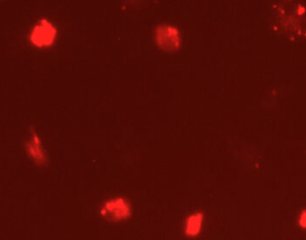

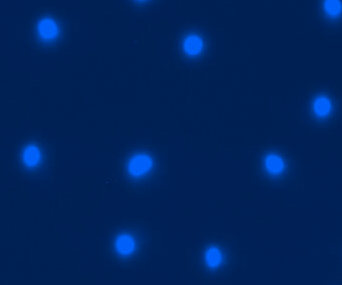


DAPI

oxLDL

MERGE

Brightfield

oxLDL

MERGE

(D)

(E)

(F)

|  |
| --- |

Figure S26: Quantification of foam cell number and foam cell size by cell area measurement.

Foam cell number was determined by calculating the percentage of DiI-oxLDL–positive (red) cells relative to total DAPI-stained (blue) nuclei per field, with ten independent fields of view analysed per condition. **(A)** DAPI-stained nuclei (blue). **(B)** DiI-oxLDL fluorescence (red) identifying lipid-positive cells. **(C)** Merged image showing representative DiI-oxLDL–positive foam cells (arrows) and DiI-oxLDL–negative cells used for manual counting **(D)** Brightfield images showing cell morphology and boundaries. **(E)** DiI-oxLDL fluorescence (red). **(F)** Merged image with manual delineation of DiI-oxLDL–positive cells (yellow outlines) using Fiji (ImageJ) for quantification of cell area (µm²); five independent fields of view were analysed per condition for size measurements. Representative images are shown. Scale bars represent 20 μm (A–C) and 200 μm (D–F).
